# American zebra optimization algorithm for global optimization problems

**DOI:** 10.1038/s41598-023-31876-2

**Published:** 2023-03-30

**Authors:** Sarada Mohapatra, Prabhujit Mohapatra

**Affiliations:** Vellore Institute of Technology, Vellore, Tamil Nadu 632014 India

**Keywords:** Applied mathematics, Computational science, Computer science

## Abstract

A novel bio-inspired meta-heuristic algorithm, namely the American zebra optimization algorithm (AZOA), which mimics the social behaviour of American zebras in the wild, is proposed in this study. American zebras are distinguished from other mammals by their distinct and fascinating social character and leadership exercise, which navies the baby zebras to leave the herd before maturity and join a separate herd with no family ties. This departure of the baby zebra encourages diversification by preventing intra-family mating. Moreover, the convergence is assured by the leadership exercise in American zebras, which directs the speed and direction of the group. This social lifestyle behaviour of American zebras is indigenous in nature and is the main inspiration for proposing the AZOA meta-heuristic algorithm. To examine the efficiency of the AZOA algorithm, the CEC-2005, CEC-2017, and CEC-2019 benchmark functions are considered, and compared with the several state-of-the-art meta-heuristic algorithms. The experimental outcomes and statistical analysis reveal that AZOA is capable of attaining the optimal solutions for maximum benchmark functions while maintaining a good balance between exploration and exploitation. Furthermore, numerous real-world engineering problems have been employed to demonstrate the robustness of AZOA. Finally, it is anticipated that the AZOA will accomplish domineeringly for forthcoming advanced CEC benchmark functions and other complex engineering problems.

## Introduction

Optimization is the process of identifying the decision variables while maintaining various constraints to maximize or minimize the cost function. The constraints, cost function, and design variables are the critical components of any optimization problem. Optimization techniques are widely applicable in the fields of engineering^1^, feature selection^2,3^, tuning of machine learning parameters^4^, wireless sensor networks^5^, image processing^6^, and bioinformatics^7^. Most real-life problems are highly non-convex and non-linear due to the presence of multiple design variables and the intrinsic nature of the constraints. Furthermore, there is no certainty of obtaining a global optimal solution^8^. The challenges connected with these real-life problems inspire scientists to design novel and successful strategies for better outcomes. The optimization approaches may be categorized into two types, such as gradient-based deterministic approaches and stochastic-based non-traditional approaches^9^. The deterministic-based approaches have limitations in solving problems with discontinuous search spaces, non-convex, high-dimensional, and non-differentiable objective functions. However, the stochastic-based strategies do not practice gradient-based information; instead, they are intelligent enough to overcome the limitations by relying on random methods in the search space. The meta-heuristic algorithms are prevalent for their broad applicability among the various techniques in stochastic-based approaches. The meta-heuristic algorithms have a high potential for exploring the solution space and exploiting the best optimal solution. Therefore, several researchers have attempted not only to propose novel meta-heuristic algorithms but also to enhance the efficiency of existing methods, resulting in the conception of several novel meta-heuristics during the last few decades. In general, meta-heuristic algorithms may be grouped into three major types, such as evolutionary algorithms (EA), natural phenomenon (NP)-based algorithms, and swarm intelligence (SI) algorithms^10,11^. Evolutionary algorithms (EAs) mimic Darwin's evolution process using three mechanisms: selection, reproduction, and mutation. Some of the most prominent EAs are Differential Evolution (DE)^12^, Genetic Algorithm (GA)^13^, Covariance Matrix Adaptation Evolutionary Strategy (CMA-ES)^14^, Evolutionary Strategy (ES)^15^, History-based Adaptive DE Variants with Linear Population Size Reduction (L-SHADE)^16^, Biogeography-Based Optimizer (BBO)^17^, and Learner Performance based—Behaviour (LPB)^18^. The NP-based algorithms emulate the chemical and physical laws of the cosmos. Most of the well-known algorithms based in this category are Simulated Annealing (SA)^19^, Central Force Optimization (CFO)^20^, Gravitational Search Algorithm (GSA)^21^, Water Cycle Optimizer (WCO)^22^, Black Hole Algorithm (BHA)^23^, Lightning Search Algorithm (LSA)^24^, Multi-Verse Optimization (MVO)^25^, Thermal Exchange Optimization (TEO)^11^, Henry Gas Solubility Optimization^26^, Equilibrium Optimizer (EO)^27^, Archimedes Optimization Algorithm (AOA)^28^, Lichtenberg Algorithm (LA)^29^, Flow Direction Algorithm (FDA)^30^, and Fusion–Fission Optimization (FuFiO)^31^. Swarm Intelligence (SI) algorithms follow the natural behaviour of mammals, birds, and insects. Most of the popular SI-based algorithms are Particle Swarm Optimizer (PSO) algorithm^32^, Gray Wolf Optimizer (GWO)^33^, Elephant Herding Optimization (EHO)^34^, Moth Flame Optimization (MFO)^35^, Whale Optimization Algorithm (WOA)^36^, Salp Swarm Algorithm (SSA)^37^, Grasshopper Optimizer Algorithm (GOA)^38^, Harris Hawks optimization (HHO)^39^, An Improvised Competitive Swarm Optimizer (ICSO)^40^, Tunicate Swarm Algorithm (TSA)^41^, Levy Flight Distribution (LFD)^10^, and American Vultures Optimization Algorithm (AVOA)^42^, Aquila Optimizer (AO)^43^, Golden Eagle Optimizer (GEO)^44^, Orca Predation Algorithm (OPA)^45^, and Artificial Rabbits Optimization (ARO)^46^, Artificial Gorilla Troops Optimizer (GTO)^47^, Mountain Gazelle Optimizer (MGO)^48^. It is emphatic to state that the existing meta-heuristics^49^ have advantages and limitations. For example, the classical PSO algorithm has the weakness of premature convergence in high-dimensional search space, whereas the genetic algorithm has difficulties in parameter tuning and extensive computation. Similarly, the gravitational search algorithm has the shortcoming of a slow convergence rate and the presence of many control parameters. The eminent GWO algorithm has difficulty tackling challenging engineering problems due to its low local search capability. Also, the recently proposed TSA algorithm has the incapability of addressing multimodal problems with large dimensions. Therefore, it is essential to challenge these limitations by adapting new techniques and methodologies. Furthermore, the "No Free Lunch (NFL) Theorem"^50^ states that no algorithm can be considered the best optimizer for all optimization problems. The unsolved problems also need a scarce approach to obtain solutions. As a result, pioneering meta-heuristics are needed to be offered frequently by investigators around the world. Hence, in this paper, a novel meta-heuristic inspired by the social behaviour of American Zebras, namely the American Zebra Optimization Algorithm (AZOA), is being projected. American zebras are socially adept animals that stay in a group with a male, several females, and offspring^51^. The foremost behaviours of zebras include feeding, mating, preserving social hierarchy, and guiding the youngsters^52,53^. American zebras are distinguished from other mammals by their unique and fascinating character “honesty”. The social character “honesty” navies the baby zebras to leave the herd before maturity and join a separate herd with no family relation. This departure of the baby zebra balances diversification by preventing intra-family mating. Moreover, the matured male zebra in the group charms the female zebra to persuade the convergence. This scarcest concept of social accordance inspires us to propose the American Zebra Optimization Algorithm (AZOA). It is anticipated that the effortlessness and robustness of the AZOA algorithm will propel rapid and accurate global solutions while solving benchmark functions and real-life engineering problems. The main contributions of this study are highlighted as follows:

- A novel bio-inspired algorithm, namely the American zebra optimization algorithm (AZOA) is proposed and inspired by the unique social behaviour and leadership exercise of American zebras.
- The various social behaviour of AZOA is introduced and modelled mathematically in five simple phases for easy implementation and superior performance.
- AZOA is implemented and tested on CEC-2005, CEC-2017, and CEC-2019 benchmark test functions and several engineering design problems to ensure the robustness of the proposed algorithm.

The rest part of the paper is organized as follows: Sect. 2 reviews the related works. Section 3 discusses the motivation and the mathematical modelling of the proposed work. Section 4 presents the experimental setup and result discussions. Section 5 focuses on the application of AZOA to classical engineering problems. Finally, Sect. 6 provides the conclusions and recommendations for future research work.

## Related works

In the literature, metaheuristic algorithms are classified into various categories. Despite distinct classifications, one could claim that the majority of these algorithms have been inspired by the collective behaviour and hunting techniques of animals in the wild. This section looks at metaheuristic algorithms that are inspired by nature and studies the basic algorithms that have been proposed to solve optimization problems. Genetic algorithm (GA) is the earliest and most widely used approach for addressing optimization problems that Holland proposed in 1992, motivated by Darwinian evolutionary principles. This algorithm has been employed extensively in the majority of optimization problems involving two recombination and mutation operators and is regarded as one of the most popular algorithms^54^, with numerous enhanced and recombination variants already described^55^. Particle swarm optimization (PSO) was proposed in 1995 based on the swarming behaviour of birds, fish, and other animals in nature^32^. It has been implemented in nearly all optimization fields, including computational intelligence, design, and planning applications. However, many researchers still propose a large number of variants to improve the performance of the PSO algorithm. In order to improve the diversity accuracy and avoid the low local optimum of PSO, Zaman et al.^56^ proposed an improved PSO with BSA called PSOBSA. Farmland Fertility Algorithm (FFA)^57^ has been developed to tackle ongoing issues; it was motivated by the fact that farmland is separated into many sections, with each sector's solutions becoming optimised for optimal efficiency, both in internal and external memory. Simulation findings reveal that farmland fertility often performs better than other metaheuristic algorithms. In reference^58^, Farhad Soleimanian Gharehchopogh et al. enhanced the FFA to apply it to tackle the TSP problem. It measures the quality of every portion of their farms throughout their visit and enhances soil quality by employing fertilisers and organic materials. Harris Hawks Optimizer (HHO) is a well-known animal behavior-based algorithm; the cooperative behaviour and pursuit style of Harris' hawks in nature, known as surprise pounce, is the primary inspiration for HHO^59^. Kaur et al. presented the TSA algorithm as being motivated by replicating the lifestyle of tunicates at sea and how food is delivered by Satnam^41^. In addition, it is regarded as one of the newest metaheuristic algorithms for engineering optimization issues. Tunicate can explore for a food source, although they are unaware of its location. Even though the TSA algorithm is simple and works well, it is easy to get stuck in local optimization, which makes it converge faster than some metaheuristic algorithms. So, Farhad Soleimanian Gharehchopogh^60^ introduced a version of this algorithm called the QLGCTSA algorithm to address these issues. Li et al.^61^ proposed a slime mould algorithm (SMA) that mimics the slime mould’s diffusion and foraging behaviour. It has a number of new features and a special mathematical model that simulates the biological wave using adaptive weights. It offers an optimum route for linking food with a high capacity for exploration and exploitation. The results indicate that the proposed SMA has a competitive and frequently excellent performance on various search landscapes. The Tree-Seed Algorithm (TSA) was proposed by Kiran in 2015 for the resolution of continuous optimization problems and is inspired by the relation between trees and seeds in nature, as well as how tree seeds grow and position themselves^62^. Xue et al.^63^ proposed a sparrow search algorithm (SSA) based on the group wisdom, foraging, and anti-predation behaviours of sparrows. The cuckoo search (CS) algorithm was proposed by Xin-She Yang and Suash Deb in 2009, and was inspired by the aggressive brood parasitism and egg-laying behaviours of certain cuckoo species^64^. However, CS algorithms have problems such as premature convergence, delayed convergence, and getting trapped in the local trap. In order to overcome this problem, Shishavan, Saeid Talebpour et al.^65^ proposed an improved Cuckoo Search Optimization (CSO) algorithm with a Genetic Algorithm (GA) for community detection in complex networks. Symbiotic Organisms Search (SOS)^66^ is a new, robust, and powerful metaheuristic algorithm inspired by the symbiotic interaction strategies adopted by organisms to survive and propagate in the ecosystem. In reference^67^, Hekmat Mohammadzadeh et al. introduced a Feature Selection with Binary Symbiotic Organisms Search Algorithm for Email Spam Detection.

### Ethical approval

This article does not contain any studies with human participants or animals performed by any of the authors.

## American zebra optimization algorithm

This section highlights the inspiration of social-life style of American zebra in proposing the AZOA algorithm along with the mathematical formulation.

### Motivation

**Figure 1:**
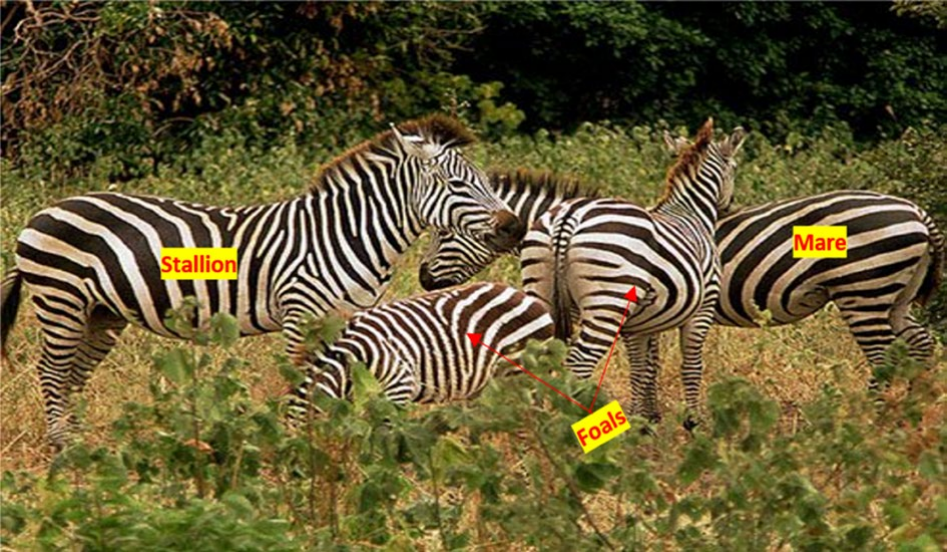
American zebras in a family group.

**Figure 2:**
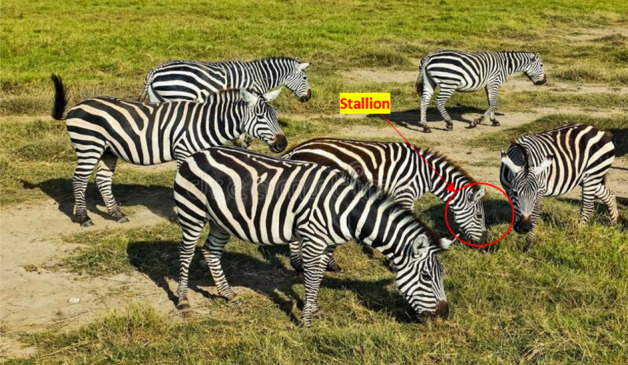
Grazing around the family leader (stallion).

The American Zebras belong to the family of Equidae with white and black striped coats. They live throughout the southeast area of America and are spotted in environments such as shrublands, plains, forests, and hilly places. The stripes of American Zebras appear in distinct shapes for every individual. The American zebras are about 7.5 ft. in body length with a shoulder height of 4 ft. and a weight of 600 lb. They have good vision, strong hearing, and the capability to run at a speed of 25 miles per hour. The zebras are social instinct animals that live in a family group, including a male zebra, several females, and offspring, as shown in Fig. 1. They spend time in herds, groom one another, and to get fresh grass, they graze around the family leader stallion, as shown in Fig. 2. The zebras strictly follow the social limitations and do not mate with their family members. The mature stallion zebras live in a single group to find a suitable mating partner, whereas the female foals join other groups. The male zebras join the solitary groups once they are old enough to breed, while the female zebras depart from their parent groups before reaching adolescence. This process of leaving the group prevents the zebra parents from breeding with their offspring to guarantee the required diversity in AZOA. Similarly, the convergence is assured by the leadership exercise in American zebras to direct the speed and direction of the group^68^. The group must be guided to the best available water reserves by the stallion group leader. The stallion dominates the other group of zebras by bringing the group members to utilize water sources. This social lifestyle of the zebras is indigenous in nature and extremely fruitful for proposing a meta-heuristic technique. Hence, based on this source of inspiration, a novel meta-heuristics algorithm called AZOA is being developed along with its mathematical formulation to accomplish the global optimization challenges.

### Mathematical modelling

This section presents the mathematical modelling of the social life behaviour of American zebras in proposing the AZOA algorithm. The life activity of American Zebras consists of 5 key phases, which are listed as the following:

- Phase 1: Formation of random zebra groups
- Phase 2: Feeding activity of American zebras
- Phase 3: Breeding activity of American zebras
- Phase 4: Group leadership
- Phase 5: Leadership transition stage of selecting a new leader

#### Phase 1: Formation of random zebra groups

**Figure 3:**
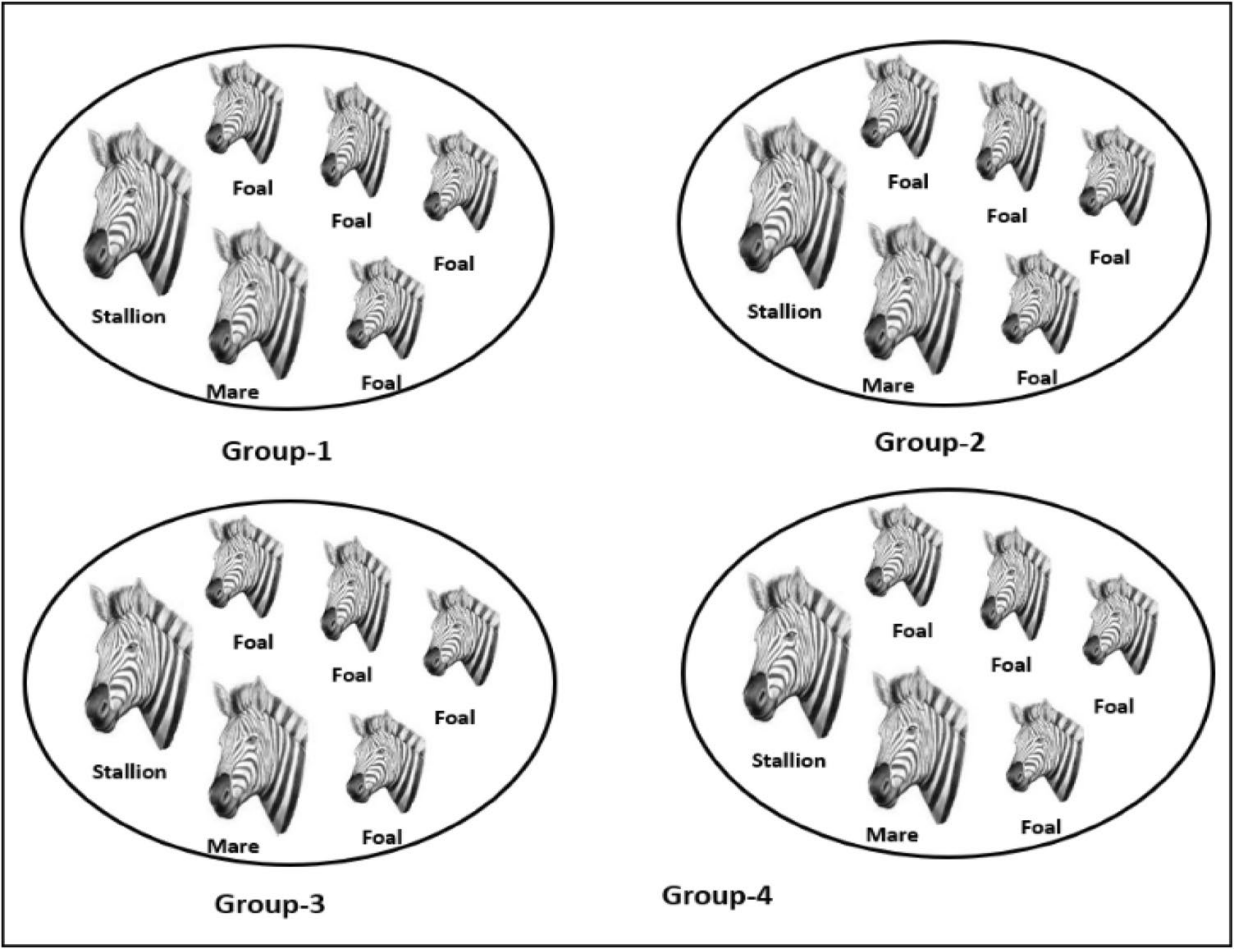
Formation of groups from original population.

In the wild, the zebras live in several different groups by following the group leader stallion, which seems to divide the whole population into multiple groupings. Here, the notation ‘*P*’ represents the stallion probability in the entire population ‘*S*’, and the total ‘*N*’ number of groups is calculated by the formula $N=S*P$. The position of ${i}$th zebra in ${j}$th group ${(Z}_{i,j\in N}=\left\{{Z}_{ij1},{Z}_{ij2}, {Z}_{ij3},.....,{Z}_{ijn}\right\})$ for $n$-dimensional search space is calculated using the formula ${Z}_{i,j}={(Z}_{max}-{Z}_{min})rand+{Z}_{min}$. Here, the upper and lower extreme points of the search area are defined by ${Z}_{max}$ and ${Z}_{min}$ respectively. The symbol ‘$rand$’ denotes a random value between [0, 1]. This mechanism ensures $N$ number of different zebra crowds with a unique stallion in each group. The sample image of division of zebra groups is reflected in Fig. 3.

#### Phase 2: Feeding activity of American zebras

Zebras are herbivores and depend mainly on various grass and green leaves. Getting fresh grass and green leaves is very difficult for young zebras, so they depend on the leader of the family. Hence, zebras always graze together and move around the family leader stallion. To mathematically model the feeding activity of American zebras, the following equations are proposed.

1 $$\overline{Z} _{i}^{j} = \left\{ {\begin{array}{*{20}l} {2R_{1} \sin \left( {2\pi R_{2} } \right) \times \left( {Z_{S}^{j} - Z_{i}^{j} } \right) + Z_{S}^{j} ,} & { if\; R_{3} < 0.5; } \\ {2R_{1} \cos \left( {2\pi R_{2} } \right) \times \left( {Z_{S}^{j} - Z_{i}^{j} } \right) + Z_{S}^{j} ,} & { otherwise,} \\ \end{array} } \right.\quad \forall \quad i \in N_{j}$$

2 $$Z_{i}^{j} = \left\{ {\begin{array}{*{20}l} {\overline{Z} _{i}^{j} ,} & {\overline{F} _{i}^{j} < F_{i}^{j} ;} \\ {Z_{i}^{j} ,} & {otherwise,} \\ \end{array} } \right.$$

where ${Z}_{S}^{j}$ and ${Z}_{i,}^{j}$ represent the position of the stallion and ${i}$th zebra of the ${j}$th group, respectively, ${N}_{j}$ represents the total members in the ${j}$th group, ${R}_{1}$ indicates a uniform random value between [− 2, 2] that induces the feeding of zebra at multiple angles of 360 degrees around leader of the group, ${R}_{2}$ denotes the adaptive parameter which is evaluated by Eq. (3), ${R}_{3}$ denotes a random value lies in [0, 1], the $\mathrm{Sin}$ and $\mathrm{Cos}$ function help the movement of other ${i}$th members in multiple angles around leader of the family^69^, ${\overline{Z} }_{i}^{j}$ represents the new update ${i}$th member position while feeding, and lastly, ${\overline{F} }_{i}^{j}$ is its fitness value of ${i}$th zebra.

3 $$R_{2} = 1 - t \times \left( \frac{1}{T} \right)$$

Here, $T$ and $t$ denote the maximum iteration and current iteration respectively.

#### Phase 3: Breeding activity of American zebras

**Figure 4:**
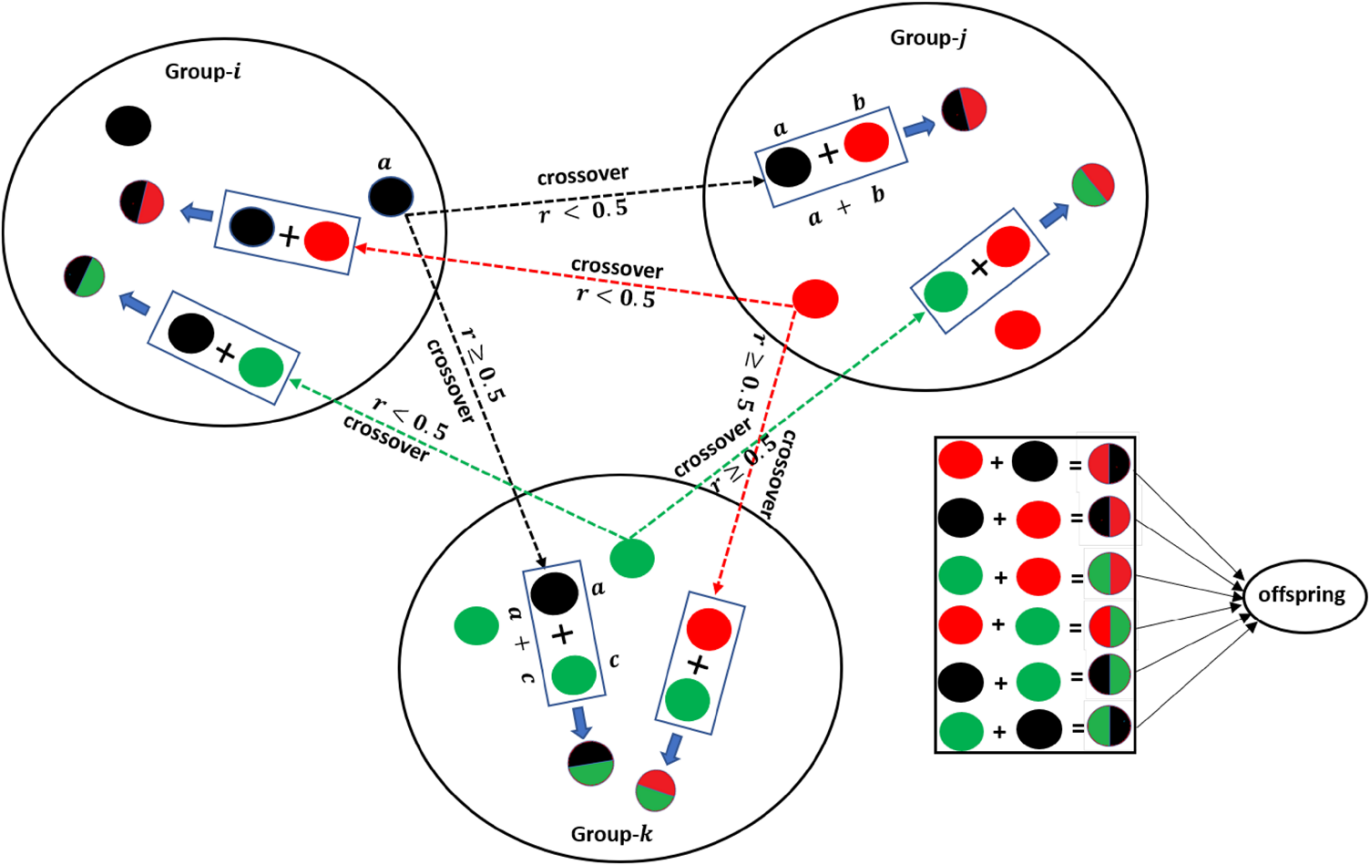
Breeding activity of American zebras.

For the proper balance of the food chain, the presence of animals at the bottom of the food chain, such as horses, cows, donkeys, and zebras, in abundance is essential. Hence, these animals reproduce profusely. Among these animals, the behaviour of the zebra is completely different, and it preserves the dignity of the family. They do not breed with their parents and siblings. Hence, the young zebras leave their families before adulthood and join another zebra family for breeding. This mechanism is presented graphically in Fig. 4 by considering three different zebra groups. Here, the baby zebra of the ${i}$th group has two ways to choose the new family; that is, the baby zebra can go to the ${j}$th group or the ${k}$th group. Similarly, other baby zebras of each group are to choose such a new group as if none of their brothers and sisters has ever been there. Since these baby zebras have no family ties in their new group, they breed without any restriction. Thus, the baby zebras from $j$ and $k$ identify other groups and breed there. In this process, the overall decency of the family is preserved, which helps to maintain diversity in the AZOA algorithm. To model the zebras' breeding activity, the following equations have been developed.

4 $$Z_{j}^{q} = {\text{Crossover}}\left( {Z_{i}^{a} ,Z_{j}^{b} { }} \right)\;if{\text{ r}} < {\text{pc }},{ }i \ne j$$

5 $$Z_{k}^{q} = {\text{Crossover}}\left( {Z_{i}^{a} ,Z_{k}^{c} { }} \right)\;if{\text{ r}} \ge {\text{pc }},{ }\;i \ne k\quad \forall i, j, k \in N$$

where ${Z}_{i}^{a}$ represents position of the baby zebra $a$ from ${i}$th group, ${Z}_{j}^{b}$ denotes position of zebra $b$ from ${j}$th group*,* ${Z}_{k}^{c}$ represents position of the zebra $c$ from ${k}$th group, and ${Z}_{j}^{q}$ and ${Z}_{k}^{q}$ are the position of zebra $q$ in ${j}$th group and ${k}$th group, respectively.

#### Phase 4: Group leadership

Zebras give great importance to the leader of the family. The leader of the family searches for green grasslands, tree leaves, and water bodies for them. The leader often fights off other rival zebras and provides good food and drink for his family. The group of zebras, which is stronger than the other group, retains the rights over the water reservoir and the grasslands. After that, others can take advantage of it. This approach is modelled using the following equations.

6 $$\overline{Z} _{S}^{j} = \left\{ {\begin{array}{*{20}l} {2R_{4} {\text{ sin}}\left( {2\pi R_{5} } \right) \times \left( {WR - { }Z_{S}^{j} } \right) + WR ,} & {if\; R_{6} < 0.5;} \\ {2R_{4} {\text{ cos}}\left( {2\pi R_{5} } \right) \times \left( {WR - { }Z_{S}^{j} } \right) + WR ,} & {otherwise, } \\ \end{array} } \right.$$

7 $$Z_{S}^{j} = \left\{ {\begin{array}{*{20}l} {\overline{Z} _{S}^{j} , \overline{F} _{S}^{j} < F_{S}^{j} ;} \\ {Z_{S}^{j} , otherwise,} \\ \end{array} } \right.$$

where ${R}_{4}$ represents uniform random number lies in [− 2, 2], ${R}_{5}$ denotes the adaptive parameter which is determined by Eq. (8), ${R}_{6}$ represents uniform random number lies in [0, 1], $WR$ denotes the water reserves, ${Z}_{S}^{j}$ is the $j$th group leader stallion current position, ${\overline{Z} }_{S}^{j}$ is the $j$th group leader stallion next position, and ${\overline{F} }_{S}^{j}$ is its fitness value of stallion in $j$th group.

8 $$R_{5} = 1 - t \times \left( \frac{1}{T} \right)$$

#### Phase 5: Leadership transition stage to select new leader

It is quite necessary for the group to have a strong group leader so that the group may maintain discipline in a proper way and also can arrange available food sources. If in any situation, the leader of the group becomes weak, then it is essential to change the leader. The following formula is developed to model the leadership transition stage to select a new leader.

9 $$Z_{S}^{j} = \left\{ {Z_{i}^{j} { }} \right.,\;if F\left( {{ }Z_{i}^{j} } \right) < F\left( {{ }Z_{S}^{j} { }} \right),\;\forall i \in N_{j}$$

where ${Z}_{S}^{j}$ represents $j$th group leaders’ stallion current position and $F( {Z}_{S}^{j})$ is the fitness value of the leader stallion.

### Pseudo-code and flow chart of AZOA

**Figure 5:**
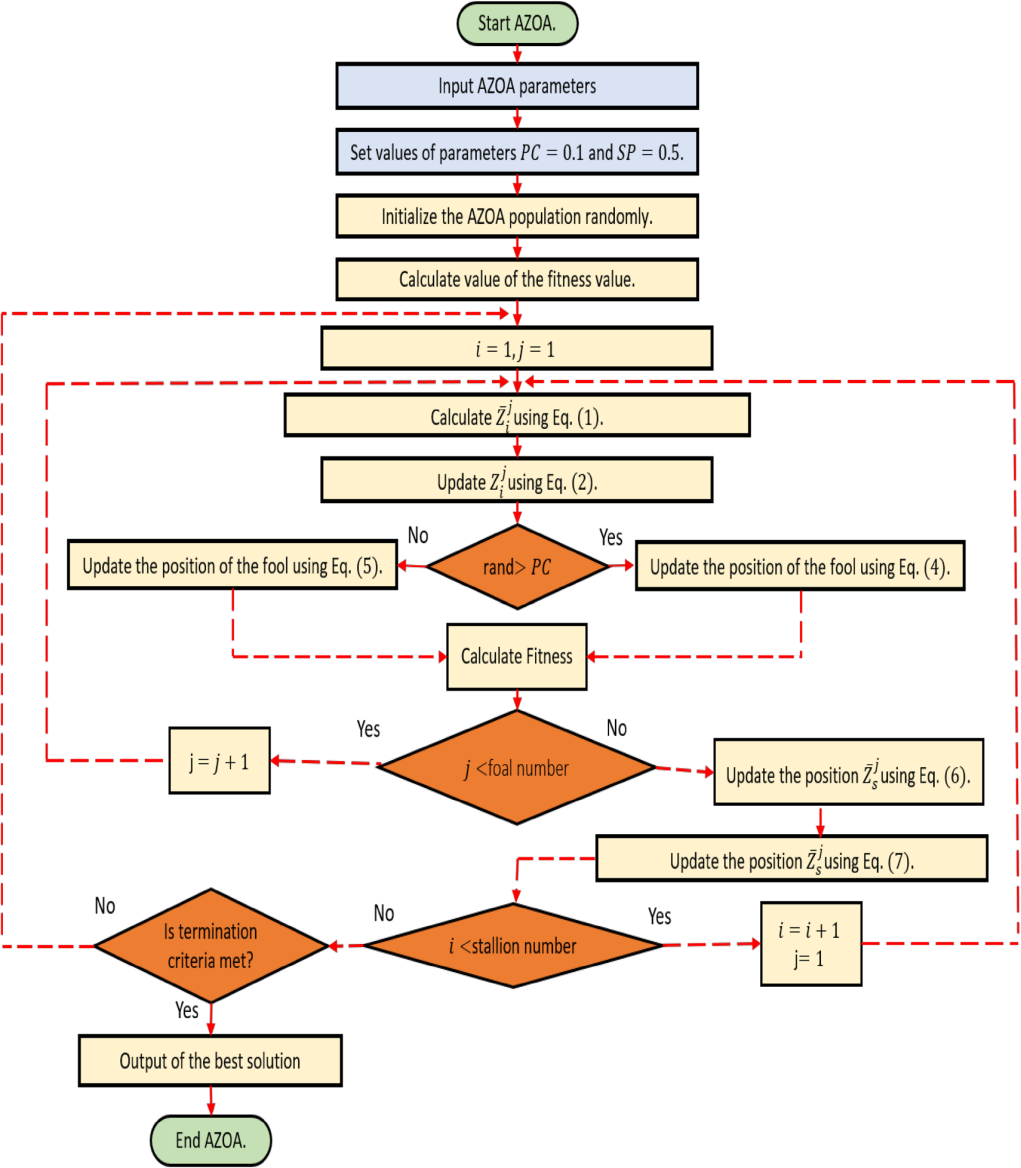
Flow chart of the proposed AZOA algorithm.


The pseudo-code and flow chart of American zebra optimization algorithm is presented in Algorithm 1 and in Fig. 5, respectively.

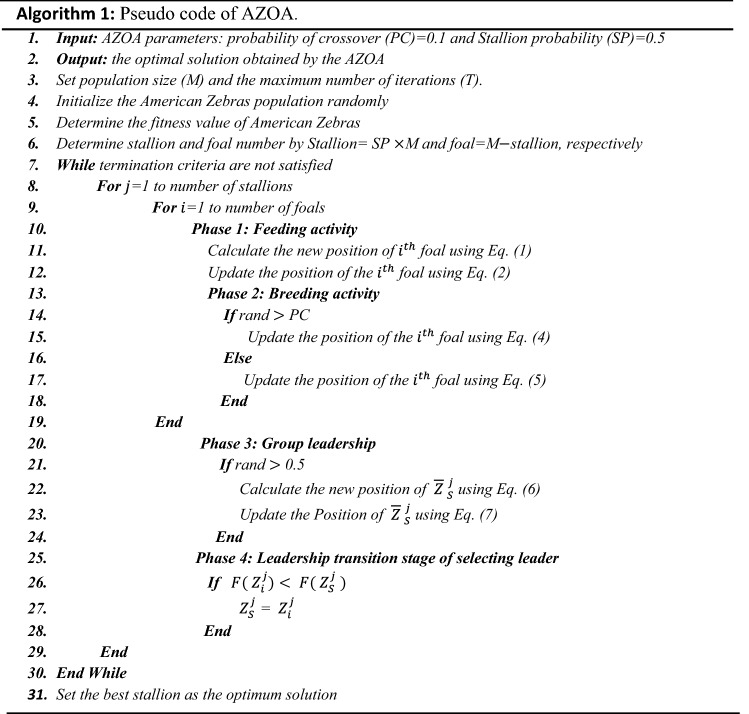


### Time complexity

The run-time complexity of AZOA depends on three procedures: initialization, evaluation of fitness value, and updating of individuals. The computational complexity of the initial process with $$M$$ individuals is O $$(M)$$, and updating the mechanism is O ($$T*M$$) + O ($$T*M*d$$), where $$T$$ represents maximum iterations and $$d$$ denotes dimension of specific problems. Hence, the total run time complexity of AZOA is O ($$M*$$($$T+Td+1$$)) which is similar to other optimizers.

## Experimental setup and result discussions

In this section, several experiments are accomplished to examine the efficiency of the newly proposed AZOA algorithm while comparing it with other meta-heuristics such as PSO, GWO, GSA, SSA, MVO, TSA, and LFD. Here, three prominent test suits, namely CEC-2005^70^, CEC-2017^71^, and CEC-2019^72^, are employed, along with three engineering problems to accomplish in the experiments. Moreover, several statistical tests like the $t$ test^73^ and the Wilcoxon rank-sum test^74^ are performed to analyse the performance of the algorithm. For the testing of benchmark functions, the number of search agents and function evaluations (NFEs) is set to 30 and 15,000, respectively. The initial controlling parameters of all algorithms are shown in Table 4. All the experiments are carried out on Windows 10, 1.70 GHz CPU, 8.00 GB RAM, and MATLAB R2021a^95^. The detailed discussions of the performance of the AZOA algorithm on each benchmark test suite are provided in the following subsections.

### Performance of AZOA on CEC-2005 benchmark test suite

The CEC-2005 is the standard test suite for researchers in computational intelligence. The ace test suite contains twenty-three benchmark functions, which may be classified into three groups: unimodal ($\mathrm{F}1{-}\mathrm{F}7$), multimodal ($\mathrm{F}8{-}\mathrm{F}13$), and fixed-dimension multimodal ($\mathrm{F}14{-}\mathrm{F}23$) functions. The list of the all-benchmark functions, along with their parameters, is presented in Tables 1, 2 and 3. Generally, all optimization algorithms have two phases: exploration and exploitation. A unimodal test function comprises a unique global optimum solution that assists in evaluating an algorithm's exploitation ability. However, the multimodal and fixed-dimension multimodal functions include multiple optimal points that help in testing the exploration capacity of the algorithm. Two assessment criteria, the mean $(avg)$ and standard deviation $(std)$, are determined using the following equations:

10 $$avg=\sum_{i=1}^{R}\frac{{x}_{i}}{R}$$

11 $$std=\sqrt{\frac{{\left({x}_{i}-avg\right)}^{2}}{R}}$$

where ${x}_{i}$ denotes the best-obtained solution from $i$th run and $R$ represents thirty independent runs.

**Table 1:** List of CEC-2005 unimodal functions.

Functions	Dim	Range	${f}_{min}$
$F1(z)$ $=\sum_{i=1}^{n}{{z}_{i}}^{2}$	30	[− 100,100]	0
$F2$(*z*) $=$ $\sum_{i=1}^{n}|z|$ + ${\prod }_{i}^{n}|{z}_{i }|$	30	[− 10,10]	0
$F3(z) =$ ${\sum }_{i=1}^{n}({\sum }_{j-1}^{i}{z}_{j })$^2^	30	[− 100,100]	0
$F4(z)=$ max_i_ {|${z}_{i}|, 1\le i\le n$}	30	[− 100,100]	0
$F5(z) = {\sum }_{i=1}^{n-1}[100({z}_{i+1}- {z}_{i}^{2})$^2^ + (${z}_{i}$ – 1)^2^]	30	[− 30,30]	0
$F6(z)$ $= {\sum }_{i=1}^{n}([{z}_{i}+0.5])$^2^	30	[− 100,100]	0
$F7(z) =$ ${\sum }_{i=1}^{n}i{z}_{i}^{4}+random[\mathrm{0,1})$	30	[− 1.28,1.28]	0

**Table 2:** List of CEC-2005 multimodal functions.

Functions	Dim	Range	${f}_{min}$
$F8(z) =$ ${\sum }_{i=1}^{n}-{z}_{i}\mathrm{sin}(\sqrt{|{z}_{i}}|)$	30	[− 500,500]	− 418.9829*5
$F9(z) = \sum _{{i = 1}}^{n} \left[ {z_{i}^{2} - 10{\text{cos}}(2\pi z_{i} ) + 10} \right]$	30	[− 5.12,5.12]	0
$F10(z)=$ − 20exp (− 0.2$\sqrt{\frac{1}{n}} {\sum }_{i=1}^{n}{z}_{i}^{2}$ ) − exp ($\frac{1}{n}{\sum }_{i=1}^{n}\mathrm{cos}(2\pi {z}_{i}))+20+e$	30	[− 32,32]	0
$F11(z)$ $=$ $\frac{1}{4000}{\sum }_{i=1}^{n}{z}_{i}^{2}-{\prod }_{i=1}^{n}\mathrm{cos}(\frac{{z}_{i}}{\sqrt{i}})+1$	30	[− 600,600]	0
$F12(z)=$ $\frac{\pi }{n}$ {10 $\mathrm{sin}\pi {y}_{1})$ +${\sum }_{i=1}^{n-1}({y}_{i}-1)$^2^ [1 + 10 ${sin}^{2}(\pi {y}_{i+1})]$ + (${y}_{n}$ − 1)^2^} + ${\sum }_{i=1}^{n}u$(${z}_{i}$,10,100,4)			
$${y}_{i}=1+\frac{{z}_{i}+1}{4}$$			
*u*($${z}_{i},a,k,m)=\left\{\begin{array}{c}{k({z}_{i}-a)}^{m} {z}_{i}>a\\ 0 -a<{z}_{i}<a \\ {k({-z}_{i}-a)}^{m} {z}_{i} <-a\end{array}\right.$$	30	[− 50,50]	0
$F13(z)=$ 0.1{sin^2^(3 $\pi {z}_{1})+{\sum }_{i=1}^{n}{({z}_{i}-1)}^{2}[1+{sin}^{2}$ (3 $\pi {z}_{i}+1$)] + ${({z}_{n}-1)}^{2}[1+{sin}^{2}\left(2\pi {z}_{n}\right)]\}$ + ${\sum }_{i=1}^{n}u({z}_{i},\mathrm{5,100,4})$	30	[− 50,50]	0

**Table 3:** List of CEC-2005 Fixed dimension multimodal functions.

Functions	Dim	Range	${f}_{min}$
$F14(z) =$($\frac{1}{500}+{\sum }_{j=1}^{25}\frac{1}{j+{\sum }_{i=1}^{2}{({z}_{i}-{a}_{ij})}^{6}})$^−1^	2	[− 65,65]	1
$F15(z) =$ ${\sum }_{i=1}^{11}[{a}_{i}-\frac{{z}_{1}\left({b}_{i}^{2}+{b}_{i}{z}_{2}\right)}{{b}_{i}^{2}+{b}_{i}{z}_{3}+{z}_{4}}]$^2^	4	[− 5,5]	0.00030
$F16(z) =$ 4 ${z}_{1}^{2}-$ 2.1 ${z}_{1}^{4}$ +$\frac{1}{3}{z}_{1}^{6}+ {z}_{1}{z}_{2}-4{z}_{2}^{2}+4{z}_{2}^{4}$	2	[− 5,5]	− 1.0316
$F17(z) =$(${z}_{2}-\frac{5.1}{{4\pi }^{2}}{z}_{1}^{2}+\frac{5}{\pi }{z}_{1}-6)$^2^ + 10(1 − $\frac{1}{8\pi }$)$\mathrm{cos}{z}_{1}+10$	2	[− 5,5]	0.398
$$F18(z) =$$[1 + ($${z}_{1}+{z}_{2}+1)$$^2^ (19–14 $${z}_{1}{+3z}_{1}^{2}-14{z}_{2}+6{z}_{1}{z}_{2}+3{z}_{2}^{2})] \times$$ [30 + (2 $${z}_{1}-3{z}_{2})$$^2^ $$\times$$ (18–32 $${z}_{1}+12{z}_{1}^{2}+48{z}_{2}-36{z}_{1}{z}_{2}+27{z}_{2}^{2})]$$	2	[− 2,2]	3
$F19(z) =- {\sum }_{i=1}^{4}{c}_{i}\mathrm{exp}(-{\sum }_{j=1}^{3}{a}_{ij}({z}_{j}-{p}_{ij})$^2^)	3	[1,3]	− 3.86
$F20(z) =- {\sum }_{i=1}^{4}{c}_{i}exp$ ($-{\sum }_{j=1}^{6}{a}_{ij}({{z}_{j}-{p}_{ij})}^{2})$	6	[0,1]	− 3.32
$F21(z) =- {\sum }_{i=1}^{5}[(Z-{a}_{i}){(Z-{a}_{i})}^{T}+{c}_{i}]$^−1^	4	[0,10]	− 10.1532
$F22(z) =- {\sum }_{i=1}^{7}[(Z-{a}_{i}){(Z-{a}_{i})}^{T}+{c}_{i}]$^−1^	4	[0,10]	− 10.4028
$F23(z) =- {\sum }_{i=1}^{10}[(Z-{a}_{i}){(Z-{a}_{i})}^{T}+{c}_{i}]$^−1^	4	[0,10]	− 10.5363

**Figure 6:**
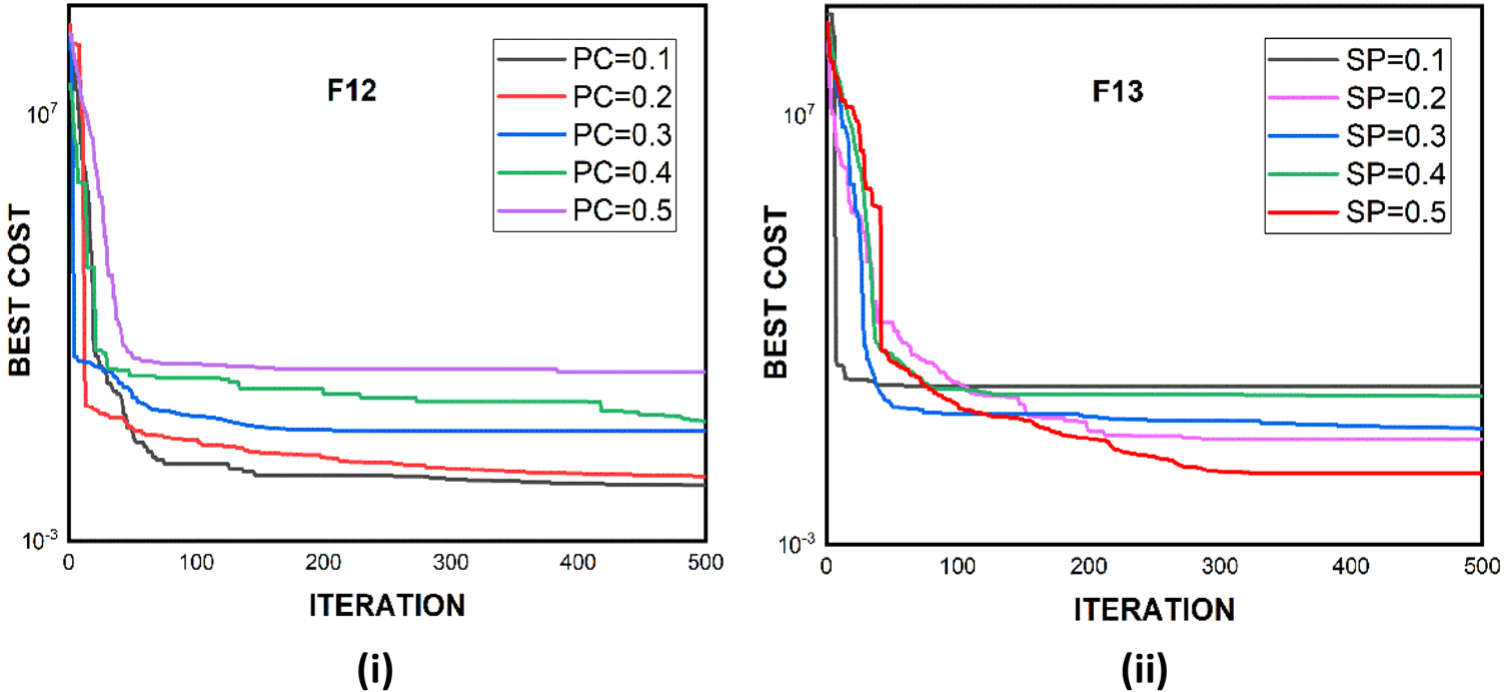
Sensitivity analysis of proposed AZOA algorithm for parameters *PC* and *SP*.

The statistical parameters $avg$ and $std$ quantify the performance of an algorithm. The lesser the value of $avg$, the better the algorithm's ability to obtain a solution close to the global optimal. Even if the two algorithms have the same $avg$ value, their performance in obtaining the global optimal may vary in each generation. As a result, $std$ is employed to establish a more accurate comparison. The $std$ should have a low value to have less variation in the outcomes. The statistical outcomes in terms of average and standard deviation of AZOA along with their compared algorithm are reported in Table 5. Table 5 demonstrate that AZOA performed better in all the unimodal functions except $\mathrm{F}6$ than other compared algorithms in exploitation abilities. The results of multimodal functions indicate that AZOA is able to outperform other meta-heuristics in terms of exploration ability. On the other hand, GSA and PSO performed admirably for functions $\mathrm{F}8$ and $\mathrm{F}13$, respectively. The outcomes of fixed-dimensional and multimodal functions illustrate that AZOA performs more effectively in optimizing $\mathrm{F}14{-}\mathrm{F}16$ and $\mathrm{F}20{-}\mathrm{F}23$. However, these results are further needed to be tested for checking the statistical significance between the algorithm. Hence, the imperative statistical tests, such as the $t$ test and the Wilcoxon rank-sum test at $\alpha$ = 0.05% significant level, are required to indicate a significant enhancement of the proposed algorithm. Let ${avg}_{1}$, ${avg}_{2}$ and ${std}_{1}$, ${std}_{2}$ be the mean and standard deviation for the two algorithms, respectively. The outcomes of the $t$ test at $\alpha$ = 0.05% for each function are presented in Table 5, which are calculated by Eq. (12). The sensitivity analysis of proposed AZOA algorithm is carried out in Fig. 6.

12 $$t=\frac{{ avg}_{1}-{ avg}_{2}}{\sqrt{\frac{{{std}_{1}}^{2}+{{std}_{2}}^{2}}{R}}}$$

**Figure 7:**
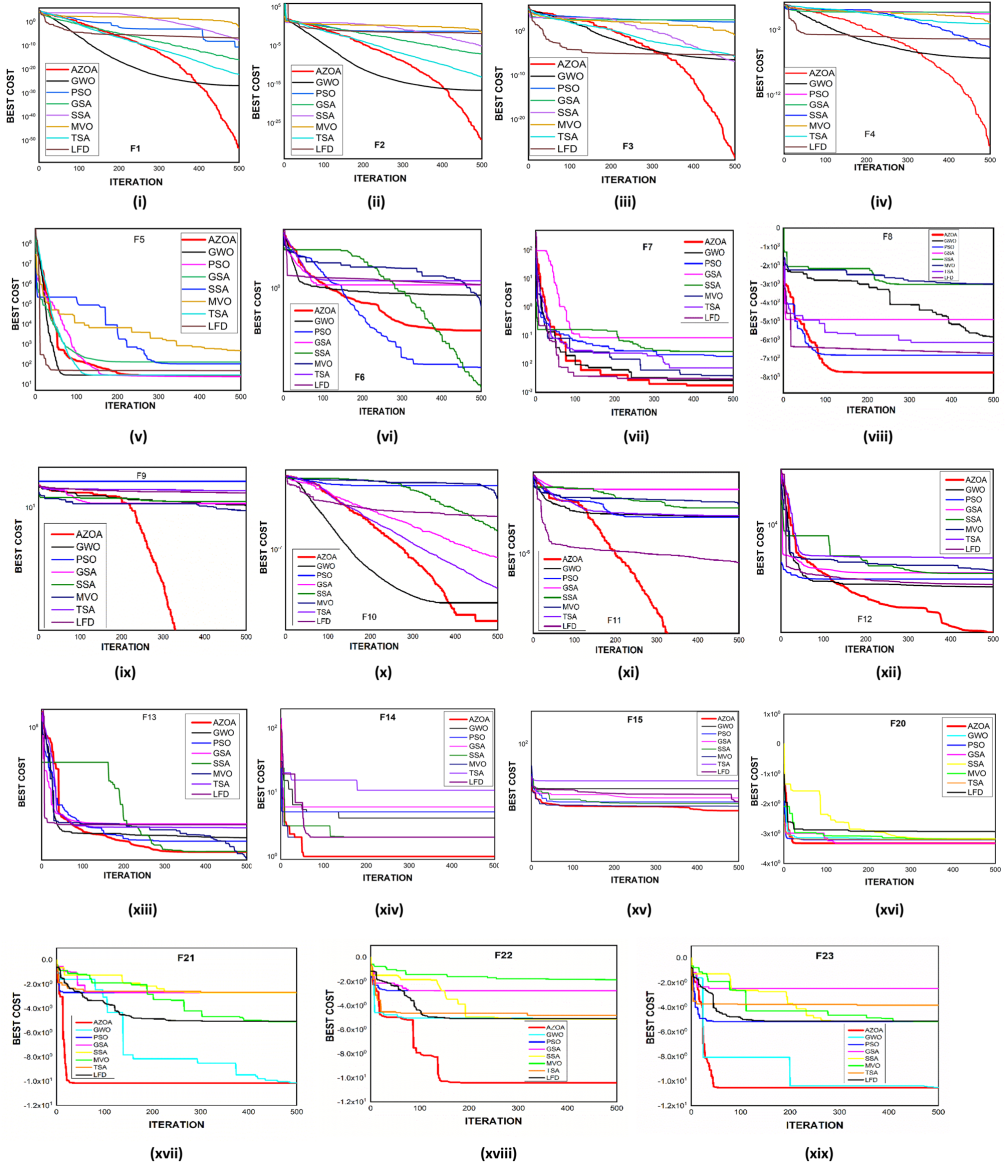
Convergence graph of AZOA and other metaheuristics in solving CEC-2005 benchmark functions.

**Figure 8:**
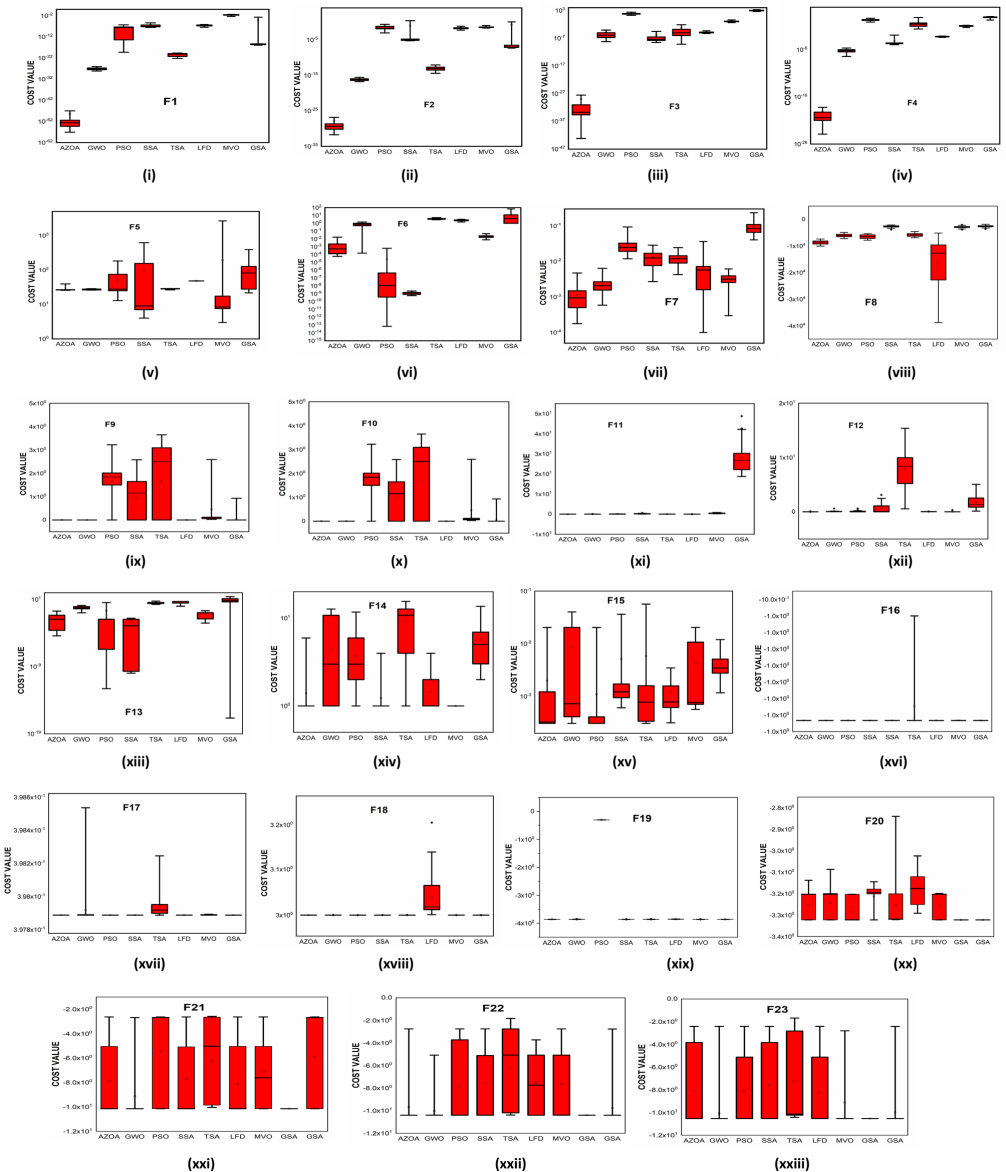
Box plots of AZOA and other metaheuristics in solving CEC-2005 benchmark.

If the corresponding $t$-value is boldfaced, AZOA performs significantly better in comparison to other algorithms. In a tie situation, the results are displayed in bold italics letter. Moreover, the last rows of each table, labelled as $w/t/l$, indicate AZOA win, tie, and loss counts over the certain algorithm in terms of $t$-values. Clearly, from the $t$-values, it is observed that the performance of AZOA is statistically significant difference in most cases. The outcomes of the Wilcoxon rank-sum test of AZOA at $\alpha$ = 0.05% significant level is presented in Table 6. Here, $\mathrm{H}=$ $1$ and $\mathrm{H }= 0$ indicate acceptance and rejection, respectively, whereas $Na$ indicates the equivalent optimum values of the two algorithms. From Table 6 it is observed that the most of the $p$ values are smaller than 0.05, which clearly shows that the AZOA algorithm performs superiorly in comparison to other meta-heuristics. After the statistical tests, it is necessary to check the convergence graph of the algorithms. The main objective behind the convergence analysis is to understand the behavior and graphical representation of the proposed AZOA algorithm. Hence, the convergence curves of the algorithms for some test functions are presented in Fig. 7. As seen from the convergence curves, the proposed algorithm in functions $\mathrm{F}1{-}\mathrm{F}4$ follows a certain smooth pattern, which gives more emphasis to the exploitation. In functions $\mathrm{F}8$, $\mathrm{F}9$, $\mathrm{F}11$, and $\mathrm{F}22$, the proposed algorithm follows a different pattern which has many optimal points. It focuses more on the exploration phases that are accomplished in the early phases of the algorithm. However, in the last phases of the algorithm, which is generally the exploitation phase, the AZOA has performed stepwise for functions $\mathrm{F}10$ and $\mathrm{F}12$. In functions $\mathrm{F}14$, $\mathrm{F}15$, $\mathrm{F}20$, and $\mathrm{F}23$, the proposed algorithm accomplishes comparable convergence. As a result, the AZOA exhibits a superior convergence pattern in almost all functions. In order to further analyse and graphically compare the performance of the optimization techniques, the whisker-box plot^75^ for each metaheuristic and objective function is displayed in Fig. 8. The central box represents the value between the first, and third quartiles and the black line denotes the median. It can be observed from Fig. 8 that AZOA performs better than the other state-of-the-art metaheuristics. It also demonstrates that AZOA has better performance and superior convergence ability in the component exploitation and exploration processes. In summary, depending on the outcomes and analyses of the algorithms' performance on CEC-2005, the proposed AZOA algorithm is capable of obtaining superior solutions for most of the test functions and produces statically significantly better outcomes than other metaheuristics.

### Sensitivity analysis

The proposed algorithm, namely AZOA, employs two parameters: parameter PC (probability of crossover) and parameter SP (stallion probability or number of groups). The sensitivity analysis of these parameters has been explained by changing their values while keeping the other parameters constant, as shown in Table 4.

**Table 4:** Values for the controlling parameters of compared algorithms.

Algorithm	Parameter	Value
PSO	$\mathrm{W}$	$1$
	${W}_{p}$	$0.99$
	${C}_{1}$	$1.5$
	${C}_{2}$	$2.0$
GSA	${G}_{0}$	$100$
	$\alpha$	$20$
	${R}_{power}$	$1$
GWO	Convergence parameter (a)	$a$ $=$ $2 - 2 \times (FEs/MaxFEs)$
MVO	${WEP}_{max}$	$1$
	${WEP}_{min}$	$0.2$
SSA	${C}_{1}$	$2 \times exp((4 \times FEs/MaxFEs)2 )$
TSA	${P}_{min}$	$1$
	${P}_{max}$	$4$
	${C}_{1}$, ${C}_{2}$,${C}_{3}$	Random numbers stand in the interval $[\mathrm{0,1}].$
LFD	Search agent	$35$
	Threshold	$2$
	CSV	$0.5$
	$\upbeta$	$1.5$
	${\alpha }_{1}$	$10$
	${\alpha }_{2}$	$0.00005$
	${\alpha }_{3}$	$0.005$
	${\partial }_{1}$	$0.9$
	${\partial }_{2}$	$0.1$
FFT	$K$ value	$2$
	$\mathrm{\alpha }$	$0.6$
	$\upbeta$	$0.4$
	$\mathrm{W}$	$1$
	$\mathrm{Q}$	$0.7$
MGO		Free parameter
AVOA	${L}_{1}$	$0.8$
	${L}_{2}$	$0.2$
	$\mathrm{W}$	$2.5$
	${P}_{1}$	$0.6$
	${P}_{2}$	$0.4$
	${P}_{3}$	$0.6$
GTO	$\upbeta$	$3$
	$\mathrm{W}$	$0.8$
	$P$	$0.03$
AZOA	Probability crossover (PC)	$0.1$
	Stallion probability (SP)	$0.5$
	Crossover	$mean$

#### Variation in the parameter PC

To examine the impact of parameter PC, the AZOA algorithm was performed for various values of PC while keeping the other parameters constant. The different values of PC tested in experimentation are 0.1, 0.2, 0.3, 0.4, and 0.5. The variation of PC on standard benchmark functions is depicted in Fig. 6(i). The results reveal that when the value of PC is set to 0.1, AZOA produces better optimal outcomes (Tables 5, 6).

**Table 5:** Statistical outcomes of AZOA and other algorithms in solving CEC-2005 benchmark functions.

Function	AZOA	GWO	PSO	GSA	SSA	MVO	TSA	
	$avg (std)$	$avg (std)$ $t$-$values$	$avg (std)$ $t$-$values$	$avg (std)$ $t$-$values$	$avg (std)$ $t$-$values$	$avg (std)$ $t$-$values$	$avg (std)$ $t$-$values$	$avg (std)$ $t$-$values$
$\mathrm{F}1$	1.160E−10 (3.340E−108)	1.570E−27 (2.350E−27) **(**−**3.660E+00)**	7.570E−06 (3.170E−05) **(**−**3.690E+00)**	2.530E−16 (9.670E−17) **(**−**1.43E+01)**	2.160E−07 (3.580E−09) **(**−**3.310E+00)**	1.230E+00 (3.720E−01) **(**−**1.830E+01)**	1.190E−21 (3.810E−21) **(**−**1.720E+21)**	3.180E−07 (9.910E−08) **(**−**1.760E+01)**
$\mathrm{F}2$	2.310E−57 (6.630E−57)	1.150E−16 (1.170E−16) **(**−**5.380E+00)**	1.760E−02 (6.420E−02) **(**−**5.08E+00)**	1.940E−01 (1.500E−09) **(**−**7.09E+08)**	1.860E+00 (1.35E+00) **(**−**7.570E+00)**	1.270E+01 (3.390E+01) **(**−**2.050E+00)**	1.200E−13 (1.670E−13) **(**−**3.930E+00)**	3.380E−04 (5.920E−05) **(**−**3.130E+01)**
$\mathrm{F}3$	4.560E−74 (2.460E−73)	3.650E−06 (4.550E−06) **(**−**4.390E+00)**	1.510E+02 (8.410E+01) **(**−**1.740E+01)**	6.800E−11 (7.40E−11) **(**−**5.03E+00)**	1.580E+03 (9.70E+02) **(−8.940E+00)**	2.230E+02 (8.270E+01) **(−1.48E+01)**	7.360E−04 (2.200E−03) **(−1.830E+00)**	1.500E−06 (5.260E−07) **(−1.570E+01)**
$\mathrm{F}4$	4.690E−48 (1.350E−47)	5.4800E−07 (6.29E−07) **(−4.770E+00)**	2.600E+00 (8.140E−01) **(−1.880E+01)**	1.740E+00 (7.35E+00) **(*−1.300E*+*00*)**	1.070E+01 (3.030E+00) **(−1.930E+01)**	2.070E+00 (7.280E−01) **(−1.56E+01)**	3.420E−01 (4.550E−01) **(−4.110E+00)**	3.550E−04 (5.780E−05) **(−3.360E+01)**
$\mathrm{F}5$	2.660E+01 (6.140E−01)	2.700E+01 (7.630E−01) **(−3.730E+00)**	4.800E+001 (3.40E+01) **(−6.41E+00)**	6.750E+01 (6.220E+01) **(−3.620E+00)**	6.430E+002 (2.180E+03) **(*−1.550E*+*00*)**	3.770E+02 (6.190E+02) **(−3.100E+00)**	2.850E+01 (4.970E−01) **(−1.760E+01)**	2.800E+001 (1.430E−01) **(−1.950E+01)**
$\mathrm{F}6$	2.690E−04 (9.923E−04)	7.760E−01 (3.960E−01) **(−1.07E+001)**	1.880E−06 (2.900E−06) (2.200E+00)	2.500E−16 (1.7400E−16) (7.430E+00)	2.730E−07 (7.930E−07) (7.410E+00)	1.300E+00 (3.910E−01) **(−1.830E+01)**	3.700E+00 (5.950E−01) **(−3.410E+01)**	1.870E+00 (2.360E−01) **(−4.360E+01)**
$\mathrm{F}7$	−7.340E+03(8.628E+02)	1.800E−03 (1.10E−03) **(−3.570E+00)**	2.380E−02 (8.20E−03) **(−1.480E+01)**	8.940E−02 (4.300E−02) **(−1.120E+01)**	1.650E−01 (6.190E−02) **(−1.450E+01)**	3.680E−02 (1.560E−02) **(−1.260E+01)**	1.150E−02 (4.200E−03) **(−1.360E+01)**	1.060E+00 (6.340E−01) **(−9.190E+00)**
$\mathrm{F}8$	0.000E+00 (0)	−5.800E+03 (8.360E+02) **(−1.930E+01)**	−6160E+03 (8.090E+02) (3.360E+03)	−2.820E+03 (4.930E+02) **(−4.770E+01)**	−7.290E+03 (7.680E+02) **(−1.190E+01)**	−7.610E+03 (5.540E+02) **(−1.210E+01)**	−5.900E+03 (6.780E+02) **(−2.100E+01)**	−4.070E+03 (3.410E+02) **(−4.380E+01)**
$\mathrm{F}9$	8.880E−16 (1.002E−31)	2.910E+00 (4.210E+000) **(−3.770E+000)**	4.810E+01 (2.200E+001) **(*−1.260E*+*001*)**	2.600E+001 (7.470E+00) **(−1.900E+01)**	5.320E+01 (1.840E+01) **(−5.480E+00)**	1.120E+02 (2.510E+01) **(−2.460E+01)**	1.870E+02 (5.110E+01) **(−2.010E+01)**	4.490E−06 (2.860E−06) **(−8.590E+00)**
$\mathrm{F}10$	0.000E+00 (0)	1.000E−13 (1.630E−14) **(−3.330E+01)**	1.140E+00 (8.140E−01) **(−7.670E+00)**	6.210E−02 (2.360E−01) **(*−1.440E*+*00*)**	2.3100E+00 (7.20E−01) **(−1.750E+01)**	2.330E+00 (3.280E+00) **(−3.900E+00)**	1.000E+00 (1.550E+00) **(−3.510E+00)**	1.330E−04 (1.950E−05) **(−3.750E+01)**
$\mathrm{F}11$	1.190E−02 (1.719E−02)	3.120E−02 (5.100E−03) **(−3.350E+01)**	2.810E−02 (4.190E−02) **(−3.670E+00)**	2.700E+01 (5.0400E+00) **(−3.0100E+01)**	1.820E−02 (1.24E−02) **(−7.970E+00)**	8.400E−01 (9.170E−02) **(−5.070E+01)**	8.80E−03 (8.30E−03) **(−5.810E+00)**	8.220E−07 (2.360E−07) **(−1.910E+01)**
$\mathrm{F}12$	1.390E+00 (5.848E−01)	4.510E−02 (2.610E−02) **(−9.460E+00)**	5.890E−02 (1.110E−01) **(−2.910E+00)**	1.800E+00 (9.510E−01) **(−1.040E+01)**	6.620E+00 (3.660E+00) **(−9.900E+00)**	2.28900E+00 (1.220E+00) **(−1.020E+01)**	8.230E+00 (4.690E+00) **(−9.610E+00)**	7.810E−01 (1.340E−01) **(−3.190E+01)**
$\mathrm{F}13$	9.980E−01 (3.387E−16)	6.300E−01 (2.100E−01) **(−1.580E+001)**	1.800E−02 (4.630E−02) **(*7.470E−002*)**	8.900E+00 (7.130E+00) **(−6.830E+00)**	1.580E+01 (1.480E+01) **(−5.820E+00)**	1.800E−01 (1.090E−01) **(−7.880E+00)**	2.870E+00 (5.010E−01) **(−3.120E+01)**	2.950E+00 (2.350E−02) **(−4.980E+02)**
$\mathrm{F}14$	3.510E−04 (5.314E−04)	4.040E+00 (4.25E+00) **(−3.920E+00)**	3.630E+00 (2.560E+00) **(−5.620E+00)**	5.860E+00 (3.83E+00) **(−6.950E+00)**	1.100E+00 (3.140E−01) **(*−1.730E*+*00*)**	9.980E−01 (0) **(*0.000E*+*00*)**	8.780E+00 (5.79733) **(−7.350E+00)**	1.300E+00 (4.670E−01) **(−3.540E+00)**
$\mathrm{F}15$	−1.030E+00 (1.649E−08)	3.370E−04 (6.250E−04) **(*−2.080E−01*)**	5.770E−04 (2.520E−03) **(−4.740E+00)**	3.670E−03 (1.650E−03) **(−1.110E+01)**	1.220E−03 (6.120E−03) **(*−8.120E−01*)**	2.680E−03 (6.210E−03) **(−2.090E+00)**	1.070E−02 (1.060E−02) **(−5.370E+00)**	9.830E−04 (4.420E−04) **(−7.500E+00)**
$\mathrm{F}16$	3.980E−01 (1.129E−16)	−1.030E+000 (−1.030E+00) **(*1.130E−04*)**	−1.03163 (6.250E−16) **(*1.590E−04*)**	−1.030E+00 (4.880E−16) **(*1.590E−04*)**	−1.030E+000 (0) **(*0.000E*+*000)***	−1.030E+00(0) **(*0.000E*+*000)***	−1.030E+00 (5.700E−05) **(*1.060E−04*)**	−1.030E+000 (0) **(*0.000E*+*000)***
$\mathrm{F}17$	2.690E−04 (9.923E−04)	3.980E−01 (3.980E−01) **(*9.730E−06*)**	3.980E−01 (0) **(*4.130E−05*)**	3.980E−01 (0) **(*4.130E−05*)**	3.980E−001 (0) (3.870E+00)	3.980E−001 (0) ***(0.000E*+*00)***	3.980E−01 (5.300E−05) ***(−5.500E−004)***	3.980E−001 (0) ***(0.000E*+*000*)**
$\mathrm{F}18$	3.000E+00 (1.322E−14)	3.000E+000 (3) ***(−5.110E−05)***	3 (1.330E−15) **(*0.000E*+*00*)**	3 (4.170E−15) **(*0.000E*+*00*)**	3 (0) **(*0.000E*+*00)***	3(0) **(*0.000E*+*00*)**	3.000E+00 (5.980E−04) **(−2.290E+00)**	3.070E+00 (7.450E−02) **(−5.380E+00)**
$\mathrm{F}19$	−3.860E+00 (3.498E−15)	−3.860E+00 (−3.860E+00) ***(−2.410E−04*)**	6.050E−02 (2.580E−15) **(−4.250E+10)**	−3.8600E+00 (2.290E−15) **(−4.780E+10)**	−3.860E+00 (0) **(*0.000E*+*00)***	−3.860E+00 (0) ***(0.000E*+*000)***	−3.860E+00 (3.000E−03) **(−4.980E+00)**	−3.860E+00 (2.400E−03) **(−1.550E+001)**
$\mathrm{F}20$	−3.270E+00 (5.992E−02)	−3.210E+00 (3.250E+00) ***(−1.950E−01)***	−3.270E+00 (6.05E−02) **(−4.520E+00)**	2.31E−02 (2.31E−02) **(−4.870E+02)**	−3.220E+00 (5.640E−02) **(−8.950E+00)**	−3.270E+00 (6.22E−02) **(−3.830E+00)**	−3.330E+00 (1.080E−01) **(*1.910E−01*)**	−3.200E+00 (7.670E−02) **(−8.190E+00)**
$\mathrm{F}21$	−1.020E+01 (1.806E−15)	−1.020E+01 (−9.130E+00) ***(−1.050E−03)***	−6.870E+00 (3.020E+00) **(−4.770E+00)**	−5.960E+00 (3.740E+00) **(−5.260E+00)**	2.680E+00 (3.300E+00) **(−1.020E+001)**	−7.370E+00 (3.020E+00) **(−4.030E+00)**	−5.590E+00 (3.050E+00) **(−6.580E+00)**	−6.610E+00 (3.380E+00) **(−4.770E+00)**
$\mathrm{F}22$	−1.040E+01 (0)	−1.040E+01 (−8.450E+00) ***(−8.520E−04)***	−8.460E+00 (3.090E+00) **(−2.440E+00)**	−9.680E+00 (2.010E+00) ***(−1.070E*+*00*)**	−9.350E+00 (2.220E+00) ***(−1.520E*+*00*)**	−8.580E+00 (3.000E+00) **(−2.310E+00)**	−5.500E+00 (3.230E+00) **(−6.010E+000)**	−1.040E+001 (0) ***(0.000E*+*000)***
$\mathrm{F}23$	−1.050E+01 (9.033E−15)	−1.050E+01 (−8.56E+00) ***(−1.250E−03)***	−9.950E+00 (1.78E+00) ***(−8.190E−01*)**	−1.050E+001 (2.60E−15) **(*0.000E*+*000*)**	−9.720E+00 (2.570E+00) ***(−1.030E*+*00)***	−1.000E+01 (1.710E+00) ***(−7.660E−01)***	−7.05E+00 (3.8200E+00) **(−3.700E+00)**	−1.050E+01 (0) **(*0.000E*+*00)***
$w/t/l$		14/9/0	15/6/2	14/8/1	13/8/2	17/6/0	20/3/0	19/4/0

Significant values are in bold italic.

**Table 6:** $p$ values by Wilcoxon Rank Sum test with five percent significance level for $\mathrm{F}1{-}\mathrm{F}23$.

Function	GWO versus AZOA		PSO versus AZOA		TSA versus AZOA		SSA versus AZOA		MVO versus AZOA		LFD versus AZOA		GSA versus AZOA	
	*p* values	$H$	*p* values	$H$	*p* values	$H$	*p* values	$H$	*p* values	*H*	*p* values	$H$	*p* values	$H$
$\mathrm{F}1$	$1.771E{-}06$	$1$	$1.779E{-}06$	$1$	$1.779E{-}06$	$1$	$1.779E{-}06$	$1$	$1.779E{-}06$	$1$	$1.779E{-}06$	$1$	$1.779E{-}06$	$1$
$\mathrm{F}2$	$1.779E{-}06$	$1$	$1.779E{-}06$	$1$	$1.779E{-}06$	$1$	$1.779E{-}06$	$1$	$1.779E{-}06$	$1$	$1.779E{-}06$	$1$	$1.779E{-}06$	$1$
$\mathrm{F}3$	$1.770E{-}06$	$1$	$1.779E{-}06$	$1$	$1.779E{-}06$	$1$	$1.779E{-}06$	$1$	$1.779E{-}06$	$1$	$1.779E{-}06$	$1$	$1.779E{-}06$	$1$
$\mathrm{F}4$	$1.770E{-}06$	$1$	$1.778E{-}06$	$1$	$1.779E{-}06$	$1$	$1.770E{-}06$	$1$	$1.779E{-}06$	$1$	$1.779E{-}06$	$1$	$1.779E{-}06$	$1$
$\mathrm{F}5$	$6.340E{-}02$	$0$	$3.730E{-}02$	$1$	$2.270E{-}04$	$1$	$2.080E{-}01$	$0$	$3.740E{-}01$	$0$	$1.779E{-}06$	$1$	$4.960E{-}05$	$1$
$\mathrm{F}6$	$1.770E{-}06$	$1$	$1.779E{-}06$	$1$	$1.779E{-}06$	$1$	$1.779E{-}06$	$1$	$1.779E{-}06$	$1$	$1.779E{-}06$	$1$	$4.840E{-}06$	$1$
$\mathrm{F}7$	$1.510E{-}04$	$1$	$1.779E{-}06$	$1$	$1.90E{-}06$	$1$	$1.779E{-}06$	$1$	$1.150E{-}05$	$1$	$5.920E{-}05$	$1$	$1.779E{-}06$	$1$
$\mathrm{F}8$	$1.779E{-}06$	$1$	$1.779E{-}06$	$1$	$1.779E{-}06$	$1$	$1.770E{-}06$	$1$	$1.779E{-}06$	$1$	$2.090E{-}04$	$1$	$1.779E{-}06$	$1$
$\mathrm{F}9$	$1.776E{-}06$	$1$	$1.771E{-}06$	$1$	$1.779E{-}06$	$1$	$1.779E{-}06$	$1$	$1.779E{-}06$	$1$	$1.770E{-}06$	$1$	$1.760E{-}06$	$1$
$\mathrm{F}10$	$1.746E{-}06$	$1$	$1.707E{-}06$	$1$	$1.779E{-}06$	$1$	$1.760E{-}06$	$1$	$1.770E{-}06$	$1$	$1.779E{-}06$	$1$	$1.779E{-}06$	$1$
$\mathrm{F}11$	$3.910E{-}03$	$1$	$1.772E{-}06$	$1$	$4.880E{-}04$	$1$	$1.779E{-}06$	$1$	$1.779E{-}06$	$1$	$1.779E{-}06$	$1$	$1.779E{-}06$	$1$
$\mathrm{F}12$	$2.018E{-}05$	$1$	$4.310E{-}01$	$0$	$1.779E{-}06$	$1$	$1.500E{-}02$	$1$	$2.270E{-}04$	$1$	$3.660E{-}04$	$1$	$1.779E{-}06$	$1$
$\mathrm{F}13$	$1.779E{-}06$	$1$	$6.040E{-}01$	$0$	$1.779E{-}06$	$1$	$2.290E{-}03$	$1$	$6.940E{-}03$	$1$	$1.700E{-}06$	$1$	$1.975E{-}06$	$1$
$\mathrm{F}14$	$7.540E{-}04$	$1$	$3.260E{-}04$	$1$	$2.530E{-}06$	$1$	$6.250E{-}01$	$0$	$3.130E{-}02$	$1$	$3.300E{-}01$	$0$	$5.340E{-}06$	$1$
$\mathrm{F}15$	$1.060E{-}02$	$1$	$1.630E{-}02$	$1$	$1.730E{-}01$	$0$	$7.380E{-}03$	$1$	$2.340E{-}02$	$1$	$4.330E{-}02$	$1$	$3.960E{-}04$	$1$
$\mathrm{F}16$	$8.426E{-}06$	$1$	$4.466E{-}08$	$1$	$1.779E{-}06$	$1$	$NA$	$0$	$1.779E{-}06$	$1$	$NA$	$0$	$A$	$0$
$\mathrm{F}17$	$1.779E{-}06$	$1$	$NA$	$0$	$1.779E{-}06$	$1$	$NA$	$0$	$1.779E{-}06$	$1$	$NA$	$0$	$NA$	$0$
$\mathrm{F}18$	$1.779E{-}06$	$1$	$NA$	$0$	$1.779E{-}06$	$1$	$NA$	$0$	$1.779E{-}06$	$1$	$1.779E{-}06$	$1$	$NA$	$0$
$\mathrm{F}19$	$1.779E{-}06$	$1$	$4.466E{-}08$	$1$	$1.779E{-}06$	$1$	$3.080E{-}01$	$0$	$1.779E{-}06$	$1$	$1.779E{-}06$	$1$	$NA$	$0$
$\mathrm{F}20$	$2.310E{-}01$	$0$	$2.660E{-}02$	$1$	$4.440E{-}01$	$0$	$1.980E{-}03$	$1$	$2.080E{-}01$	$0$	$3.010E{-}03$	$1$	$8.459E{-}05$	$1$
$\mathrm{F}21$	$8.980E{-}01$	$1$	$1.150E{-}02$	$1$	$4.760E{-}03$	$1$	$8.870E{-}01$	$0$	$1.600E{-}01$	$0$	$9.220E{-}01$	$0$	$6.610E{-}02$	$0$
$\mathrm{F}22$	$2.630E{-}03$	$1$	$1.290E{-}01$	$0$	$1.390E{-}04$	$1$	$1.660E{-}02$	$0$	$3.660E{-}04$	$1$	$1.410E{-}02$	$1$	$6.880E{-}01$	$0$
$\mathrm{F}23$	$9.790E{-}01$	$0$	$9.700E{-}01$	$0$	$3.010E{-}03$	$1$	$5.920E{-}01$	$0$	$9.630E{-}01$	$0$	$8.960E{-}01$	$0$	$4.200E{-}02$	$1$

#### Variation in the parameter SP

To examine the impact of the parameter SP, the AZOA algorithm was performed for various values of SP while keeping the other parameters constant. The different values of PC tested in experimentation are 0.1, 0.2, 0.3, 0.4, and 0.5. The variation of SP on standard benchmark functions is depicted in Fig. 6(ii). The results reveal that when the value of SP is set to 0.1, AZOA produces better optimal outcomes.

### Performance of AZOA on the CEC-2017 benchmark test suite

**Figure 9:**
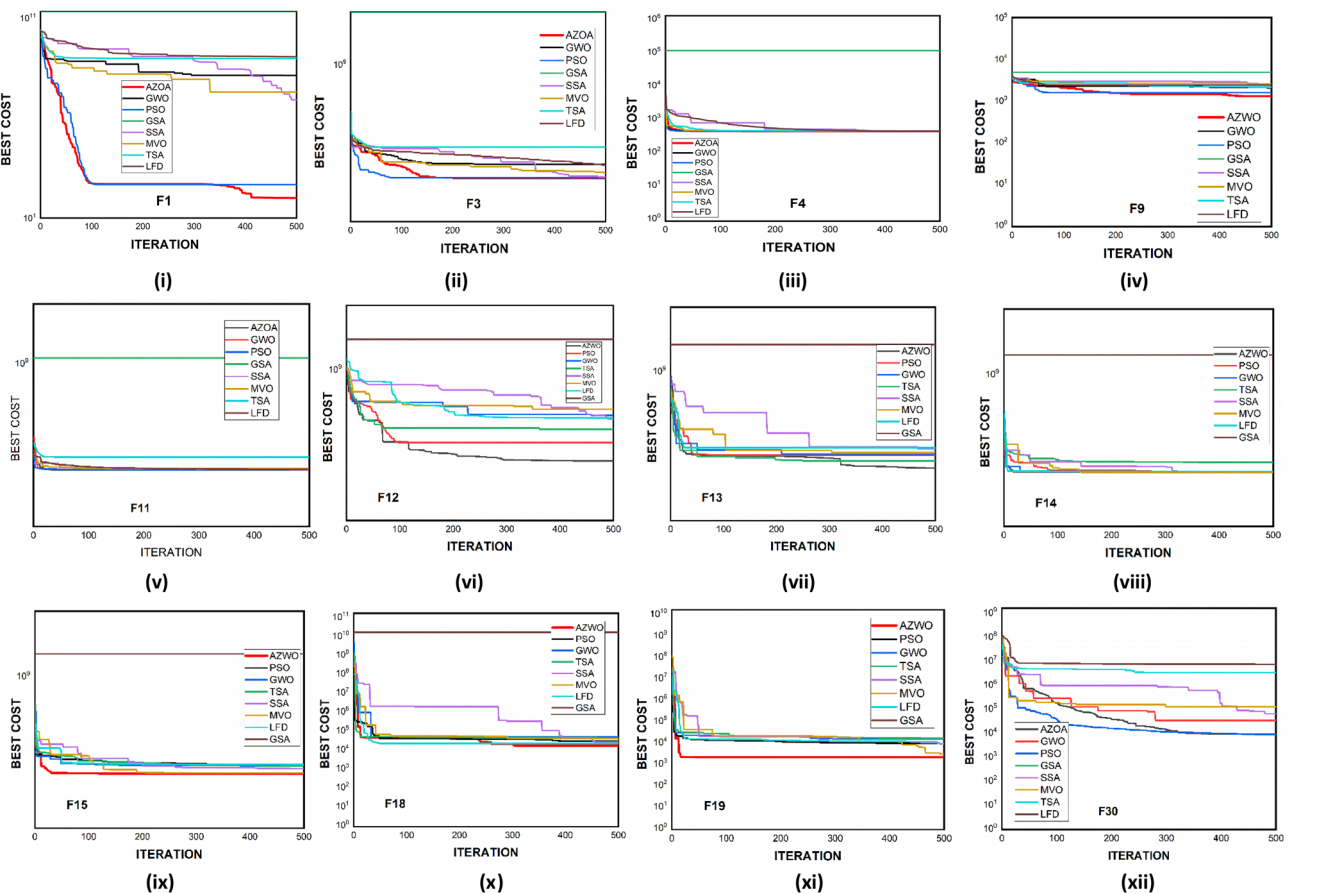
Convergence graph of AZOA and other metaheuristics in solving CEC-2017 benchmark functions.

**Figure 10:**
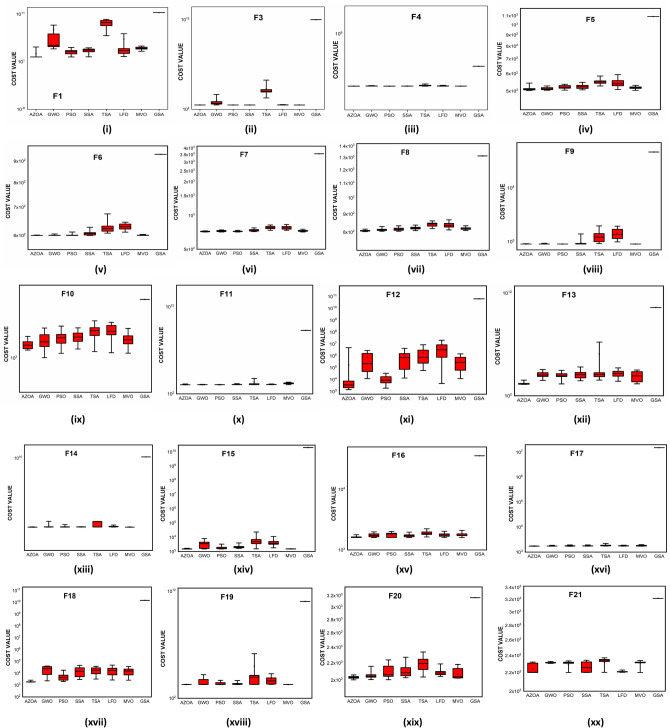

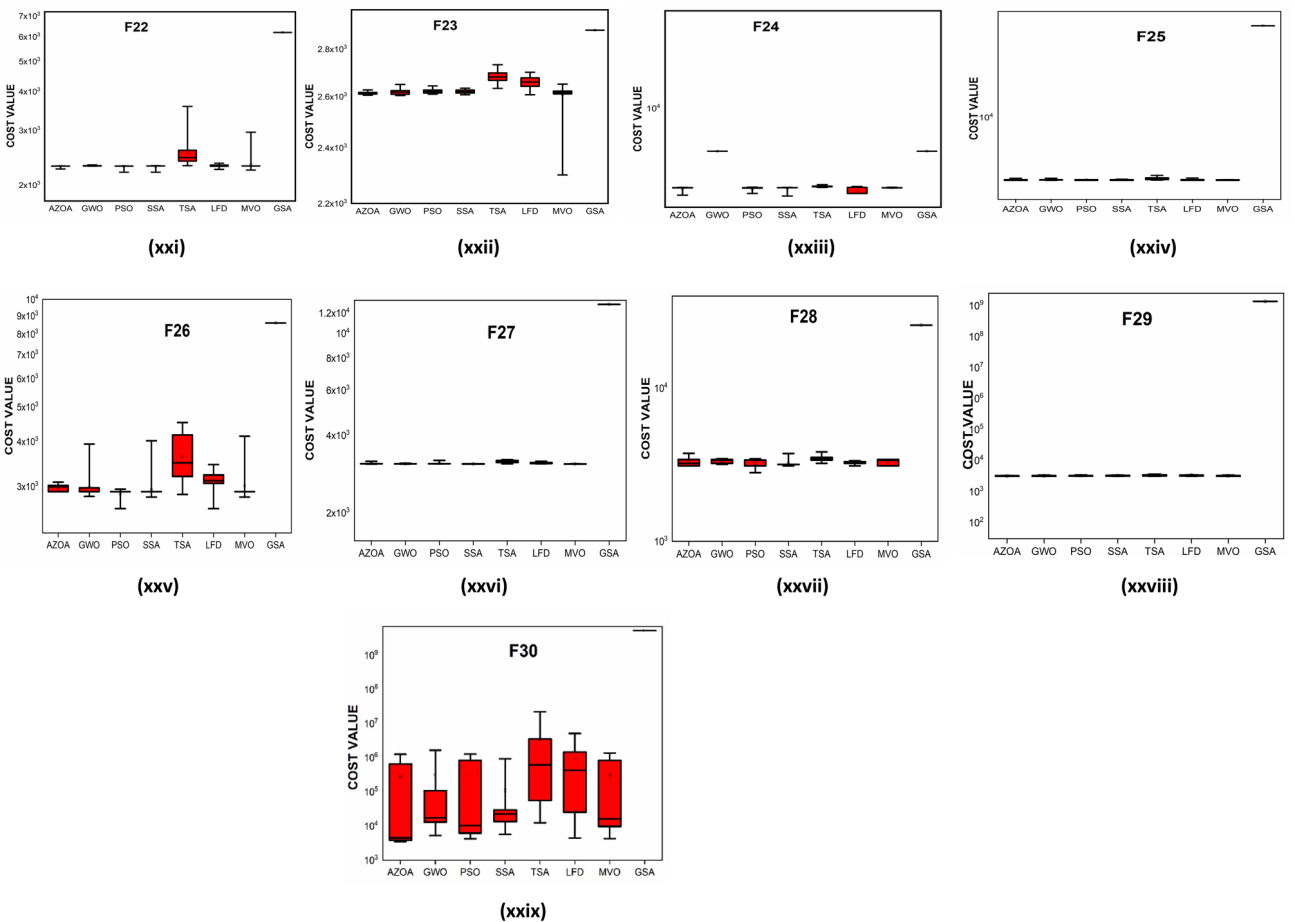
Box plots of AZOA and other metaheuristics in solving CEC-2017 benchmark functions.

In this section, the CEC-2017 test suite functions are employed to evaluate the efficiency and capacity of the newly proposed AZOA. The test suite contains thirty functions from which the function $\mathrm{F}2$ is excluded due to the difficulty in simulation. The CEC-2017 functions are classified into four groups, namely unimodal ($\mathrm{F}1{-}\mathrm{F}3$), multimodal ($\mathrm{F}4{-}\mathrm{F}10$), hybrid ($\mathrm{F}11{-}\mathrm{F}20$), and composition ($\mathrm{F}21{-}\mathrm{F}30$). The hybrid and composite functions reflect more challenging optimization functions with dynamical search spaces that have been used to study the trade-off balance between the exploration and exploitation of the algorithm. In this test function, the dimension is fixed to $10$, and the run times for all algorithms are considered as 30, along with 500 generations, for a total of 150,000 number function evaluations (NFEs). The statistical outcomes of AZOA on the CEC-2017 objective functions are presented in Table 7, and the best outcomes are highlighted in bold letters. Table 7 shows that the proposed algorithm has good performance on unimodal problems and multimodal problems, as well as the ability to identify the global optimal solution continuously. Also, it shows that the AZOA algorithm performed well in comparison to other existing algorithms on hybrid functions. In addition, the outcomes of the fourth group of CEC-2017 functions display that the AZOA produces competitive outcomes in the composition functions. However, comparing metaheuristic algorithms based on their $ave$ and $std$ values is inconclusive. Therefore, $t$ test and the Wilcoxon rank-sum test and at $\alpha$ = 0.05% significant level is presented to demonstrate a significant difference in AZOA. The $t$-values at $\alpha$ = 0.05% level of significance by $t$ test are presented in Table 7 to confirm the presence of significant differences in AZOA with respect to the compared algorithms. If the corresponding $t$-value is boldfaced, AZOAs perform significantly better in comparison to other algorithms. In a tie situation, the results are displayed in bold italic letters. Furthermore, $w/t/l$ has been labelled in the last rows of Table 7, which indicate AZOA win, tie, and lose counts over that certain algorithm in terms of $t$-values. Clearly, from Table 7, it is observed that AZOA has a significant difference over other algorithms. The $p$ values at $\alpha$ = 0.05% significant level by the Wilcoxon Rank Sum test are presented in Table 8 for unimodal, multimodal, and fixed-point multimodal functions, respectively. These tables show that the $p$ values are less than 0.05. This shows clearly that the American zebra algorithm performs better in comparison to other metaheuristic algorithms. The convergent graphs of the implemented algorithms are shown in Fig. 9. When looking at all these curves, it becomes clear that the AZOA shows the rapid convergence for the functions $\mathrm{F}1$, $\mathrm{F}10$, $\mathrm{F}12$, $\mathrm{F}13$, $\mathrm{F}15$, $\mathrm{F}18$, $\mathrm{F}19$, and $\mathrm{F}30$ and a comparable convergence for the functions $\mathrm{F}3$, $\mathrm{F}4$, $\mathrm{F}11$, $\mathrm{F}14$, and $\mathrm{F}15$. As a result of this observation, AZOA may be regarded as one of the dependable algorithms. In Fig. 10, the performance of the metaheuristic algorithms and the proposed AZOA in solving the functions $\mathrm{F}1{-}\mathrm{F}30$ is presented as a boxplot. In optimizing most $\mathrm{F}1{-}\mathrm{F}30$ functions, this boxplot study indicates that the AZOA has a smaller width and more efficient centre than competitor metaheuristic algorithms. This suggests that the AZOA has provided solutions that are almost identical in multiple implementations. As a result, AZOA can offer more effective solutions to optimum challenges. Analysis of the CEC-2017 optimization results demonstrates that AZOA performs better than the seven compared algorithms.

**Table 7:** Statistical outcomes of AZOA and other algorithms in solving CEC-2017 benchmark functions.

Function	AZOA	GWO	PSO	GSA	SSA	MVO	TSA	LFD
	$avg (std)$	$avg\left(std\right)$ $t$-$values$	$avg (std)$ $t$-$values$	$avg (std)$ $t$-$values$	$avg (std)$ $t$-$values$	$avg (std)$ $t$-$values$	$avg (std)$ $t$-$values$	$avg (std)$ $t$-$values$
$\mathrm{F}1$	3.080E+03 (3.31E+03)	5.390E+07 (1.250E+08) **(−3.090E+00)**	1.740E+03 (2.190E+03) **(−5.910E+00)**	2.098E+11 (9.320E−05) **(−7.880E+14)**	3.010E+03 (2.340E+03) **(−9.630E+00)**	7.900E+03 (4.410E+03) **(−1.330E+01)**	2.730E+09 (2.700E+09) **(−7.92E+00)**	5.750E+05 (1.810E+06) **(−2.460E+00)**
$\mathrm{F}3$	3.000E+02 (4.34E+01)	9.800E+02 (1.260E+03) **(−4.2500E+00)**	3.000E+02 (0.000E+00) **(*0.000E+000)***	9.740E+11 (3.730E−04) **(−2.030E+16)**	3.000E+02 (8.600E−10 **(*0.000E+00*)**	3.000E+02 (7.650E−03) **(−1.410E+01)**	1.980E+04 (3.520E+04) **(−4.370E+00)**	3.050E+02 (6.850E+00) **(−5.650E+00)**
$\mathrm{F}4$	4.100E+02 (1.60E+01)	4.160E+02 (1.760E+01) **(−6.630E+00)**	4.120E+02 (5.720E−01) **(−6.550E+00)**	1.080E+05 (2.960E−11) **(−1.190E+06)**	4.150E+02 (1.970E+00) **(−1.520E+01)**	4.140E+02 (1.230E+00) **(−1.410E+01)**	4.920E+02 (7.820E+01) **(−9.150E+00)**	4.240E+02 (3.230E+01) **(−5.580E+00)**
$\mathrm{F}5$	5.360E+02 (1.43E+01)	5.130E+02 (6.680E+00) **(−6.490E+00)**	5.210E+02 (9.410E+00) **(−1.140E+01)**	1.090E+03 (6.940E−13) **(−2.150E+03)**	5.230E+02 (1.070E+01) **(−1.130E+01)**	5.170E+02 (5.950E+00) **(−1.250E+01)**	5.520E+02 (1.410E+01) **(−2.410E+01)**	5.430E+02 (1.930E+01) **(−1.440E+01)**
$\mathrm{F}6$	6.180E+02 (8.58E+00)	6.000E+02 (9.260E−01) **(−3.190E+00)**	6.010E+02 (2.470E+00) **(−2.990E+00)**	9.340E+02 (4.630E−13) **(−1.070E+04)**	6.080E+02 (7.640E+00) **(−7.710E+00)**	6.010E+02 (7.650E−01) **(−6.150E+00)**	6.250E+02 (1.420E+01) **(−1.380E+01)**	6.300E+02 (9.930E+00) **(−2.300E+01)**
$\mathrm{F}7$	7.620E+02 (2.40E+01)	7.260E+02 (9.010E+00) **(−7.400E+00)**	7.220E+02 (5.100E+00) **(−6.210E+00)**	3.560E+03 (1.390E−12) **(−7.010E+03)**	7.370E+02 (1.540E+01) **(−1.010E+01)**	7.300E+02 (1.020E+01) **(−9.400E+00)**	7.800E+02 (2.380E+01) **(−2.000E+01)**	7.770E+02 (2.560E+01) **(−1.790E+01)**
$\mathrm{F}8$	8.270E+02 (9.30E+00)	8.130E+02 (5.310E+00) **(−7.800E+00)**	8.160E+02 (6.810E+00) **(−1.010E+01)**	1.310E+03 (6.940E−13) **(−2.430E+03)**	8.220E+02 (6.620E+00) **(−1.670E+01)**	8.190E+02 (5.980E+00) **(−1.460E+01)**	8.420E+02 (1.160E+01) **(−2.360E+01)**	8.370E+02 (1.420E+01) **(−1.630E+01)**
$\mathrm{F}9$	1.100E+03 (1.20E+02)	9.050E+02 (9.020E+00) **(−4.260E+00)**	9.000E+02 (1.660E−01) ***(−1.100E+00*)**	4.670E+04 (2.220E−11) **(−7.000E+05)**	9.430E+02 (1.050E+02) **(−3.160E+00)**	9.000E+02 (3.130E−01) ***(−1.530E+00*)**	1.240E+03 (2.840E+02) **(−9.270E+00)**	1.420E+03 (3.020E+02) **(−1.330E+01)**
$\mathrm{F}10$	1.990E+03 (3.51E+02)	1.590E+03 (3.160E+02) **(−4.250E+00)**	1.690E+03 (2.930E+02) **(−7.020E+00)**	4.780E+03 (1.850E−12) **(−1.880E+02)**	1.750E+03 (2.510E+02) **(−9.450E+00)**	1.640E+03 (2.700E+02) **(−6.200E+00)**	2.050E+03 (3.120E+02) **(−1.410E+01)**	2.050E+03 (3.370E+02) **(−1.380E+01)**
$\mathrm{F}11$	1.180E+03 (5.46E+01)	1.130E+03 (2.410E+01) **(−5.800E+00)**	1.130E+03 (1.730E+01) **(−6.400E+00)**	1.920E+09 (4.85E−07) **(−2.380E+09)**	1.190E+03 (8.590E+01) **(−6.800E+00)**	1.640E+03 (2.600E+02) **(−5.110E+00)**	2.280E+03 (1.810E+003) **(−4.770E+00)**	1.200E+03 (6.070E+01) **(−1.120E+01)**
$\mathrm{F}12$	2.270E+04 (2.83E+04)	7.450E+05 (9.190E+05) **(−6.360E+00)**	3.200E+04 (8.500E+03) **(−7.020E+00)**	6.390E+10 (2.33E−05) **(−1.570E+08)**	1.030E+06 (1.150E+06) **(−6.910E+00)**	4.670E+05 (4.710E+05) **(−7.750E+00)**	1.760E+06 (2.240E+06) **(−6.180E+00)**	5.300E+06 (5.940E+06) **(−6.910E+00)**
$\mathrm{F}13$	4.960E+03 (8.07E+03)	1.280E+04 (7.520E+03) **(−1.180E+01)**	1.150E+04 (7.060E+03) **(−1.090E+01)**	3.940E+10 (7.760E−06) **(−1.760E+09)**	1.470E+04 (1.200E+04) **(−8.520E+00)**	1.240E+04 (1.090E+04) **(−7.900E+00)**	1.150E+06 (4.380E+06) **(−2.060E+00)**	1.780E+04 (1.280E+04) **(−9.920E+00)**
$\mathrm{F}14$	1.510E+03 (5.64E+01)	2.340E+03 (1.580E+03) **(−4.470E+00)**	1.580E+03 (2.740E+02) **(−4.150E+00)**	1.300E+10 (5.820E−06) **(−5.300E+09)**	1.490E+03 (2.8800E+01) **(−1.300E+01)**	1.440E+03 (1.130E+01) **(−1.950E+00)**	3.7400E+03 (1.880E+003) **(−9.5100E+00)**	1.650E+003 (1.960E+02) **(−8.540E+00)**
$\mathrm{F}15$	1.900E+03 (1.03E+03)	3.640E+03 (2.050E+03) **(−8.000E+00)**	1.840E+03 (4.590E+02) **(−5.510E+00)**	2.020E+10 (0.000E+00) **(−6.680E+09)**	2.130E+03 (4.580E+02) **(−1.040E+01)**	1.530E+03 (1.640E+01) **(−3.010E+00)**	7.190E+03 (6.620E+03) **(−6.720E+00)**	4.570E+03 (2.250E+03) **(−1.050E+01)**
$\mathrm{F}16$	1.780E+03 (1.45E+02)	1.730E+03 (1.090E+02) **(−6.030E+00)**	1.800E+03 (1.440E+02) **(−8.130E+00)**	3.570E+04 (1.480E−11) **(−5.410E+03)**	1.740E+03 (1.050E+02) **(−6.690E+00)**	1.770E+03 (1.300E+02) **(−7.100E+00)**	1.890E+03 (1.190E+02) **(−1.480E+01)**	1.770E+03 (1.220E+02) **(−7.610E+00)**
$\mathrm{F}17$	1.760E+03 (2.54E+01)	1.750E+03 (2.880E+01) **(−4.870E+00)**	1.770E+03 (4.500E+01) **(−6.140E+00)**	1.520E+07 (1.14E−08) **(−9.890E+06)**	1.780E+03 (3.660E+01) **(−9.100E+00)**	1.800E+03 (7.170E+01) **(−7.530E+00)**	1.860E+03 (1.180E+02) **(−8.690E+00)**	1.780E+03 (3.180E+01) **(−1.050E+01)**
$\mathrm{F}18$	1.100E+04 (1.34E+04)	2.280E+04 (1.380E+04) **(−1.100E+001)**	5.980E+03 (4.640E+03) **(−6.930E+00)**	1.480E+10 (7.760E−06) **(−5.210E+09)**	1.820E+04 (1.390E+04) **(−9.110E+00)**	1.600E+04 (9.730E+03) **(−1.1500E+001)**	1.980E+04 (1.150E+04) **(−1.220E+001)**	1.900E+04 (1.300E+04) **(−9.480E+00)**
$\mathrm{F}19$	2.050E+03 (2.00E+02)	4.880E+03 (5.220E+03) **(−4.470E+00)**	2.460E+03 (8.600E+02) **(−5.010E+00)**	1.110E+11 (3.100E−05) **(−5.030E+10)**	2.360E+03 (7.300E+02) **(−4.800E+00)**	1.910E+03 (7.350E+00) **(−1.730E+00)**	9.920E+04 (2.780E+05) **(−2.750E+00)**	5.710E+03 (4.280E+03) **(−6.870E+00)**
$\mathrm{F}20$	2.130E+03 (7.06E+01)	2.060E+03 (4.010E+01) **(−4.860E+00)**	2.100E+03 (7.150E+01) **(−7.300E+00)**	3.160E+03 (1.390E−12) **(−6.540E+02)**	2.100E+03 (6.750E+01) **(−8.230E+00)**	2.070E+03 (5.550E+01) **(−4.820E+00)**	2.190E+03 (8.170E+01) **(−1.440E+01)**	2.090E+03 (3.790E+01) **(−1.170E+01)**
$\mathrm{F}21$	2.250E+03 (6.81E+01)	2.310E+03 (6.340E+00) **(−1.090E+01)**	2.290E+03 (5.160E+01) **(−5.250E+00)**	3.210E+03 (4.630E−13) **(−1.430E+02)**	2.270E+03 (6.110E+01) **(−2.050E+00)**	2.300E+03 (3.760E+01) **(−8.040E+00)**	2.320E+03 (6.000E+01) **(−7.570E+00)**	2.250E+03 (5.430E+00) (4.500E+00)
$\mathrm{F}22$	2.310E+03 (1.49E+01)	2.310E+03 (5.810E+00) **(−5.350E+00)**	2.300E+03 (1.890E+01) **(*6.28E−01)***	6.200E+03 (9.25E−13) **(−3.430E+03)**	2.290E+03 (2.520E+01) ***(1.680E+00)***	2.320E+03 (1.230E+02) ***(−1.500E+00)***	2.540E+03 (2.9800E+02) **(−6.3000E+000)**	2.310E+03 (2.400E+01) **(−1.720E+00)**
$\mathrm{F}23$	2.640E+03 (2.72E+01)	2.620E+03 (1.110E+01) **(−5.650E+00)**	2.620E+03 (8.020E+00) **(−8.730E+00)**	2.880E+03 (4.630E−13) **(−3.720E+02)**	2.620E+03 (8.160E+00) **(−8.050E+00)**	2.610E+03 (5.980E+01) ***(2.210E−001*)**	2.670E+03 (2.320E+01) **(−2.180E+001)**	2.660E+03 (2.200E+01) **(−1.690E+01)**
$\mathrm{F}24$	2.730E+03 (1.05E+02)	2.740E+03 (9.450E+00) **(−2.040E+00)**	2.700E+03 (8.930E+01) ***(1.220E+00*)**	4.980E+03 (3.710E−12) **(−2.090E+02)**	2.730E+03 (7.730E+01) ***(−6.30E−01*)**	2.740E+03 (7.440E+00) **(−2.310E+00)**	2.810E+03 (3.140E+01) **(−7.560E+00)**	2.590E+03 (1.240E+02) (6.670E+00)
$\mathrm{F}25$	2.920E+03 (6.73E+01)	2.940E+03 (2.430E+001) **(−1.640E+00)**	2.930E+03 (2.310E+01) **(*7.170E−01*)**	6.040E+04 (2.220E−11) **(−1.510E+04)**	2.920E+03 (2.520E+01) **(*1.140E+00*)**	2.920E+03 (2.350E+01) (2.100E+00)	3.010E+03 (7.600E+01) **(−7.230E+00)**	2.950E+03 (4.130E+01) **(−2.310E+00)**
$\mathrm{F}26$	3.090E+03 (3.24E+02)	2.980E+03 (2.500E+02) **(−2.85E−01)**	2.860E+03 (9.600E+01) (7.800E+00)	8.590E+03 (5.550E−12) **(−7.500E+02)**	2.940E+03 (2.050E+02) **(*1.140E+00*)**	3.010E+03 (3.300E+02) ***(−9.52E−01)***	3.620E+03 (5.350E+02) **(−9.490E+00)**	3.120E+03 (1.540E+02) **(−7.200E+00)**
$\mathrm{F}27$	3.130E+03 (3.08E+01)	3.090E+03 (4.960E+00) (2.800E+00)	3.110E+03 (2.310E+01) **(−1.940E+00)**	1.300E+04 (7.400E−12) **(−5.610E+03)**	3.090E+03 (2.540E+00) (4.690E+00**)**	3.090E+03 (1.990E+00) (5.200E+00)	3.160E+03 (3.3600E+01) **(−1.290E+001)**	3.120E+03 (1.710E+01) **(−7.0200E+00)**
$\mathrm{F}28$	3.330E+03 (1.49E+02)	3.340E+03 (1.030E+02) **(−2.280E+00)**	3.210E+03 (1.820E+02) (2.320E+00)	2.580E+04 (3.70E−12) **(−1.060E+03)**	3.200E+03 (1.340E+02) (2.800E+00**)**	3.300E+03 (1.400E+02) ***(−8.03E−01)***	3.450E+03 (1.550E+02) **(−5.860E+00)**	3.260E+03 (6.390E+01) **(*7.23E−01*)**
$\mathrm{F}29$	3.260E+03 (6.88E+01)	3.180E+03 (4.940E+01) **(−1.810E+00)**	3.200E+03 (3.860E+01) **(−5.500E+00)**	1.350E+09 (1.210E−06) **(−4.530E+08)**	3.210E+03 (5.160E+01) **(−5.010E+00)**	3.190E+03 (4.770E+01) **(−3.470E+00)**	3.290E+03 (9.300E+01) **(−8.860E+00)**	3.260E+03 (6.440E+01) **(−1.070E+001)**
$\mathrm{F}30$	1.560E+06 (1.73E+06)	3.070E+05 (5.780E+05) ***(−2.90E−001)***	3.100E+05 (4.820E+05) ***(−3.50E−001)***	5.240E+09 (9.700E−07) **(−9.370E+04)**	1.090E+05 (2.680E+05) (2.600E+00)	2.960E+05 (4.770E+05) ***(−1.93E−01)***	3.400E+06 (5.630E+06) **(−4.330E+00)**	9.470E+05 (1.320E+06) **(−3.730E+00)**
$w/t/l$	28/2/0		22/6/2	30/0/0	22/4/4	22/6/2	30/0/0	27/1/2

Significant values are in bold/italic.

**Table 8:** $p$ values by Wilcoxon Rank Sum test with five percent significance level for $\mathrm{F}1{-}\mathrm{F}30$.

Function	GWO versus AZOA		PSO versus AZOA		TSA versus AZOA		SSA versus AZOA		MVO versus AZOA		LFD versus AZOA		GSA versus AZOA	
	$p$ $\mathrm{values}$	$H$	$p$ $\mathrm{values}$	$H$	$p$ $\mathrm{values}$	$H$	$p$ $\mathrm{values}$	$H$	$p$ $\mathrm{values}$	$H$	$p$ $\mathrm{values}$	$H$	$p$ $\mathrm{values}$	$H$
$\mathrm{F}1$	$1.780\mathrm{E}{-}06$	1	$3.180E{-}05$	$1$	$1.780E{-}06$	$1$	$1.680E{-}05$	$1$	$3.980E{-}06$	$1$	$2.210E{-}05$	$1$	$1.400E{-}06$	$1$
$\mathrm{F}3$	$1.780\mathrm{E}{-}06$	1	$NA$	$0$	$1.780E{-}06$	$1$	$NA$	$0$	$1.780E{-}06$	$1$	$1.780E{-}06$	$1$	$4.470E{-}08$	$1$
$\mathrm{F}4$	$1.780\mathrm{E}{-}06$	1	$8.810E{-}01$	$0$	$1.970E{-}06$	$1$	$1.970E{-}06$	$1$	$1.050E{-}05$	$1$	$2.950E{-}06$	$1$	$1.780E{-}06$	$1$
$\mathrm{F}5$	$3.920\mathrm{E}{-}02$	1	$2.010E{-}04$	$1$	$1.780E{-}06$	$1$	$2.020E{-}05$	$1$	$4.280E{-}04$	$1$	$2.950E{-}06$	$1$	$1.750E{-}06$	$1$
$\mathrm{F}6$	$1.310\mathrm{E}{-}03$	1	$2.310E{-}01$	$0$	$1.780E{-}06$	$1$	$2.410E{-}06$	$1$	$2.210E{-}05$	$1$	$1.780E{-}06$	$1$	$1.770E{-}06$	$1$
$\mathrm{F}7$	$4.180\mathrm{E}{-}03$	1	$1.800E{-}01$	$0$	$1.780E{-}06$	$1$	$1.840E{-}05$	$1$	$1.050E{-}03$	$0$	$1.780E{-}06$	$1$	$1.780E{-}06$	$1$
$\mathrm{F}8$	$4.970\mathrm{E}{-}05$	1	$6.040E{-}06$	$1$	$1.780E{-}06$	$1$	$1.780E{-}06$	$1$	$2.950E{-}06$	$1$	$1.780E{-}06$	$1$	$1.630E{-}06$	$1$
$\mathrm{F}9$	$5.070\mathrm{E}{-}03$	1	$2.010E{-}04$	$1$	$1.780E{-}06$	$1$	$5.580E{-}05$	$1$	$5.890E{-}01$	$0$	$1.780E{-}06$	$1$	$1.030E{-}06$	$1$
$\mathrm{F}10$	$2.340\mathrm{E}{-}02$	1	$9.800E{-}04$	$1$	$2.180E{-}06$	$1$	$8.370E{-}05$	$1$	$1.310E{-}03$	$1$	$2.410E{-}06$	$1$	$1.780E{-}06$	$1$
$\mathrm{F}11$	$1.280E{-}04$	$1$	$5.390E{-}04$	$1$	$1.050E{-}05$	$1$	$2.660E{-}05$	$1$	$1.780E{-}06$	$1$	$1.270E{-}05$	$1$	$1.730E{-}06$	$1$
$\mathrm{F}12$	$1.780E{-}06$	$1$	$1.780E{-}06$	$1$	$7.290E{-}04$	$1$	$1.780E{-}06$	$1$	$9.540E{-}06$	$1$	$1.150E{-}05$	$1$	$1.780E{-}06$	$1$
$\mathrm{F}13$	$1.780E{-}06$	$1$	$2.180E{-}06$	$1$	$1.780E{-}06$	$1$	$1.780E{-}06$	$1$	$5.350E{-}06$	$1$	$1.780E{-}06$	$1$	$1.780E{-}06$	$1$
$\mathrm{F}14$	$2.670E{-}06$	$1$	$1.970E{-}06$	$1$	$2.410E{-}06$	$1$	$1.780E{-}06$	$1$	$3.010E{-}01$	$0$	$1.780E{-}06$	$1$	$1.780E{-}06$	$1$
$\mathrm{F}15$	$3.260E{-}06$	$1$	$4.390E{-}06$	$1$	$1.780E{-}06$	$1$	$1.780E{-}06$	$1$	$2.560E{-}01$	$0$	$1.780E{-}06$	$1$	$1.780E{-}06$	$1$
$\mathrm{F}16$	$1.280E{-}04$	$1$	$1.080E{-}04$	$1$	$1.780E{-}06$	$1$	$7.040E{-}05$	$1$	$4.550E{-}05$	$1$	$1.390E{-}04$	$1$	$1.780E{-}06$	$1$
$\mathrm{F}17$	$7.290E{-}04$	$1$	$3.960E{-}04$	$1$	$2.180E{-}06$	$1$	$4.390E{-}06$	$1$	$8.670E{-}06$	$1$	$1.970E{-}06$	$1$	$1.780E{-}06$	$1$
$\mathrm{F}18$	$1.780E{-}06$	$1$	$2.670E{-}06$	$1$	$1.780E{-}06$	$1$	$1.780E{-}06$	$1$	$1.780E{-}06$	$1$	$1.780E{-}06$	$1$	$1.780E{-}06$	$1$
$\mathrm{F}19$	$3.600E{-}06$	$1$	$1.970E{-}06$	$1$	$2.410E{-}06$	$1$	$2.670E{-}06$	$1$	$1.860E{-}03$	$1$	$1.780E{-}06$	$1$	$1.780E{-}06$	$1$
$\mathrm{F}20$	$4.990E{-}04$	$1$	$8.670E{-}06$	$1$	$1.970E{-}06$	$1$	$8.670E{-}06$	$1$	$3.040E{-}02$	$1$	$1.780E{-}06$	$1$	$1.780E{-}06$	$1$
$\mathrm{F}21$	$8.670E{-}06$	$1$	$5.390E{-}04$	$1$	$7.040E{-}05$	$1$	$5.260E{-}02$	$0$	$3.480E{-}05$	$1$	$6.470E{-}01$	$0$	$1.650E{-}06$	$1$
$\mathrm{F}22$	$5.890E{-}06$	$1$	$4.550E{-}02$	$1$	$1.780E{-}06$	$1$	$2.890E{-}02$	$1$	$2.910E{-}05$	$1$	$9.070E{-}02$	$0$	$1.780E{-}06$	$1$
$\mathrm{F}23$	$5.780E{-}02$	$0$	$9.800E{-}04$	$1$	$1.780E{-}06$	$1$	$2.630E{-}03$	$1$	$2.230E{-}01$	$0$	$2.180E{-}06$	$1$	$1.780E{-}06$	$1$
$\mathrm{F}24$	$1.540E{-}01$	$0$	$1.860E{-}01$	$0$	$1.970E{-}06$	$1$	$3.960E{-}01$	$0$	$5.890E{-}01$	$0$	$1.510E{-}03$	$1$	$1.780E{-}06$	$1$
$\mathrm{F}25$	$9.470E{-}01$	$0$	$1.930E{-}01$	$0$	$9.920E{-}05$	$1$	$1.860E{-}01$	$0$	$9.990E{-}03$	$1$	$1.370E{-}01$	$0$	$1.770E{-}06$	$1$
$\mathrm{F}26$	$3.550E{-}02$	$1$	$5.810E{-}05$	$1$	$3.600E{-}06$	$1$	$2.420E{-}03$	$1$	$1.220E{-}01$	$0$	$9.110E{-}05$	$1$	$1.640E{-}06$	$1$
$\mathrm{F}27$	$3.730E{-}02$	$1$	$1.730E{-}01$	$0$	$1.780E{-}06$	$1$	$2.950E{-}06$	$1$	$5.890E{-}06$	$1$	$7.880E{-}06$	$1$	$1.780E{-}06$	$1$
$\mathrm{F}28$	$1.680E{-}02$	$1$	$5.420E{-}02$	$0$	$9.920E{-}05$	$1$	$7.890E{-}02$	$0$	$4.310E{-}01$	$0$	$8.010E{-}01$	$0$	$1.510E{-}06$	$1$
$\mathrm{F}29$	$8.670E{-}06$	$1$	$5.390E{-}04$	$1$	$7.040E{-}05$	$1$	$5.260E{-}02$	$0$	$3.480E{-}05$	$1$	$6.470E{-}01$	$0$	$1.650E{-}06$	$1$
$\mathrm{F}30$	$5.890E{-}06$	$1$	$4.550E{-}02$	$1$	$1.780E{-}06$	$1$	$2.890E{-}02$	$1$	$2.910E{-}05$	$1$	$9.070E{-}02$	$0$	$1.780E{-}06$	$1$

### Performance of AZOA on the CEC-2019 benchmark test suite

This subsection computes the compared algorithm performance using the new proposed CEC-2019 benchmark functions. For all algorithms, the population size is considered as 30 with 500 iterations and a maximum of 15,000 function evaluations. Its outcomes are compared with the same algorithm that was employed in the previous part. The statistical outcomes such as $avg$ and $std$ are reported in Table 9. According to the $avg$ value, the results of Table 9 show that the new algorithm performs better for solving the benchmark functions in comparison to another algorithm. The $t$-values at $\alpha$ = 0.05% significant level are presented in Table 9 to check the significant difference between the algorithms. Clearly, from Table 9, it is observed that AZOA has a significant difference over other algorithms. The $p$ values by the Wilcoxon rank sum test at $\alpha$ = 0.05% significant are presented in Table 10. Table 10 shows that the $p$-values are smaller than 0.05. This shows clearly that the American zebra optimization algorithm performs well in comparison to other metaheuristic algorithms.

**Table 9:** Statistical outcomes of AZOA and other algorithms in solving CEC-2019 benchmark functions.

Function	AZOA	GWO	PSO	GSA	SSA	MVO	TSA	LFD
	$avg (std)$	$avg (std)$ $t$-$values$	$avg (std)$ $t$-$values$	$avg (std)$ $t$-$values$	$avg (std)$ $t$-$values$	$avg (std)$ $t$-$values$	$avg (std)$ $t$-$values$	$avg (std)$ $t$-$values$
$\mathrm{F}1$	8.160E+04 (1.55E+05)	2.760E+08 (5.310E+08) **(−2.850E**+**00)**	7.020E+08 (6.960E+08) **(−5.520E+00)**	2.590E+12 (1.610E+12) **(−8.810E+00)**	1.560E+10 (2.110E+10) **(−4.050E+00)**	4.110E+09 (2.400E+09) **(−9.380E+00)**	1.670E+08 (2.220E+08) **(−1.130E+01)**	1.120E+05 (1.960E+05) **(**1.120E+05**)**
$\mathrm{F}2$	1.730E+01 (3.48E−05)	1.730E+01 (3.04E−04) ***(0.000E*+*000*)**	1.730E+001 (0.000E+000) ***(0.000E*+*00)***	1.240E+04 (4.650E+03) **(−1.460E+001)**	1.740E+01 (1.67E−02) **(−3.280E+01)**	1.950E+01 (1.440E+00) **(−8.370E+00)**	1.8200E+01 (6.210E−01) **(−7.940E+00)**	1.730E+01 (5.170E−03) (1.730E+01)
$\mathrm{F}3$	1.270E+01 (2.85E−06)	1.270E+01 (1.660E−06) **(*0.000E*+*000)***	1.270E+001 (9.030E−15) **(*0.000E*+*00)***	1.270E+001 (2.830E−04) **(*0.000E*+*000)***	1.270E+01 (4.830E−10) **(*0.000E*+*000*)**	1.270E+001 (3.120E−08) ***(0.000E0*+*00)***	1.270E+001 (9.830E−04) **(*0.000E*+*000*)**	1.270E+01 (3.520E−04) (1.270E+001)
$\mathrm{F}4$	2.670E+02 (1.90E+02)	1.320E+02 (4.310E+02) ***(−1.180E*+*000)***	2.100E+01 (8.920E+00) (4.480E+00)	1.020E+01 (5.650E+00) **(**7.620E+00)	4.300E+01 (1.800E+01) ***(−8.63E−01)***	4.090E+01 (1.010E+001) ***(−5.44E−01)***	4.710E+03 (2.830E+03) **(−9.040E+00)**	1.170E+03 (8.450E+02) (1.170E+03)
$\mathrm{F}5$	1.420E+00 (2.53E−01)	1.4300E+00 (2.490E−001) **(−8.100E+00)**	1.160E+00 (1.210E−01) **(−4.460E+00)**	1.000E+00 (5.950E−03) (1.490E+01)	1.320E+00 (1.620E−01) **(−8.720E+00)**	1.360E+00 (1.110E−01) **(−1.450E+01)**	2.600E+00 (6.980E−01) **(−1.200E+01)**	2.730E+00 (5.770E−01) (2.730E+00)
$\mathrm{F}6$	9.670E+00 (1.14E+00)	1.080E+01 (6.600E−01) **(−2.920E+01)**	7.850E+00 (1.730E+00) **(−6.500E+00)**	1.000E+00 (1.190E−05) (3.580E+01)	5.710E+00 (1.780E+00) ***(−2.290E−01)***	9.050E+00 (1.190E+00) **(−1.350E+01)**	1.050E+01 (8.930E−01) **(−2.340E+01)**	9.290E+00 (9.630E−01) (9.290E+00)
$\mathrm{F}7$	2.450E+02 (2.09E+02)	3.760E+02 (2.550E+02) **(−4.850E+00)**	2.060E+02 (1.220E+02) **(−2.6800E+00)**	1.660E+002 (4.570E+01) **(−1.960E+00)**	3.730E+02 (2.030E+02) **(−5.520E+00)**	3.320E+02 (1.700E+02) **(−5.0800E+00)**	6.570E+02 (2.500E+02) **(−9.900E+00)**	3.370E+02 (1.970E+02) (3.370E+02)
$\mathrm{F}8$	5.060E+00 (6.10E−01)	4.940E+00 (1.080E+00) ***(−1.620E*+*00)***	5.210E+00 (7.110E−01) **(−4.090E+00)**	5.410E+00 (5.040E−01) **(−6.880E+00)**	5.110E+00 (6.240E−01) **(−3.760E+00)**	5.220E+00 (6.050E−01) **(−4.670E+00)**	6.340E+00 (4.640E−01) **(−1.550E+01)**	5.750E+00 (3.950E−01) (5.750E+00)
$\mathrm{F}9$	3.320E+00 (4.90E−01)	4.480E+00 (9.370E−01) **(−1.160E+01)**	2.390E+00 (3.750E−02) (5.230E+00)	3.090E+00 (3.470E−01) **(−9.120E+00)**	2.600E+00 (1.820E−01) **(−2.920E+00)**	2.440E+00 (9.370E−02) (2.020E+00)	7.830E+02 (8.580E+02) **(−4.980E+00)**	5.190E+00 (1.680E+00) (5.190E+00)
$\mathrm{F}10$	1.970E+01 (2.65E+00)	1.930E+01 (4.460E+00) **(*8.500E−01*)**	2.010E+01 (1.440E−01) **(−3.700E+00)**	1.790E+01 (6.100E+00) (1.800E+00)	2.010E+01 (9.650E−02) **(−5.360E+00)**	2.010E+01 (3.440E−02) **(−1.130E+001)**	2.040E+01 (7.510E−02) **(−3.320E+001)**	2.000E+01 (8.220E−04) (2.000E+01)
$w/t/l$		6/4/0	6/2/2	5/1/4	7/3/0	7/2/1	9/1/0	0/0/10

Significant values are in bold/italic.

**Table 10:** $p$-values by Wilcoxon Rank Sum test with five percent significance level for $\mathrm{F}1\mathrm{ to F}10$.

Function	GWO versus AZOA		PSO versus AZOA		TSA versus AZOA		SSA versus AZOA		MVO versus AZOA		LFD versus AZOA		GSA versus AZOA	
	$p$-$values$	$H$	$p$-$values$	$H$	$p$-$values$	$H$	$p$-$values$	$H$	$p$-$values$	$H$	$p$-$values$	$H$	$p$-$values$	$H$
$\mathrm{F}1$	$1.770E{-}06$	$1$	$1.770E{-}06$	$1$	$1.770E{-}06$	$1$	$1.770E{-}06$	$1$	$1.770E{-}06$	$1$	$1.590E{-}02$	$1$	$1.770E{-}06$	$1$
$\mathrm{F}2$	$1.770E{-}06$	$1$	$NA$	$0$	$1.050E{-}05$	$1$	$1.778E{-}06$	$1$	$1.770E{-}06$	$1$	$1.770E{-}06$	$1$	$1.777E{-}06$	$1$
$\mathrm{F}3$	$NA$	$0$	$NA$	$0$	$1.390E{-}04$	$1$	$NA$	$0$	$5.500E{-}02$	$0$	$1.910E{-}03$	$1$	$6.460E{-}01$	$0$
$\mathrm{F}4$	$6.950E{-}02$	$0$	$9.800E{-}04$	$1$	$1.780E{-}06$	$1$	$7.690E{-}01$	$0$	$7.690E{-}01$	$0$	$1.780E{-}06$	$1$	$3.260E{-}06$	$1$
$\mathrm{F}5$	$1.150E{-}05$	$1$	$9.800E{-}04$	$1$	$1.780E{-}06$	$1$	$1.780E{-}06$	$1$	$1.780E{-}06$	$1$	$1.780E{-}06$	$1$	$1.780E{-}06$	$1$
$\mathrm{F}6$	$1.770E{-}06$	$1$	$9.800E{-}04$	$1$	$1.780E{-}06$	$1$	$1.120E{-}01$	$0$	$1.780E{-}06$	$1$	$1.780E{-}06$	$1$	$1.780E{-}06$	$1$
$\mathrm{F}7$	$9.110E{-}05$	$1$	$4.220E{-}02$	$1$	$2.410E{-}06$	$1$	$4.160E{-}05$	$1$	$1.280E{-}04$	$1$	$3.380E{-}04$	$1$	$4.220E{-}02$	$1$
$\mathrm{F}8$	$9.140E{-}01$	$0$	$5.26E{-}02$	$0$	$2.180E{-}06$	$1$	$9.070E{-}02$	$0$	$1.680E{-}02$	$1$	$3.600E{-}06$	$1$	$3.660E{-}04$	$1$
$\mathrm{F}9$	$1.770E{-}06$	$1$	$4.550E{-}05$	$1$	$1.780E{-}06$	$1$	$1.740E{-}03$	$1$	$4.330E{-}02$	$1$	$1.780E{-}06$	$1$	$1.780E{-}06$	$1$
$\mathrm{F}10$	$3.660E{-}04$	$1$	$5.070E{-}03$	$1$	$1.770E{-}06$	$1$	$5.750E{-}01$	$0$	$2.410E{-}06$	$1$	$1.780E{-}06$	$1$	$1.780E{-}06$	$1$

**Figure 11:**
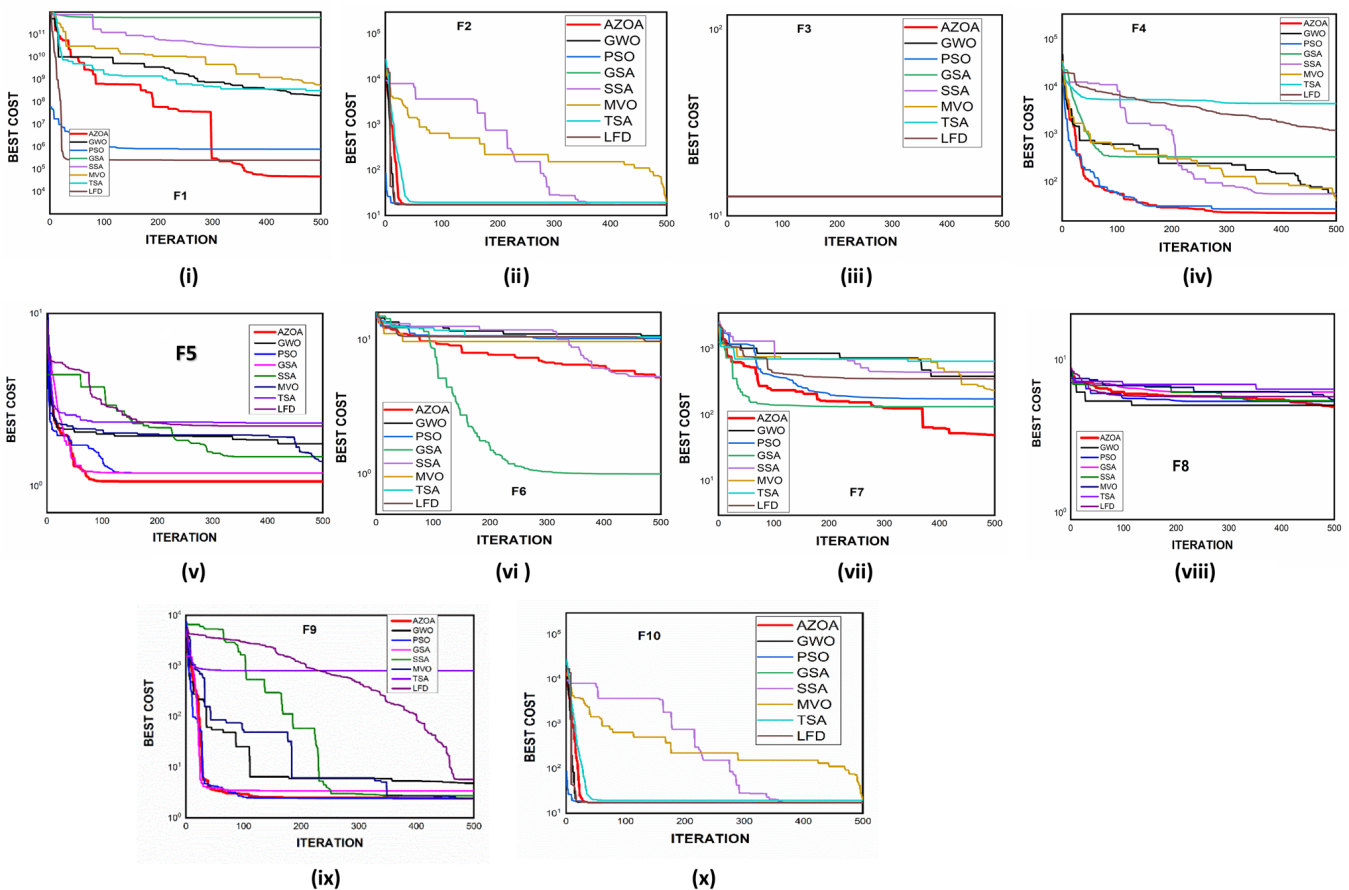
Convergence graph of AZOA and other metaheuristics in solving CEC-2019 benchmark functions.

**Figure 12:**
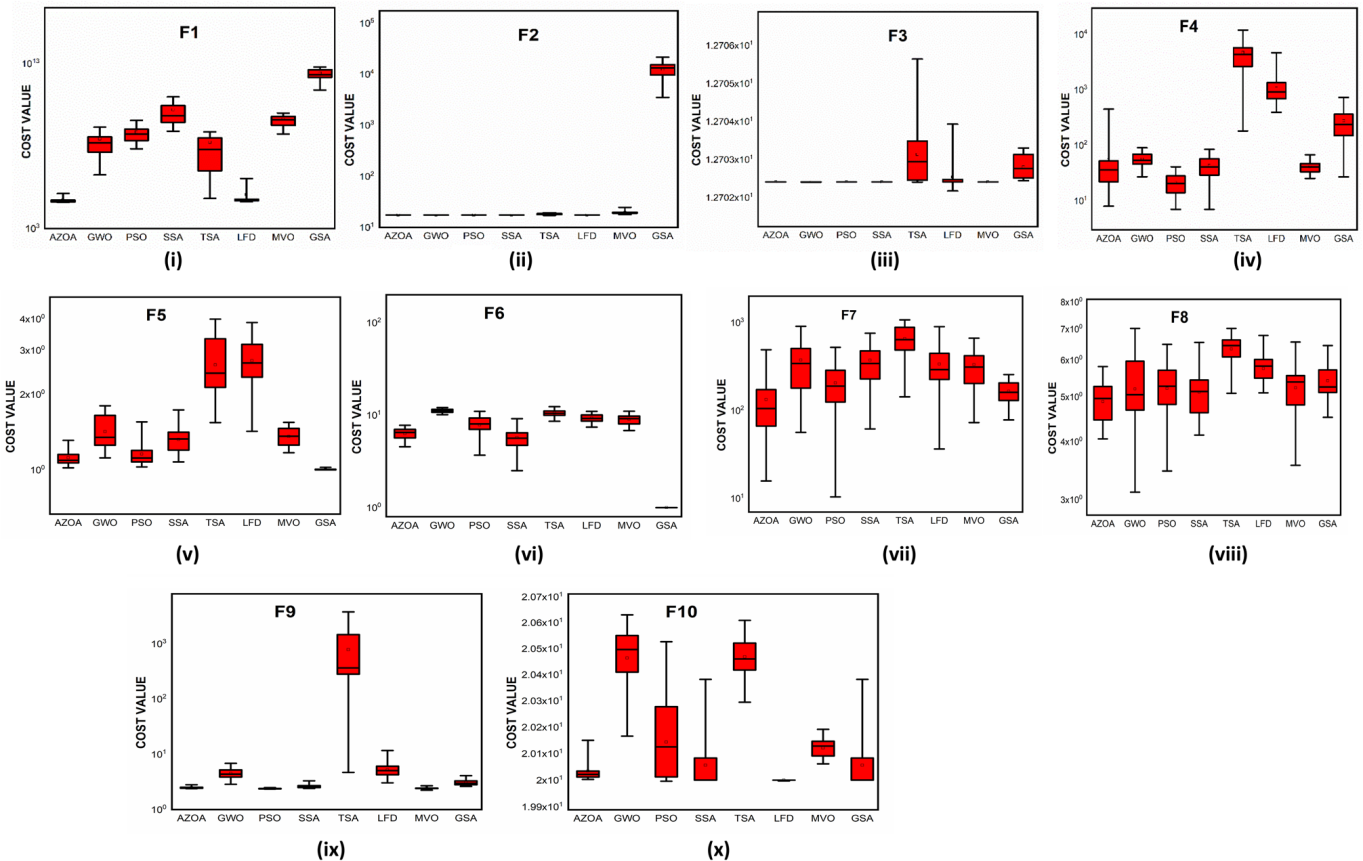
Box plots of AZOA and other metaheuristics in solving CEC-2019 benchmark functions.

The convergent graph of the algorithms that have been implemented is shown in Fig. 11. It is clear from these curves that the AZOA exhibits the quickest convergent for the functions $\mathrm{F}1$, $\mathrm{F}4$, $\mathrm{F}5$, and $\mathrm{F}7$ and a comparable convergence for the functions $\mathrm{F}2$, $\mathrm{F}3$, $\mathrm{F}8$, and $\mathrm{F}9$. In Fig. 12, the boxplot of the compared algorithms along with the proposed AZOA for solving the functions is presented as a boxplot. From Fig. 12, the boxplot study indicates that the AZOA has a smaller width and a more efficient centre than competitor metaheuristic algorithms. This shows that the AZOA has provided solutions that are almost identical in multiple implementations. As a result, AZOA can offer more effective solutions to optimal challenges.

### Comparison of proposed AZOA with latest outstanding algorithms

In this subsection, the performance of the proposed AZOA method is compared to that of the four latest outstanding algorithms, namely the Farmland Fertility Algorithm (FFA)^57^, Mountain Gazelle Optimization (MGO)^48^, African Vultures Optimization Algorithm (AVOA)^42^, and Artificial Gorilla Troops Optimizer (GTO)^47^. The proposed AZOA method and these four latest outstanding algorithms are implemented on the CEC-2005, CEC-2017, and CEC-2019 benchmark functions.

**Figure 13:**
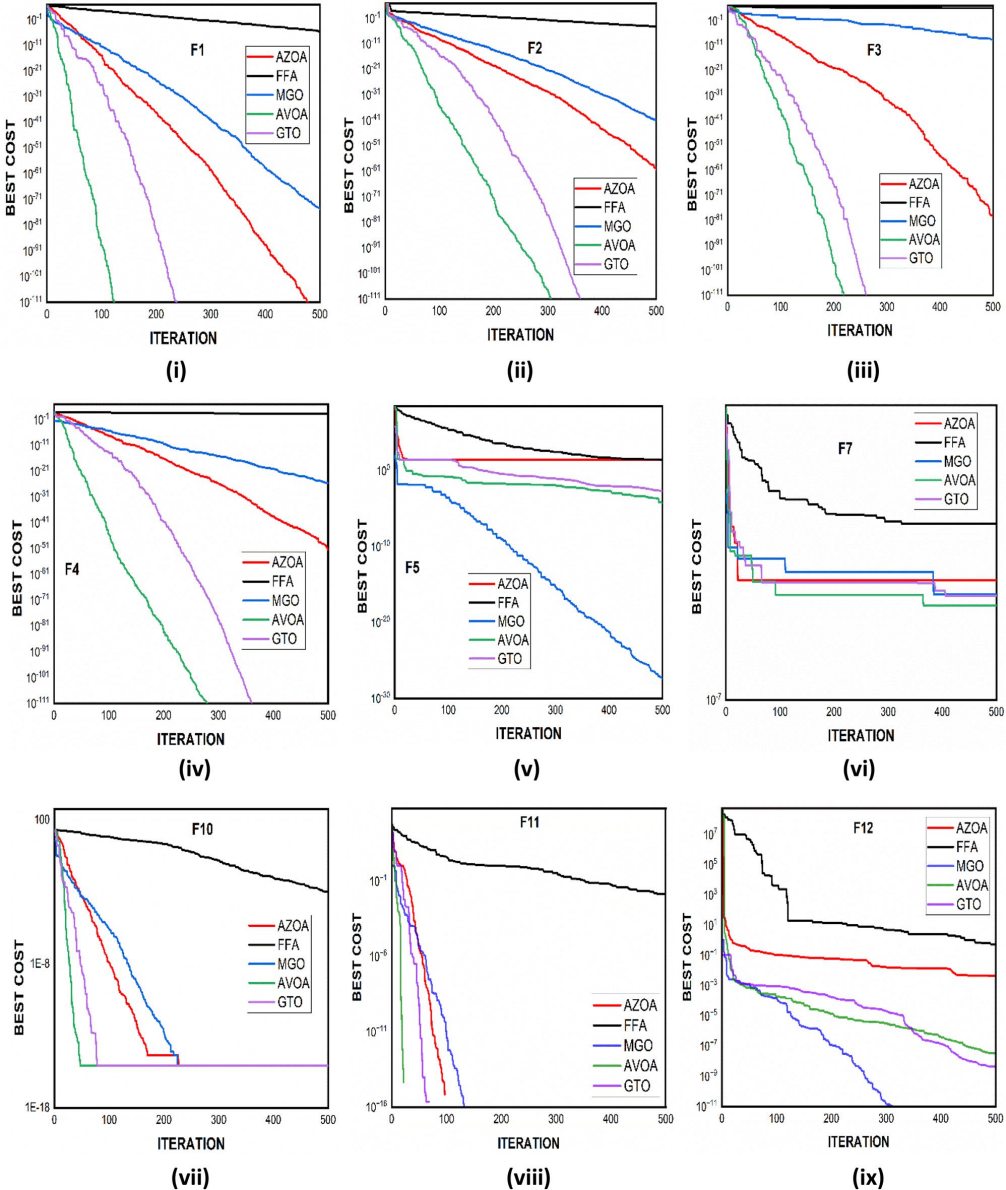
Convergence graph of AZOA and four latest outstanding metaheuristics in solving CEC-2005 benchmark functions.

**Figure 14:**
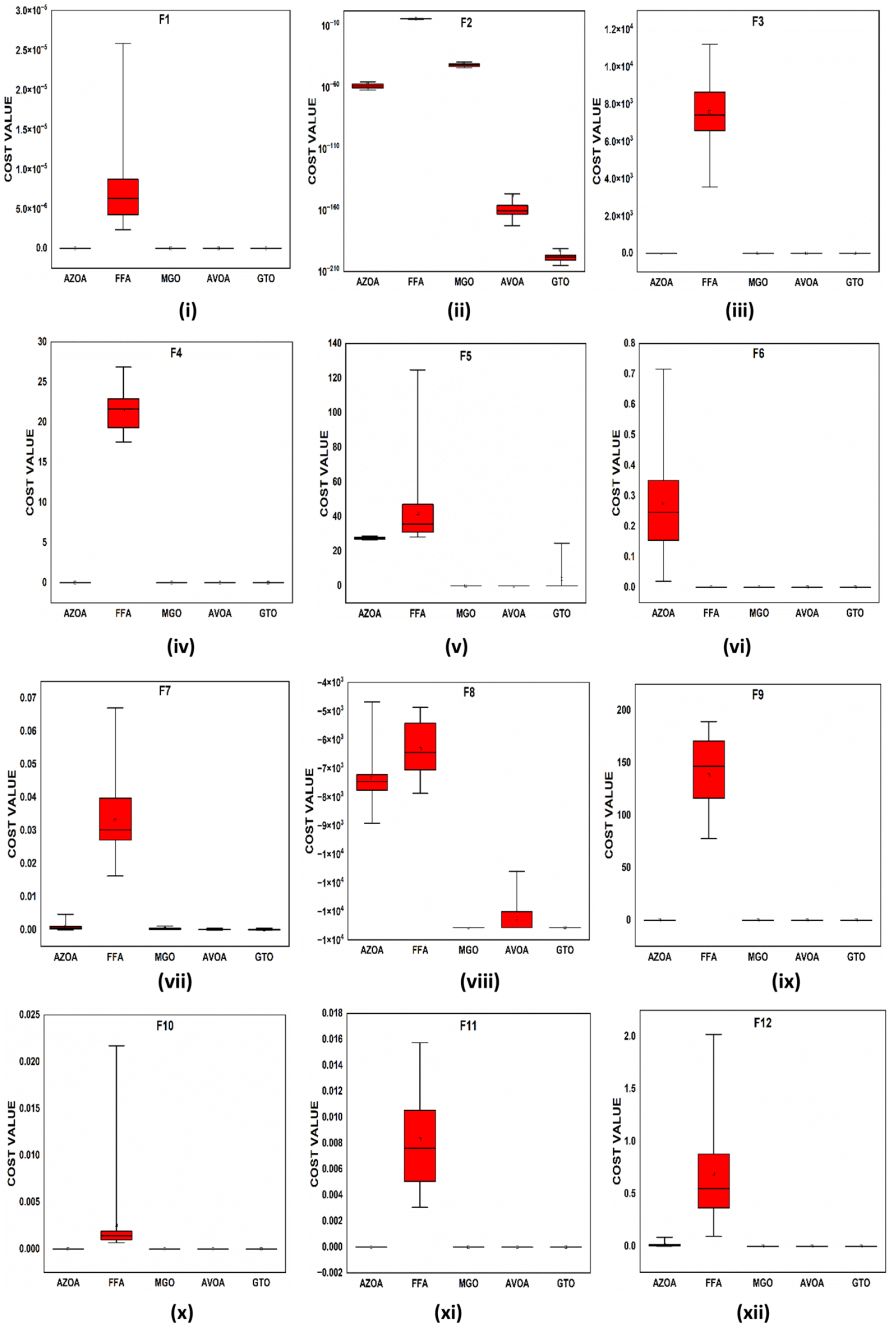

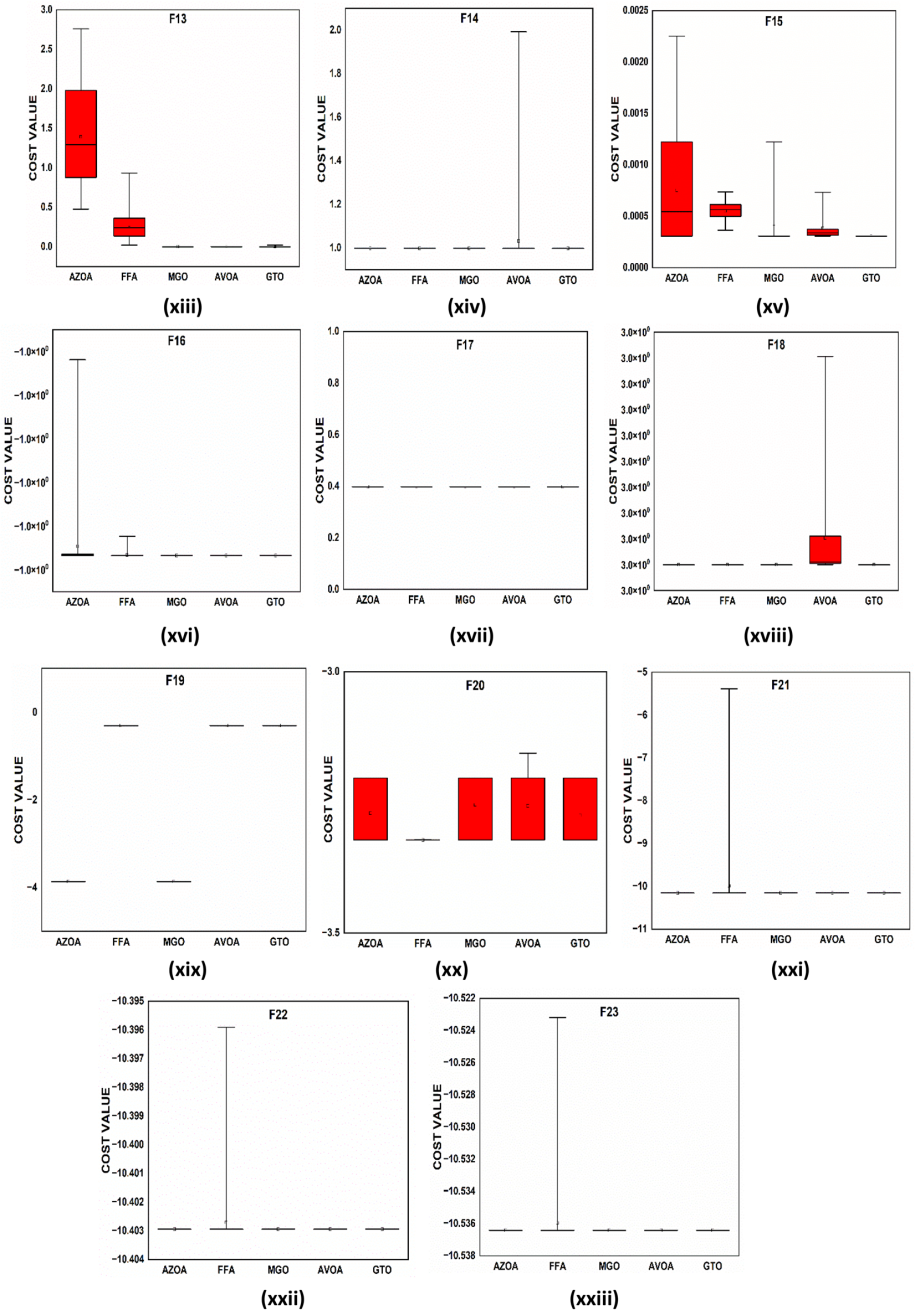
Box plots of AZOA and four latest outstanding metaheuristics in solving CEC-2005 benchmark functions.

The simulation outcomes of the CEC-2005 benchmark functions are presented in Tables 11 and 12. According to the simulation results, the proposed AZOA method is the third best optimizer compared to the four latest outstanding algorithms in solving $\mathrm{F}1{-}\mathrm{F}4$,$\mathrm{F}7$, $\mathrm{F}9{-}\mathrm{F}11$, $\mathrm{F}14{-}\mathrm{F}19$ and $\mathrm{F}21{-}\mathrm{F}23$ functions. The convergence curves of AZOA and the four latest outstanding algorithms while accomplishing the solution during algorithm iterations are depicted in Fig. 13. The simulation results revealed that the proposed method, namely AZOA with high exploitation, exploration, and balancing capabilities, had superior performance when compared with FFA and MGO and comparable performance with AVOA and GTO. Also, the outcomes of the Wilcoxon sum rank statistical test divulge the significant statistical superiority of AZOA against the two latest outstanding algorithms, namely FFA and MGO and AZOA. The boxplots of the performance of AZOA and competitor algorithms in solving the CEC-2005 benchmark set functions are displayed in Fig. 14. Analysis of the boxplot results demonstrate that the proposed AZOA method, in dealing with $\mathrm{F}1{-}\mathrm{F}4$, $\mathrm{F}7$, $\mathrm{F}9{-}\mathrm{F}11$, $\mathrm{F}14{-}\mathrm{F}19$ and $\mathrm{F}21{-}\mathrm{F}23$ functions, is the third best optimizer compared to rival algorithms.

**Table 11:** Statistical outcomes of AZOA and latest algorithms in solving CEC-2005 benchmark functions.

Function	AZOA	FFA	MGO	AVOA	GTO
	$avg (std)$	$avg (std)$ $t$-$values$	$avg (std)$ $t$-$values$	$avg (std)$ $t$-$values$	$avg (std)$ $t$-$values$
$\mathrm{F}1$	1.160E−108 (3.340E−108)	6.930E−06 (4.428E−06) **(−8.570E+00)**	8.940E−72 (4.524E−71) *(−1.080E*+*00)*	3.990E−293 (0) (1.900E+00)	0.000E+00 (0) (1.900E+00)
$\mathrm{F}2$	2.310E−57 (6.631E−57)	5.270E−05 (2.294E−05) **(−1.260E+01)**	2.060E−41 (6.543E−41) **(−1.730E+00)**	5.610E−149 (3.066E−148) (1.910E+00)	1.880E−193 (0) (1.910E+00)
$\mathrm{F}3$	4.560E−74 (2.459E−73)	7.560E+03 (1.648E+03) **(−2.510E+01)**	2.730E−08 (1.030E−07) *(−1.450E*+*00)*	1.540E−212 (0) *(1.010E*+*00)*	0.000E+00 (0) *(1.010E*+*00)*
$\mathrm{F}4$	4.690E−48 (1.345E−47)	2.150E+01 (2.683E+00) **(−4.390E+01)**	6.570E−23 (3.386E−22) *(−1.060E*+*00)*	5.620E−148 (2.973E−147) (1.910E+00)	1.110E−188 (0) (1.910E+00)
$\mathrm{F}5$	2.760E+01 (6.144E−01)	4.180E+01 (1.913E+01) **(−4.070E+00)**	2.010E−22 (9.317E−22) (2.460E+02)	4.900E−05 (4.899E−05) (2.460E+02)	3.950E+00 (8.982E+00) (1.440E+01)
$\mathrm{F}6$	2.780E−01 (1.794E−01)	6.920E−06 (3.797E−06) (8.480E+00)	3.890E−08 (1.959E−07) (8.480E+00)	4.980E−07 (3.597E−07) (8.480E+00)	2.300E−07 (4.831E−07) (8.480E+00)
$\mathrm{F}7$	2.690E−04 (9.923E−04)	3.310E−02 (1.152E−02) **(−1.520E+01)**	3.610E−04 (2.796E−04) (3.230E+00)	1.560E−04 (1.088E−04) (4.460E+00)	9.810E−05 (1.032E−04) (4.780E+00)
$\mathrm{F}8$	−7.340E+03 (8.628E+02)	−6.320E+03 (9.315E+02) **(−4.410E+00)**	−1.260E+04 (5.561E−08) (3.320E+01)	−1.230E+04 (5.198E+02) (2.670E+01)	−1.260E+04 (1.354E−04) (3.320E+01)
$\mathrm{F}9$	0.000E+00 (0)	1.380E+02 (3.370E+01) **(−2.250E+01)**	0.000E+00 (0) *(1.430E*+*00)*	0.000E+00 (0) *(−1.300E*+*00)*	0.000E+00 (0) *(−1.650*+*00)*
$\mathrm{F}10$	8.880E−16 (1.002E−31)	2.540E−03 (4.002E−03) **(−3.470E+00)**	1.24E−15 (1.084E−15) **(−1.800E+00)**	8.880E−16 (1.002E−31) *(0.000E*+*00)*	8.880E−16 (1.002E−31) *(0.000E*+*00)*
$\mathrm{F}11$	0.000E+00 (0)	8.310E−03 (3.773E−03) **(−1.210E+01)**	0.000E+00 (0) (−1.310E+00)	0.000E+00 (0) *(1.410E*+*00)*	0.000E+00 (0) *(−1.510E*+*00)*
$\mathrm{F}12$	1.190E−02 (1.719E−02)	6.890E−01 (4.667E−01) **(−7.940E+00)**	3.160E−26 (1.266E−25) (3.800E+00)	2.470E−08 (2.021E−08) (3.800E+00)	4.270E−08 (7.200E−08) (3.800E+00)
$\mathrm{F}13$	1.390E+00 (5.848E−01)	2.740E−01 (1.956E−01) (9.93E+00)	1.650E−32 (3.742E−33) (1.300E+01)	4.840E−08 (4.892E−08) (1.310E+01)	2.530E−03 (0) (1.320E+01)
$\mathrm{F}14$	9.980E−01 (3.387E−16)	9.980E−01 (3.387E−16) (*0.000E*+*00*)	9.980E−01 (3.387E−16) (*0.000E*+*00*)	1.030E+00 (1.815E−01) (*−1.000E*+*00*)	9.980E−01 (3.387E−16) (*0.000E*+*00*)
$\mathrm{F}15$	3.510E−04 (5.314E−04)	5.520E−04 (9.927E−05) (2.010E+00)	3.990E−04 (2.794E−04) (3.210E+00)	3.830E−04 (1.096E−04) (3.720E+00)	3.070E−04 (9.802E−19) (4.570E+00)
$\mathrm{F}16$	−1.030E+00 (1.649E−08)	−1.030E+00 (1.611E−09) (*1.320E*+*00*)	−1.030E+00 (0) *(1.430E*+*00)*	−1.030E+00 (0) *(1.430E*+*00)*	−1.030E+00 (0) *(1.430E*+*00)*
$\mathrm{F}17$	3.980E−01 (1.129E−16)	3.980E−01 (3.787E−16) *(−7.690E−01*)	3.980E−01 (1.129E−16) *(0.000E*+*00)*	3.980E−01 (1.129E−16) *(0.000E*+*00)*	3.980E−01 (1.129E−16) *(0.000E*+*00)*
$\mathrm{F}18$	3.000E+00 (1.322E−14)	3.000E+00 (5.718E−15) (2.03E+00)	3.000E+00 (4.801E−15) (2.590E+00)	3.00E+00 (3.746E−06) **(−3.010E+00)**	3.000E+00 (2.565E−15) (3.610E+00)
$\mathrm{F}19$	−3.860E+00 (3.498E−15)	−3.000E−01 (0) **(−5.570E+15)**	−3.860E+00 (2.710E−15) *(1.100E*+*00)*	−3.000E−01 (0) **(−5.58E+15)**	−3.000E−01 (0) **(−5.580E+15)**
$\mathrm{F}20$	−3.270E+00 (5.992E−02)	−3.320E+00 (7.462E−05) (4.710E+00)	−3.250E+00 (5.992E−02) *(−1.002E*+*00)*	−3.260E+00 (6.271E−02) *(8.710E−01)*	−3.270E+00 (5.924E−02) *(2.580E−01)*
$\mathrm{F}21$	−1.020E+01 (1.806E−15)	−9.990E+00 (8.70E−01) *(−1.040E*+*00)*	−1.020E+01 (1.806E−15) *(0.000E*+*00)*	−1.020E+01 (4.544E−13) **(−2.800E+00)**	−1.020E+01 (1.806E−15) *(0.000E*+*00)*
$\mathrm{F}22$	−1.040E+01 (0)	−1.040E+01 (1.28E−03) *(−1.000E*+*00)*	−1.040E+01 (0) **(−4.320+00)**	−1.040E+01 (6.310E−13) **(−2.940E+00)**	−1.040E+01 (0*)* ***(−5.460*+*00)***
$\mathrm{F}23$	−1.050E+01 (9.033E−15)	−1.050E+01 (2.41E−03) *(−1.030E*+*00)*	−1.050E+01 (9.033E−15) *(0.000E*+*00)*	−1.050E+01 (3.830E−13) **(−2.180E+00)**	−1.050E+01 (9.033E−15) *(0.000E*+*00)*
$w/t/l$		12/6/5	3/12/8	5/8/10	2/10/11

Significant values are in bold/italic.

**Table 12:** $p$-values by Wilcoxon Rank Sum test with five percent significance level for $\mathrm{F}1{-}\mathrm{F}23$.

Function	FFT versus AZOA		MGO versus AZOA		AVOA versus AZOA		GTO versus AZOA	
	$p$ $\mathrm{values}$	$H$	$p$ $\mathrm{values}$	$H$	$p$ $\mathrm{values}$	$H$	$p$ $\mathrm{values}$	$H$
$\mathrm{F}1$	1.779E−06	1	1.779E−06	1	1.779E−06	1	1.779E−06	1
$\mathrm{F}2$	1.778E−06	1	1.778E−06	1	1.779E−06	1	1.779E−06	1
$\mathrm{F}3$	1.779E−06	1	1.779E−06	1	1.779E−06	1	1.779E−06	1
$\mathrm{F}4$	1.779E−06	1	1.779E−06	1	1.779E−06	1	1.779E−06	1
$\mathrm{F}5$	1.779E−06	1	1.779E−06	1	1.779E−06	1	1.779E−06	1
$\mathrm{F}6$	1.779E−06	1	1.779E−06	1	1.779E−06	1	1.779E−06	1
$\mathrm{F}7$	1.779E−06	1	4.990E−04	1	1.840E−05	1	2.413E−06	1
$\mathrm{F}8$	3.660E−04	1	1.779E−06	1	1.779E−06	1	1.779E−06	1
$\mathrm{F}9$	1.779E−06	1	NA	0	NA	0	NA	0
$\mathrm{F}10$	1.779E−06	1	NA	0	NA	0	NA	0
$\mathrm{F}11$	1.779E−06	1	NA	0	NA	0	NA	0
$\mathrm{F}12$	1.779E−06	1	1.779E−06	1	1.779E−06	1	1.779E−06	1
$\mathrm{F}13$	2.413E−06	1	1.779E−06	1	1.779E−06	1	1.779E−06	1
$\mathrm{F}14$	NA	0	NA	0	NA	0	NA	0
$\mathrm{F}15$	1.670E−01	0	3.090E−03	1	1.880E−02	1	1.220E−04	1
$\mathrm{F}16$	1.090E−01	0	1.560E−02	1	1.560E−02	1	1.560E−02	1
$\mathrm{F}17$	NA	0	NA	0	NA	0	NA	0
$\mathrm{F}18$	NA	0	NA	0	NA	0	NA	0
$\mathrm{F}19$	4.466E−08	1	NA	0	4.466E−08	1	4.466E−08	1
$\mathrm{F}20$	1.030E−01	0	3.580E−01	0	4.690E−02	1	8.190E−01	0
$\mathrm{F}21$	1.950E−03	1	NA	0	NA	0	NA	0
$\mathrm{F}22$	9.770E−04	1	NA	0	NA	0	NA	0
$\mathrm{F}23$	3.130E−02	1	NA	0	NA	0	NA	0

**Figure 15:**
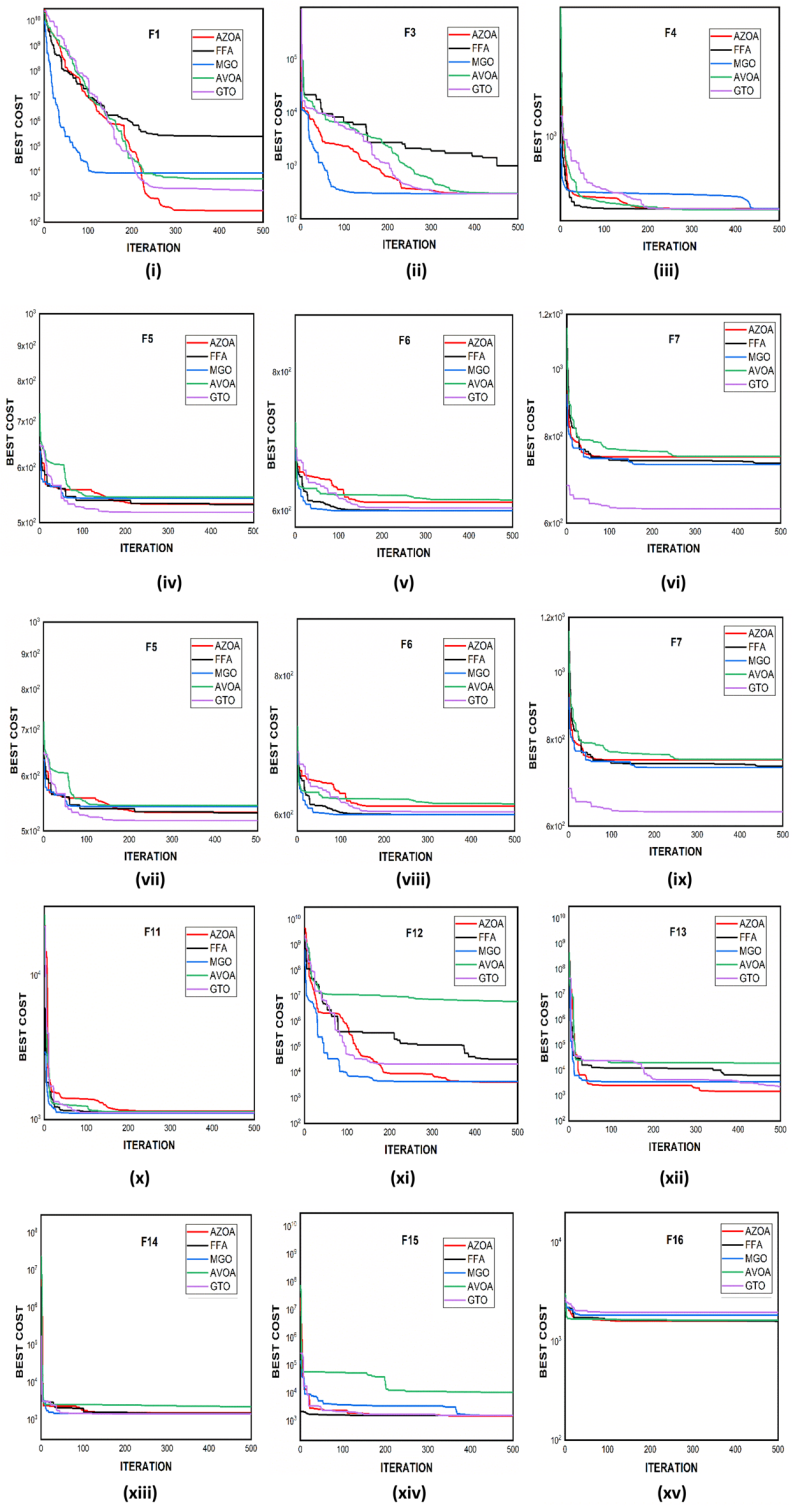

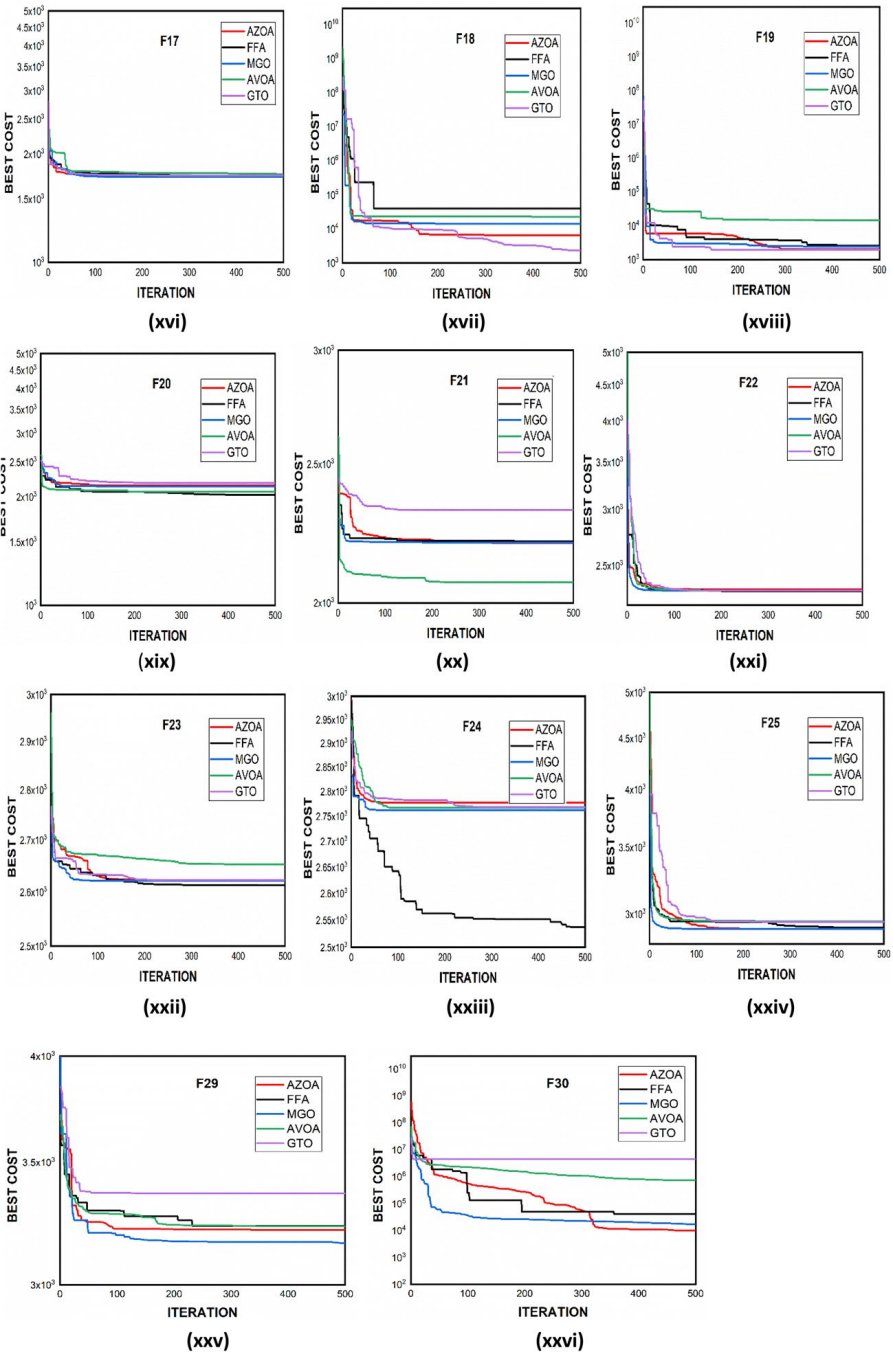
Convergence graph of AZOA and four latest outstanding metaheuristics in solving CEC-2017 benchmark functions.

**Figure 16:**
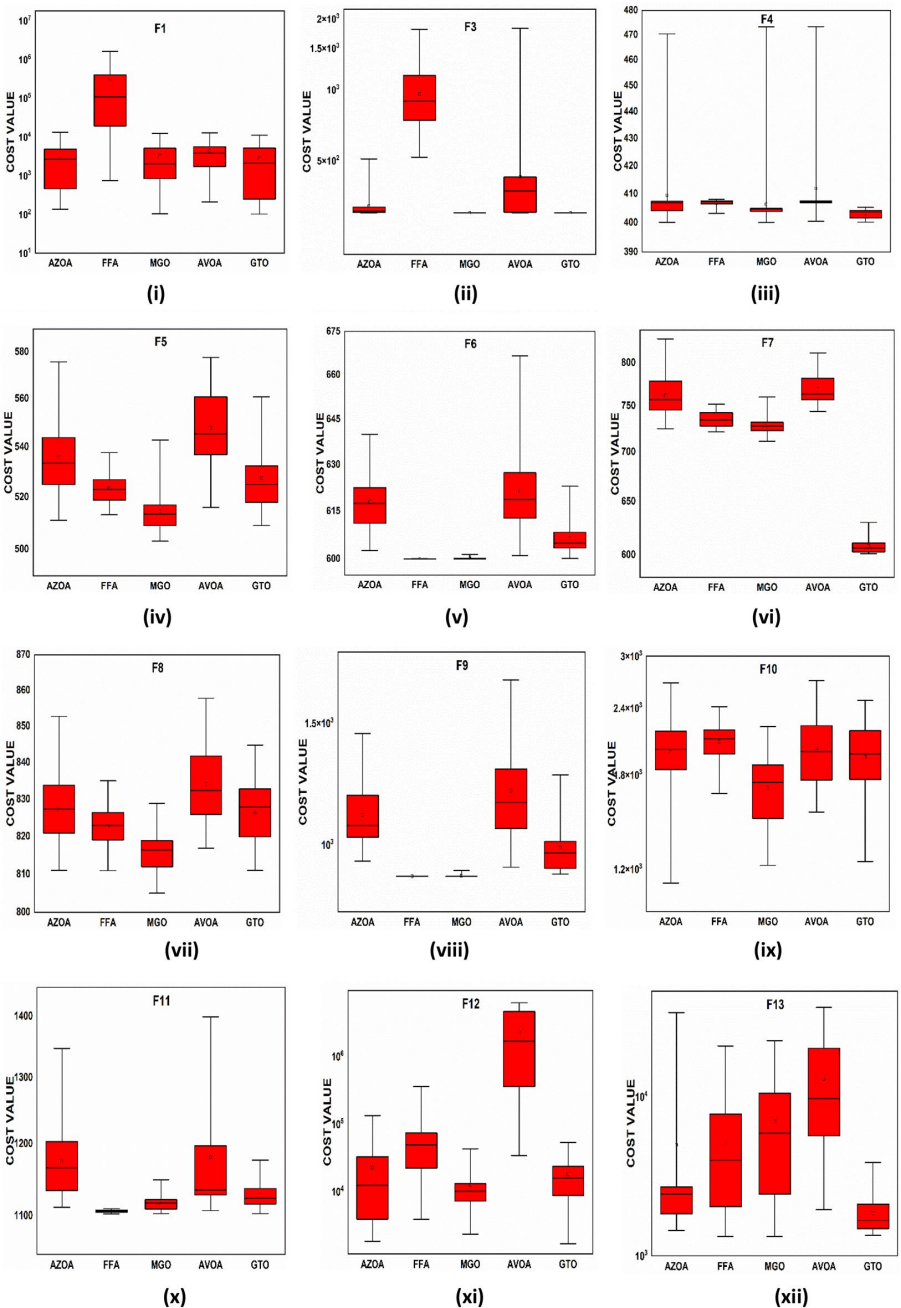

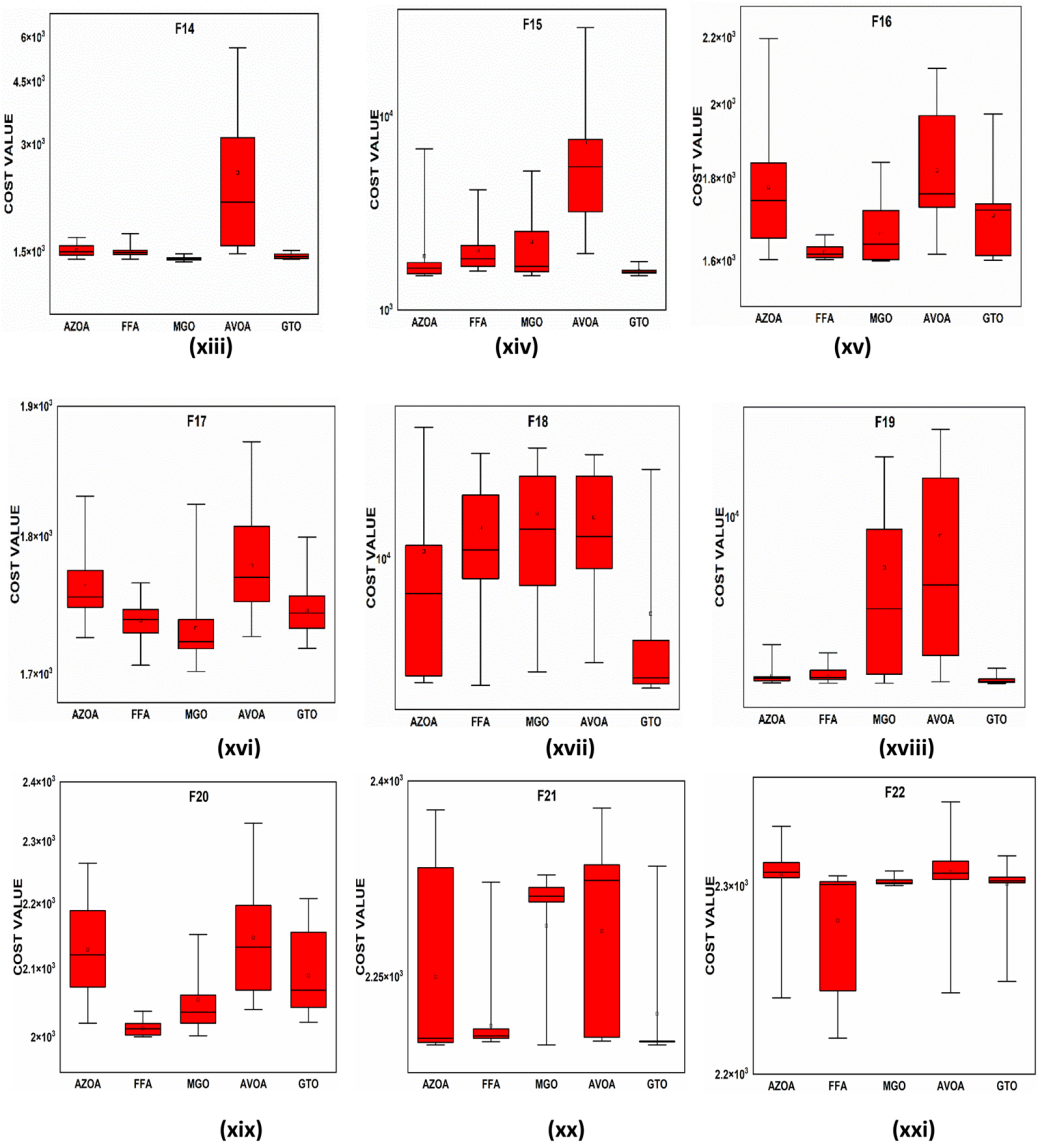

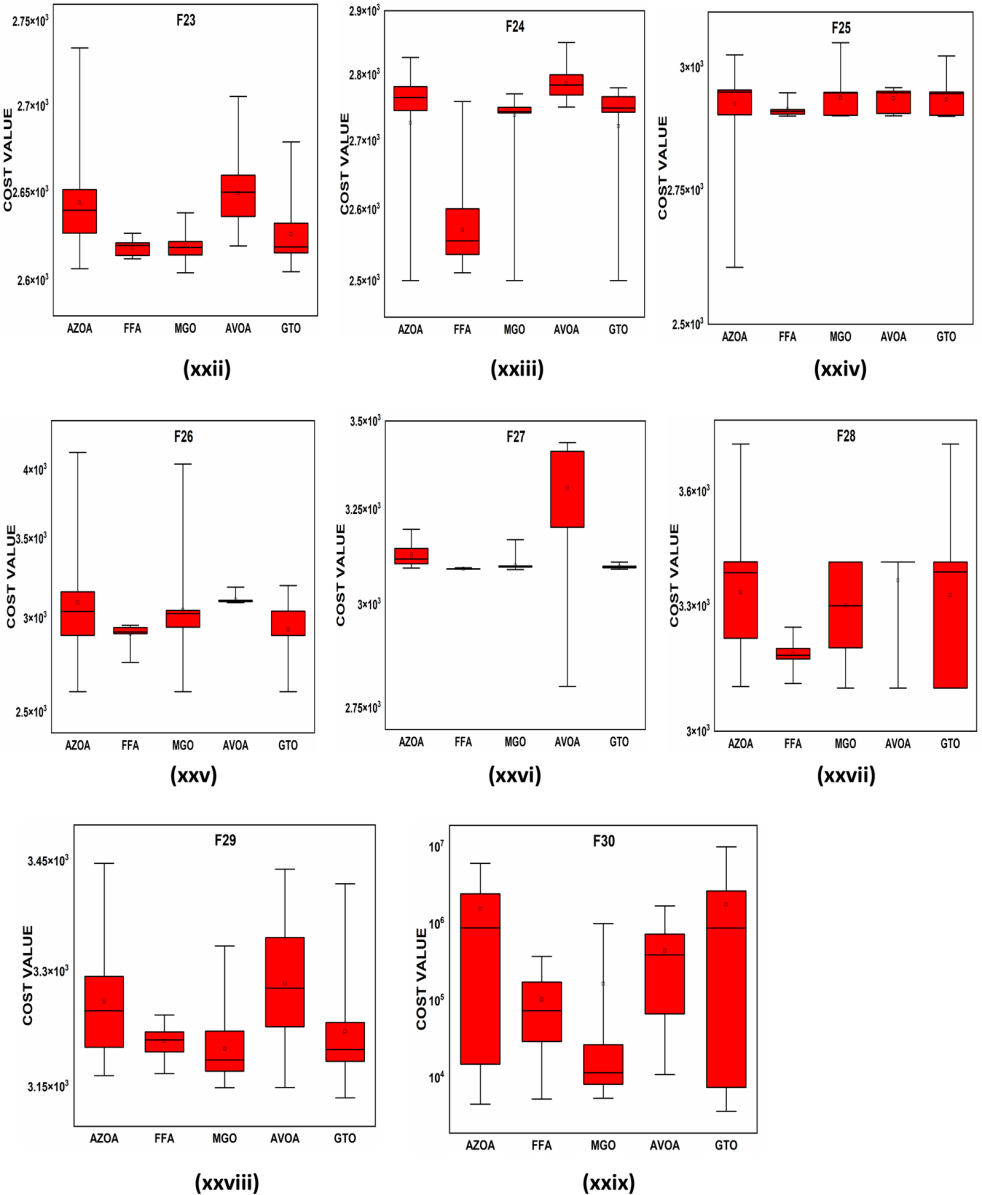
Box plots of AZOA and four latest outstanding metaheuristics in solving CEC-2017 benchmark functions.

The statistical outcomes of the CEC-2017 benchmark functions employing AZOA and the four latest outstanding algorithms are presented in Tables 13 and 14. What is concluded from the simulation results is that the proposed AZOA method provided better result when it compared with AVOA for $\mathrm{F}1$, $\mathrm{F}3$, $\mathrm{F}5{-}\mathrm{F}9$, $\mathrm{F}11$, $\mathrm{F}14{-}\mathrm{F}17$, and $\mathrm{F}19{-}\mathrm{F}29$ functions and offer equivalent result compared with FFA and MGO. The convergence curves of AZOA and the four latest outstanding algorithms while achieving the solution for the CEC-2005 functions during algorithm iterations are presented in Fig. 15. The analysis of the simulation results shows that the proposed AZOA method has provided better performance for functions $\mathrm{F}1$, $\mathrm{F}13$, and $\mathrm{F}30$ and comparable performance for other functions. The boxplots of the performance of AZOA and competitor algorithms in solving the CEC-2017 benchmark set functions are shown in Fig. 16.

**Table 13:** Statistical outcomes of AZOA and latest algorithms in solving CEC-2017 benchmark function.

Function	AZOA	FFA	MGO	AVOA	GTO
	$avg (std)$	$avg (std)$ $t$-$values$	$avg (std)$ $t$-$values$	$avg (std)$ $t$-$values$	$avg (std)$ $t$-$values$
$\mathrm{F}1$	3.080E+03 (3.310E+03)	3.230E+05(4.330E+05) **(−4.040E+00)**	3.270E+03 (3.230E+03) *(−2.270E−01)*	4.370E+03 (3.430E+03) *(−1.490E*+*00)*	2.870E+03 (2.850E+03) *(2.580E−01)*
$\mathrm{F}3$	3.000E+02 (4.340E+01)	9.590E+02 (3.080E+02) **(−1.120E+01)**	3.000E+02 (5.960E−07) (2.560E+00)	4.260E+02 (2.810E+02) **(−2.040E+00)**	3.000E+02 (6.260E−04) (2.560E+00)
$\mathrm{F}4$	4.100E+02 (1.600E+01)	4.070E+02 (1.320E+00) *(9.900E−01)*	4.060E+02 (1.280E+01) *(8.830E−01)*	4.120E+02 (1.770E+01) *(−5.350E−01)*	4.030E+02 (1.600E+00) (2.210E+00)
$\mathrm{F}5$	5.360E+02 (1.430E+01)	5.230E+02 (6.390E+00) (4.380E+00)	5.140E+02 (8.330E+00) (7.130E+00)	5.480E+02 (1.680E+01) **(−2.950E+00)**	5.270E+02 (1.260E+01) (2.460E+00)
$\mathrm{F}6$	6.180E+02 (8.5800E+00)	6.000E+02 (1.750E−02) (1.150E+01)	6.000E+02 (3.180E−01) (1.140E+01)	6.210E+02 (1.350E+01) *(−1.120E*+*00)*	6.070E+02 (6.480E+00) (5.490E+00)
$\mathrm{F}7$	7.620E+02 (2.400E+01)	7.350E+02 (8.300E+00) (5.790E+00)	7.280E+02 (9.150E+00) (7.280E+00)	7.690E+02 (1.810E+01) *(−1.310E*+*00)*	6.080E+02 (7.260E+00) (3.360E+01)
$\mathrm{F}8$	8.270E+02 (9.300E+00)	8.230E+02 (5.960E+00) (2.190E+00)	8.160E+02 (6.120E+00) (5.360E+00)	8.340E+02 (1.070E+01) **(−2.650E+00)**	8.260E+02 (8.950E+00) *(4.010E−01)*
$\mathrm{F}9$	1.100E+03 (1.200E+02)	9.000E+02 (2.640E−01) (9.310E+00)	9.020E+02 (4.860E+00) (9.230E+00)	1.200E+03 (2.050E+02) **(−2.180E+00)**	9.900E+02 (8.670E+01) (4.200E+00)
$\mathrm{F}10$	1.990E+03 (3.510E+02)	2.080E+03 (1.700E+02) *(−1.250E*+*00)*	1.700E+03 (2.790E+02) (3.450E+00)	2.020E+03 (3.110E+02) *(−3.680E−01)*	1.950E+03 (3.080E+02) *(4.790E−01)*
$\mathrm{F}11$	1.180E+03 (5.460E+01)	1.110E+03 (2.010E+00) (6.940E+00)	1.120E+03 (9.360E+00) (5.800E+00)	1.180E+03 (8.660E+01) *(−2.960E−01)*	1.130E+03 (1.770E+01) (4.450E+00)
$\mathrm{F}12$	2.270E+04 (2.830E+04)	7.590E+04 (8.390E+04) **(−3.290E+00)**	1.250E+04 (9.480E+03) (1.870E+00)	2.250E+06 (2.160E+06) **(−5.640E+00)**	1.800E+04 (1.350E+04) *(8.100E−01)*
$\mathrm{F}13$	4.960E+03 (8.070E+03)	5.150E+03 (4.010E+03) *(−1.160E−01)*	7.000E+03 (5.330E+03) *(−1.160E*+*00)*	1.290E+04 (9.020E+03) **(−3.580E+00)**	1.850E+03 (5.390E+02) (2.110E+00)
$F14$	1.510E+03 (5.640E+01)	1.500E+03 (4.680E+01) *(7.640E−01)*	1.430E+03 (1.710E+01) (7.250E+00)	2.500E+03 (1.260E+03) **(−4.290E+00)**	1.460E+03 (2.600E+01) (4.480E+00)
$\mathrm{F}15$	1.900E+03 (1.030E+03)	2.030E+03 (5.450E+02) *(−6.190E−01)*	2.250E+03 (1.100E+03) *(−1.290E*+*00)*	7.350E+03 (6.440E+03) **(−4.580E+00)**	1.590E+03 (6.500E+01) *(1.620E*+*00)*
$\mathrm{F}16$	1.780E+03 (1.450E+02)	1.620E+03 (1.570E+01) (5.870E+00)	1.660E+03 (7.610E+01) (3.810E+00)	1.820E+03 (1.430E+02) *(−1.150E*+*00)*	1.710E+03 (1.080E+02) (2.120E+00)
$\mathrm{F}17$	1.760E+03 (2.540E+01)	1.740E+03 (1.360E+01) (4.680E+00)	1.730E+03 (2.990E+01) (4.220E+00)	1.780E+03 (3.820E+01) **(−1.820E+00)**	1.750E+03 (1.830E+01) (3.050E+00)
$\mathrm{F}18$	1.100E+04 (1.340E+04)	1.500E+04 (1.050E+04) *(−1.300E*+*00)*	1.800E+04 (1.210E+04) **(−2.140E+00)**	1.710E+04 (1.190E+04) **(−1.880E+00)**	4.880E+03 (6.960E+03) (2.220E+00)
$\mathrm{F}19$	2.050E+03 (2.000E+02)	2.100E+03 (1.800E+02) *(−9.080E−01)*	6.050E+03 (4.700E+03) **(−4.650E+00)**	8.290E+03 (6.880E+03) **(−4.970E+00)**	1.970E+03 (6.590E+01) (2.120E+00)
$\mathrm{F}20$	2.130E+03 (7.060E+01)	2.010E+03 (9.370E+00) (8.960E+00)	2.050E+03 (5.070E+01) (4.710E+00)	2.150E+03 (8.110E+01) *(−9.300E−01)*	2.090E+03 (6.110E+01) (2.300E+00)
$\mathrm{F}21$	2.250E+03 (6.810E+01)	2.210E+03 (2.410E+01) (2.730E+00)	2.290E+03 (4.860E+01) **(−2.510E+00)**	2.280E+03 (6.610E+01) **(−1.980E+00)**	2.220E+03 (4.690E+01) (1.800E+00)
$\mathrm{F}22$	2.310E+03 (1.490E+01)	2.280E+03 (3.220E+01) (3.930E+00)	2.300E+03 (1.640E+00) *(1.630E*+*00)*	2.310E+03 (1.510E+01) *(−2.300E−01)*	2.300E+03 (1.170E+01) *(1.620E*+*00)*
$\mathrm{F}23$	2.640E+03 (2.720E+01)	2.620E+03 (4.410E+00) (5.100E+00)	2.620E+03 (7.520E+00) (4.840E+00)	2.650E+03 (1.940E+01) *(−8.890E−01)*	2.630E+03 (1.740E+01) (3.040E+00)
$\mathrm{F}24$	2.730E+03 (1.050E+02)	2.570E+03 (5.380E+01) (7.270E+00)	2.740E+03 (4.570E+01) *(−5.600E−01)*	2.790E+03 (2.230E+01) **(−3.060E+00)**	2.720E+03 (8.910E+01) *(2.110E−01)*
$\mathrm{F}25$	2.920E+03 (6.730E+01)	2.910E+03 (1.470E+01) *(9.260E−01)*	2.940E+03 (3.120E+01) *(−9.250E−01)*	2.930E+03 (2.230E+01) *(−8.580E−01)*	2.930E+03 (2.890E+01) *(−6.460E−01)*
$\mathrm{F}26$	3.090E+03 (3.240E+02)	2.910E+03 (5.190E+01) (3.020E+00)	3.050E+03 (2.620E+02) *(5.830E−01)*	3.110E+03 (2.020E+01) *(−2.650E−01)*	2.930E+03 (1.380E+02) (2.460E+00)
$\mathrm{F}27$	3.130E+03 (3.080E+01)	3.090E+03 (9.670E−01) (6.430E+00)	3.100E+03 (1.340E+01) (4.540E+00)	3.310E+03 (1.530E+02) **(−6.380E+00)**	3.100E+03 (4.240E+00) (5.480E+00)
$\mathrm{F}28$	3.330E+03 (1.490E+02)	3.180E+03 (2.670E+01) (5.520E+00)	3.300E+03 (1.140E+02) *(9.750E−01)*	3.360E+03 (9.980E+01) *(−9.140E−01)*	3.330E+03 (2.080E+02) *(1.630E−01)*
$\mathrm{F}29$	3.260E+03 (6.880E+01)	3.210E+03 (2.050E+01) (3.900E+00)	3.200E+03 (4.690E+01) (4.010E+00)	3.280E+03 (8.260E+01) *(−1.220E*+*00)*	3.220E+03 (6.930E+01) (2.170E+00)
$\mathrm{F}30$	1.560E+06 (1.730E+06)	1.030E+05 (9.370E+04) (4.630E+00)	1.660E+05 (3.290E+05) (4.360E+00)	4.490E+05 (4.190E+05) (3.440E+00)	1.780E+06 (2.230E+06) *(−4.250E−01)*
$w/t/l$		3/7/20	3/9/11	14/8/8	0/10/20

Significant values are in bold/italic.

**Table 14:** $p$ values by Wilcoxon Rank Sum test with five percent significance level for $\mathrm{F}1{-}\mathrm{F}23$.

Function	FFT versus AZOA		MGO versus AZOA		AVOA versus AZOA		GTO versus AZOA	
	*p* values	$H$	*p* values	$H$	*p* values	$H$	*p* values	$H$
$\mathrm{F}1$	3.260E−06	1	8.650E−01	0	8.310E−02	0	8.650E−01	0
$\mathrm{F}3$	1.780E−06	1	1.780E−06	1	4.460E−03	1	1.780E−06	1
$\mathrm{F}4$	2.920E−01	0	1.780E−02	1	2.310E−01	0	7.860E−04	1
$\mathrm{F}5$	2.460E−04	1	3.600E−06	1	1.500E−02	0	1.190E−02	1
$\mathrm{F}6$	1.780E−06	1	1.780E−06	1	2.560E−01	0	3.810E−05	1
$\mathrm{F}7$	1.840E−05	1	2.950E−06	1	6.950E−02	0	1.780E−06	1
$\mathrm{F}8$	5.260E−02	0	1.270E−05	1	7.850E−03	1	5.750E−01	0
$\mathrm{F}9$	1.780E−06	1	1.780E−06	1	5.780E−02	0	4.620E−04	1
$\mathrm{F}10$	2.230E−01	0	3.010E−03	1	9.300E−01	0	7.380E−01	0
$\mathrm{F}11$	1.780E−06	1	2.410E−06	1	9.790E−01	0	7.680E−05	1
$\mathrm{F}12$	9.800E−04	1	2.470E−01	0	1.780E−06	1	9.470E−01	0
$\mathrm{F}13$	1.540E−01	0	1.340E−02	1	3.380E−04	1	3.960E−04	1
$\mathrm{F}14$	3.960E−01	0	2.410E−06	1	5.890E−06	1	2.270E−04	1
$\mathrm{F}15$	6.530E−03	1	7.270E−02	0	1.780E−06	1	2.340E−02	1
$\mathrm{F}16$	3.980E−06	1	1.050E−03	1	1.930E−01	0	5.010E−02	1
$\mathrm{F}17$	1.170E−04	1	6.770E−04	1	9.070E−02	0	4.180E−03	1
$\mathrm{F}18$	1.220E−01	0	1.980E−02	1	3.200E−02	1	9.410E−03	1
$\mathrm{F}19$	2.830E−01	0	4.970E−05	1	7.150E−06	1	1.340E−02	1
$\mathrm{F}20$	1.780E−06	1	3.660E−04	1	6.770E−01	0	4.330E−02	1
$\mathrm{F}21$	5.750E−01	0	7.270E−02	1	6.950E−02	0	9.410E−03	1
$\mathrm{F}22$	1.840E−05	1	5.820E−04	1	5.470E−01	0	9.990E−03	1
$\mathrm{F}23$	1.050E−05	1	9.920E−05	1	2.310E−01	0	1.190E−02	1
$\mathrm{F}24$	1.840E−05	1	1.030E−01	0	1.030E−01	0	6.340E−02	0
$\mathrm{F}25$	1.420E−02	1	6.770E−01	0	8.650E−01	0	6.770E−01	0
$\mathrm{F}26$	4.760E−03	1	7.230E−01	0	2.150E−01	0	3.370E−02	1
$\mathrm{F}27$	1.780E−06	1	2.910E−05	1	7.040E−05	1	7.880E−06	1
$\mathrm{F}28$	9.540E−06	1	4.190E−01	0	2.080E−01	0	9.550E−01	0
$\mathrm{F}29$	1.620E−03	1	4.280E−04	1	3.520E−01	0	4.330E−02	1
$\mathrm{F}30$	1.510E−04	1	4.620E−04	1	3.210E−03	1	9.220E−01	0

**Figure 17:**
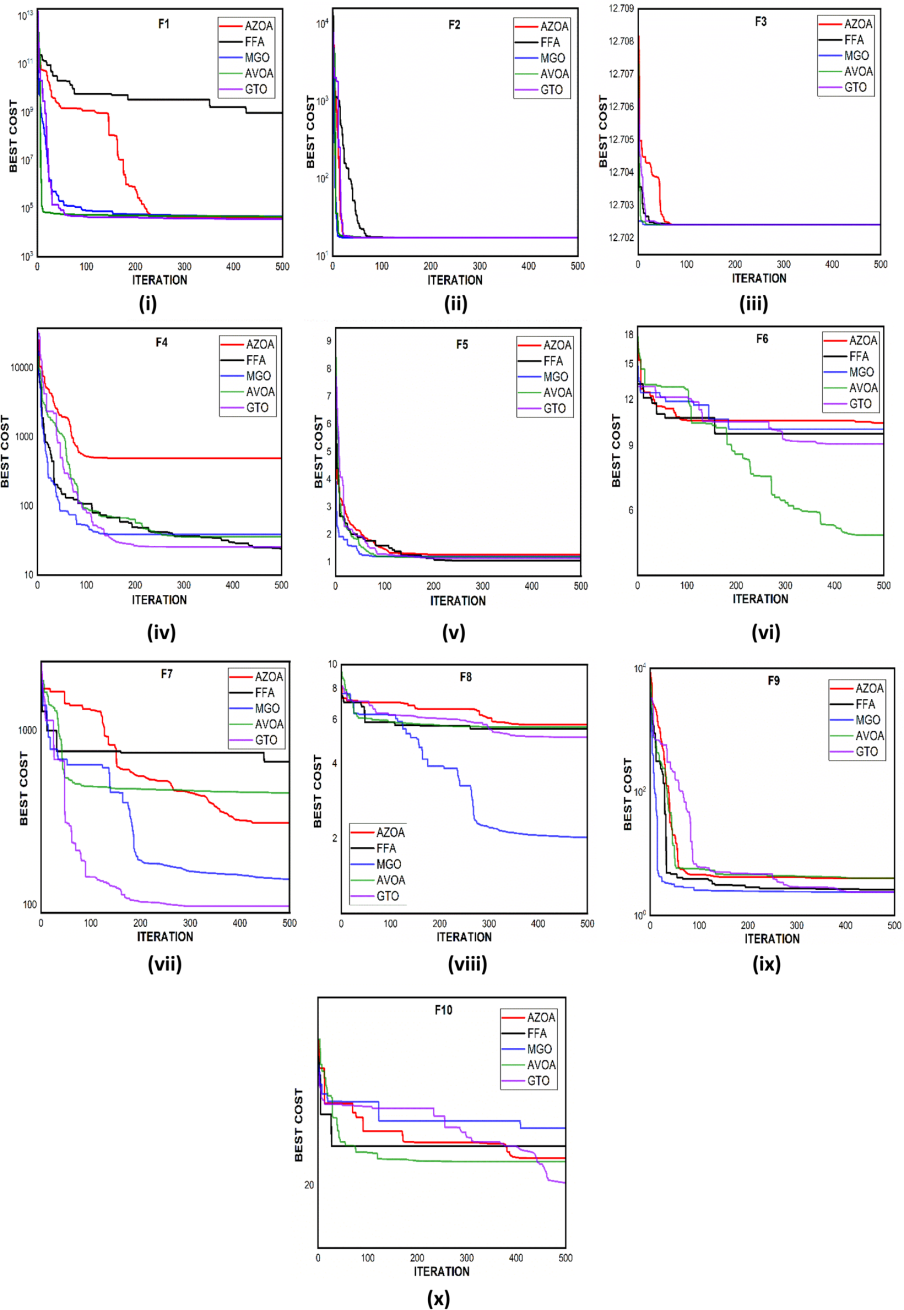
Convergence graph of AZOA and four latest outstanding metaheuristics in solving CEC-2019 benchmark functions.

**Figure 18:**
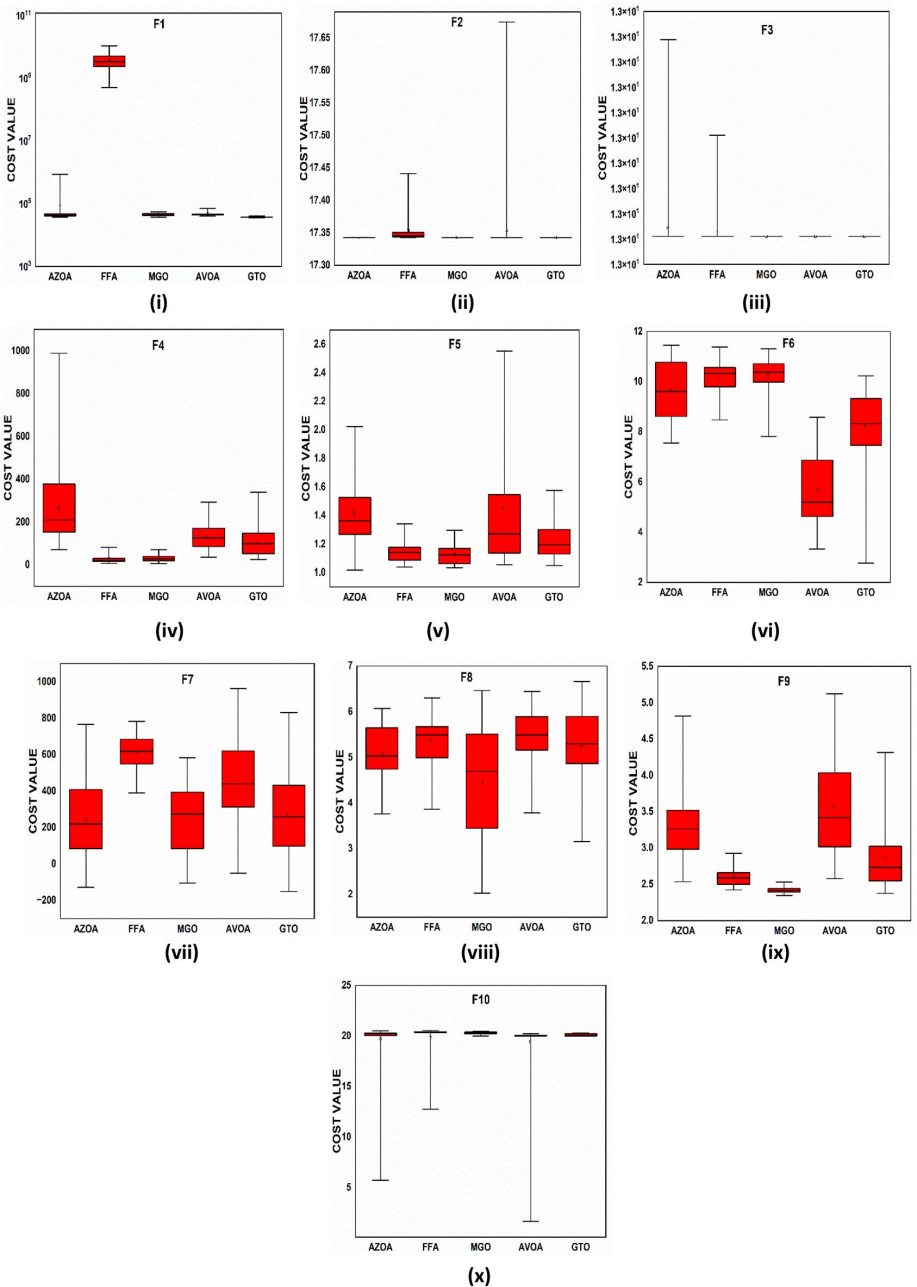
Box plots of AZOA and four latest outstanding metaheuristics in solving CEC-2019 benchmark functions.

The optimization outcomes of the CEC-2019 benchmark functions employing AZOA and the four latest outstanding algorithms are presented in Tables 15 and 16. Firstly, when AZOA is compared with FFA, it provides the best result for functions $\mathrm{F}2{-}\mathrm{F}4$, $\mathrm{F}6{-}\mathrm{F}8$, and $\mathrm{F}10$. Secondly, it provided a better result for functions $\mathrm{F}2,$ $\mathrm{F}3$, $\mathrm{F}6$, $\mathrm{F}7$, and $\mathrm{F}10$ compared with MGO. Thirdly, AZOA provides better outcomes compared with AVOA except for functions $\mathrm{F}1$, $\mathrm{F}4$, $\mathrm{F}6$, and $\mathrm{F}8$. Lastly, AZOA offers the best outcomes for functions $\mathrm{F}2$, $\mathrm{F}3$, $\mathrm{F}7$, $\mathrm{F}8$, and $\mathrm{F}10$. Hence, AZOA performs better as compared with the four latest outstanding algorithms. The convergent graph of the algorithms that have been implemented is shown in Fig. 17. It is clear from these curves that the AZOA performs comparable convergence for most of the functions. In Fig. 18, the box plot of the compared algorithms along with the proposed AZOA for solving the functions is presented as a box plot. From Fig. 18, the boxplot study indicates that the AZOA has a smaller width and a more efficient centre than competitor metaheuristic algorithms.

**Table 15:** Statistical outcomes of AZOA and latest algorithms in solving CEC-2019 benchmark function.

Function	AZOA	FFA	MGO	AVOA	GTO
	$avg (std)$	$avg (std)$ $t$-$values$	$avg (std)$ $t$-$values$	$avg (std)$ $t$-$values$	$avg (std)$ $t$-$values$
$\mathrm{F}1$	8.160E+04(1.550E+05)	4.090E+09 (2.740E+09) **(−8.160E+00)**	4.520E+04 (5.060E+03) *(1.280E*+*00)*	4.630E+04 (5.240E+03) *(1.240E*+*00)*	3.790E+04 (1.170E+03) *(1.540E*+*00)*
$\mathrm{F}2$	1.730E+01 (3.480E−05)	1.740E+01 (2.030E−02) **(−2.800E+00)**	1.730E+01 (0.000E+00) *(1.070E*+*00)*	1.740E+01 (6.050E−02) *(−9.990E−01)*	1.730E+01 (9.020E−14) *(1.070E*+*00)*
$\mathrm{F}3$	1.270E+01 (2.850E−06)	1.270E+01 (1.460E−06) *(6.200E−01)*	1.270E+01 (1.270E−09) *(1.210E*+*00)*	1.270E+01 (5.730E−14) *(1.210E*+*00)*	1.270E+01 (9.030E−15) *(1.210E*+*00)*
$\mathrm{F}4$	2.670E+02 (1.900E+02)	2.710E+01 (1.920E+01) (6.880E+00)	3.130E+01 (1.570E+01) (6.770E+00)	1.330E+02 (6.260E+01) (3.670E+00)	1.110E+02 (6.910E+01) (4.220E+00)
$\mathrm{F}5$	1.420E+00 (2.530E−01)	1.150E+00 (7.610E−02) (5.630E+00)	1.120E+00 (6.960E−02) (6.150E+00)	1.450E+00 (4.320E−01) *(−3.330E−01)*	1.220E+00 (1.330E−01) (3.740E+00)
$\mathrm{F}6$	9.670E+00 (1.140E+00)	1.020E+01 (6.340E−01) **(−2.340E+00)**	1.020E+01 (7.280E−01) **(−2.290E+00)**	5.640E+00 (1.520E+00) (1.160E+01)	8.270E+00 (1.460E+00) (4.140E+00)
$\mathrm{F}7$	2.450E+02 (2.090E+02)	6.120E+02 (9.180E+01) **(−8.800E+00)**	2.560E+02 (1.710E+02) *(−2.310E−01)*	4.470E+02 (2.520E+02) **(−3.380E+00)**	2.690E+02 (2.430E+02) *(−4.150E−01)*
$\mathrm{F}8$	5.060E+00 (6.100E−01)	5.350E+00 (5.230E−01) **(−1.950E+00)**	4.440E+00 (1.220E+00) (2.500E+00)	5.390E+00 (6.490E−01) **(−1.990E+00)**	5.220E+00 (7.530E−01) *(−9.050E−01)*
$\mathrm{F}9$	3.320E+00 (4.900E−01)	2.600E+00 (1.270E−01) (7.730E+00)	2.420E+00 (4.100E−02) (1.000E+01)	3.560E+00 (6.700E−01) *(−1.610E*+*00)*	2.850E+00 (4.380E−01) (3.910E+00)
$\mathrm{F}10$	1.970E+01 (2.650E+00)	1.980E+01 (1.800E+00) *(−1.760E−01)*	2.030E+01 (1.500E−01) *(−1.160E*+*00)*	1.940E+01 (3.360E+00) *(3.750E−01)*	2.010E+01 (1.170E−01) *(−7.880E−01)*
$w/t/l$		5/1/4	1/5/4	2/6/2	0/6/4

Significant values are in bold/italic.

**Table 16:** $p$-values by Wilcoxon Rank Sum test with five percent significance level for $\mathrm{F}1{-}\mathrm{F}10$.

Function	FFT versus AZOA		MGO versus AZOA		AVOA versus AZOA		GTO versus AZOA	
	$p$ $\mathrm{values}$	$H$	$p$ $\mathrm{values}$	$H$	$p$ $\mathrm{values}$	$H$	$p$ $\mathrm{values}$	$H$
$\mathrm{F}1$	1.7793E−06	1	9.140E−01	0	4.497E−01	0	5.893E−06	1
$\mathrm{F}2$	3.906E−03	1	NA	0	1	0	NA	0
$\mathrm{F}3$	NA	0	NA	0	NA	0	NA	0
$\mathrm{F}4$	1.779E−06	1	1.7782E−06	1	4.620E−04	1	7.042E−05	1
$\mathrm{F}5$	9.531E−06	1	6.4802E−06	1	6.252E−01	0	2.337E−03	1
$\mathrm{F}6$	2.857E−02	1	4.374E−02	1	1.779E−06	1	7.001E−04	1
$\mathrm{F}7$	4.846E−06	1	7.074E−01	0	8.462E−04	1	7.227E−01	0
$\mathrm{F}8$	1.730E−01	0	4.436E−02	1	4.33E−02	1	4.876E−01	0
$\mathrm{F}9$	1.965E−06	1	1.777E−06	1	1.371E−01	0	2.460E−04	1
$\mathrm{F}10$	2.002E−02	1	5.450E−02	0	1.542E−03	1	4.671E−02	1

## Application of AZOA in solving various real-life engineering problems

In this part, the AZOA is evaluated on real life engineering problems, that present a variety of challenges, such as constraints, mixed integers, and so on. These constrained engineering optimization problems (in the case of minimization) can be represented as follows:

13 $$\begin{array}{*{20}l} {Minimize\,f\left( {\vec{z}} \right)\vec{z} \in R^{n} } \\ {Subject\; to\;\left\{ {\begin{array}{*{20}l} {g_{i} \left( {\vec{z}} \right) 0, } & { i = 1,2, \ldots m} \\ {h_{j} \left( {\vec{z}} \right) = 0,} & {j = 1,2, \ldots n} \\ \end{array} } \right.} \\ \end{array}$$

where ${g}_{i}$ and ${h}_{j}$ represent the inequality and equality constraints, respectively. ${R}^{n}$ denotes the $n$-dimensional vector space over real field. The goal of AZOA is to find the finest feasible solution that minimises the cost function $f(\overrightarrow{z})$ subject to constraints. To handle all these constraints in AZOA, the penalty function is used. The penalty function approach is applied to redefine the constrained engineering optimization problem. As a result, in Eq. ($14$) the optimization of these engineering problems applying AZOA is expressed as:

14 $$Minimize\;F\left( {\vec{z}} \right) = \left\{ {\begin{array}{*{20}l} {f\left( {\vec{z}} \right) } & {\vec{z} \in S < 0} \\ {f\left( {\vec{z}} \right) + \lambda \left( {\mathop \sum \limits_{i = 1}^{m} g_{i}^{2} \left( {\vec{z}} \right) + \mathop \sum \limits_{j = 1}^{k} h_{j}^{2} \left( {\vec{z}} \right)} \right), } & { \overrightarrow { z} \notin S \ge 0} \\ \end{array} } \right.$$

where $S$ denotes feasible search space. while applying such an approach, individuals who violate any constraint at any level are assigned a large function optimal value. As a result, throughout the optimization phase, the algorithm will automatically eliminate infeasible solutions. In this manner, by applying a penalty function, a constrained problem can be converted into an unconstrained problem.

### Solving tension or compression spring design problem using AZOA

**Figure 19:**
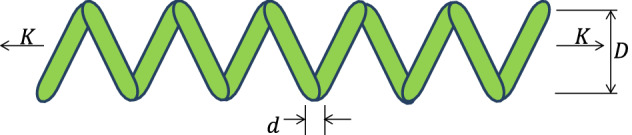
Tension or compression spring design problem.

The key idea behind this engineering design is to minimize the spring weight while considering three nonlinear and one linear inequality constraint. The geometric figure of the spring is seen in Fig. 19. This engineering problem has three continuous decision variables, including wire diameter ($d$ or ${z}_{1}$), mean coil diameter ($D$ or ${z}_{2}$), and the number of active coils ($K$ or ${z}_{3}$). Mathematical expression of the design has been presented as below:

15 $$\begin{array}{*{20}l} {{\text{Suppose}}\,\vec{z} = \left[ {z_{1} ,z_{2} , z_{3} } \right] = \left[ {K d D} \right]} \hfill \\ {Minimize\;f_{1} \left( {\vec{z}} \right) = \left( {z_{3} + 2} \right)z_{2} z_{1}^{2} } \hfill \\ {Subject to} \hfill \\ {g_{1} \left( {\vec{z}} \right) = 1 - \frac{{z_{2}^{3} z_{3} }}{{71785z_{1}^{4} }} \le 0} \hfill \\ {g_{2} \left( {\vec{z}} \right) = \frac{{4z_{2}^{2} - z_{1} z_{2} }}{{12566\left( {z_{2} z_{1}^{3} - z_{1}^{4} } \right)}} + \frac{1}{{5108z_{1}^{2} }} \le 0} \hfill \\ {g_{3} \left( {\vec{z}} \right) = 1 - \frac{{140.45z_{1} }}{{z_{2}^{2} z_{3} }} \le 0} \hfill \\ {g_{4} \left( {\vec{z}} \right) = \frac{{z_{1} + z_{2} }}{1.5} - 1 \le 0} \hfill \\ {0.05 \le z_{1} \le 2.00} \hfill \\ {0.25 \le z_{2} \le 1.30} \hfill \\ {2.00 \le z_{3} \le 15.0} \hfill \\ \end{array}$$

The outcomes of the newly proposed AZOA are compared to well-known metaheuristic algorithms that have successfully tackled this problem, including PSO, GSA, SSA, TSA, MVO, GWO, and LFD. The outcomes of this comparison are displayed in Table 17 and show that AZOA is able to generate effective solutions and design well.

**Table 17:** Outcomes of tension or compression design problem.

Algorithms	Optimum outcomes of variable			Optimal weight
	$d$	$D$	$N$	
AZOA	$5.13E{-}02$	$3.47E{-}01$	$1.19E{+}01$	$1.27{\varvec{E}}-02$
GWO	$5.17E{-}02$	$3.57E{-}01$	$1.13E{+}01$	$1.27E{-}02$
GSA	$5.03E{-}02$	$3.24E{-}01$	$1.35E{+}01$	$1.27E{-}02$
PSO	$5.17E{-}02$	$3.58E{-}01$	$1.12E{+}01$	$1.27E{-}02$
SSA	$5.12E{-}02$	$3.45E{-}01$	$1.20E{+}01$	$1.27E{-}02$
MVO	$5.00E{-}02$	$3.16E{-}01$	$1.42E{+}01$	$1.28E{-}02$
TSA	$5.22E{-}02$	$3.69E{-}01$	$1.06E{+}01$	$1.27E{-}02$
LFD	$5.17E{-}02$	$3.58E{-}01$	$1.12E{+}01$	$1.27E{-}02$

Significant values are in bold/italic.

### Solving pressure vessel design problem using AZOA

**Figure 20:**
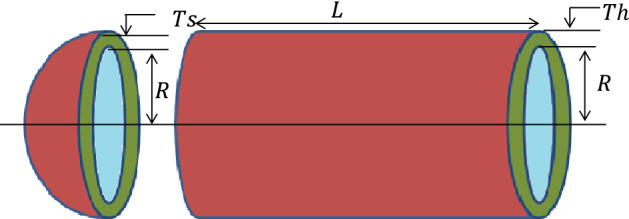
Pressure vessel design problem.

This design problem’s primary goal is to lower the price of a pressure vessel overall, which includes the costs of welding, forming, and materials, as illustrated in Fig. 20. This optimization design has four design variables as the thickness of the shell (${z}_{1}$ or $Ts$), the thickness of the head (${z}_{2}$ or $Th$), inner radius (${z}_{3}$ or $R$), and the length of the cylindrical portion of the vessel (${z}_{4}$ or $L$). In between this four-design variable, ${z}_{3}$ and ${z}_{4}$ are continuous, whereas ${z}_{1}$ and ${z}_{2}$ are discrete (integer multiplies of 0.0625 in). Mathematically, the pressure vessel is expressed as follows:

16 $$\begin{array}{*{20}l} {{\text{Consider}}\;\vec{z} = \left[ {z_{1} ,z, z_{3} , z_{4} } \right] = \left[ {T_{s} T_{h } R L} \right]} \hfill \\ {Minimize\;f_{2} \left( {\vec{z}} \right) = 0.6224z_{1} z_{3} z_{4} + 1.7781z_{2} z_{3}^{2} + 3.1661z_{1}^{2} z_{4} + 19.84z_{1}^{2} z_{3} ,} \hfill \\ {Subject \;to} \hfill \\ {g_{1} \left( {\vec{z}} \right) = - z_{1} + 0.0193z_{3} \le 0} \hfill \\ {g_{2} \left( {\vec{x}} \right) = - z_{3} + 0.00954z_{3} \le 0} \hfill \\ {g_{3} \left( {\vec{x}} \right) = - \pi z_{3}^{2} z_{4} - \left( \frac{4}{3} \right)\pi z_{3}^{2} + 1296000 \le 0} \hfill \\ {g_{4} \left( {\vec{x}} \right) = z_{4} - 240 \le 0} \hfill \\ {0 \le z_{1} \le 99} \hfill \\ {0 \le z_{2} \le 99} \hfill \\ {10 \le z_{3} \le 200} \hfill \\ {10 \le z_{4} \le 200} \hfill \\ \end{array}$$

The outcomes of the AZOA are compared to well-known metaheuristic algorithms, including PSO, GSA, SSA, TSA, MVO, GWO, and LFD. The outcomes of this comparison are displayed in Table 18, which illustrates that AZOA produced the best results in addressing this issue by lowering the total cost of the cylindrical pressure vessel.

**Table 18:** Outcomes of pressure vessel design problem.

Algorithms	Optimum outcomes of variable				Optimal cost
	$Ts$	$Th$	$R$	$L$	
AZOA	$7.84E{-}01$	$3.91E{-}01$	$4.06E{+}01$	$1.96E{+}02$	$\ {\mathbf{5}}.{\mathbf{88}} \varvec{E}+ {\mathbf{03}}$
GWO	$8.13E{-}01$	$4.35E{-}01$	$4.21E{+}01$	$1.77E{+}02$	$6.05E{+}03$
GSA	$1.13E{+}00$	$6.25E{-}01$	$5.60E{+}01$	$8.45E{+}01$	$8.54E{+}03$
PSO	$8.13E{-}01$	$4.38E{-}01$	$4.21E{+}01$	$1.77E{+}02$	$6.06E{+}03$
SSA	$7.78E{-}01$	$3.83E{-}01$	$4.03E{+}01$	$2.00E{+}02$	$5.88E{+}03$
MVO	$8.13E{-}01$	$4.38E{-}01$	$4.21E{+}01$	$1.77E{+}02$	$6.06E{+}03$
TSA	$1.03E{+}00$	$5.11E{-}01$	$5.32E{+}01$	$7.55E{+}01$	$6.52E{+}03$
LFD	$8.78E{-}01$	$4.34E{-}01$	$4.55E{+}01$	$1.39E{+}02$	$6.08E{+}03$

Significant values are in bold/italic.

### Solving welded beam design problem using AZOA

**Figure 21:**
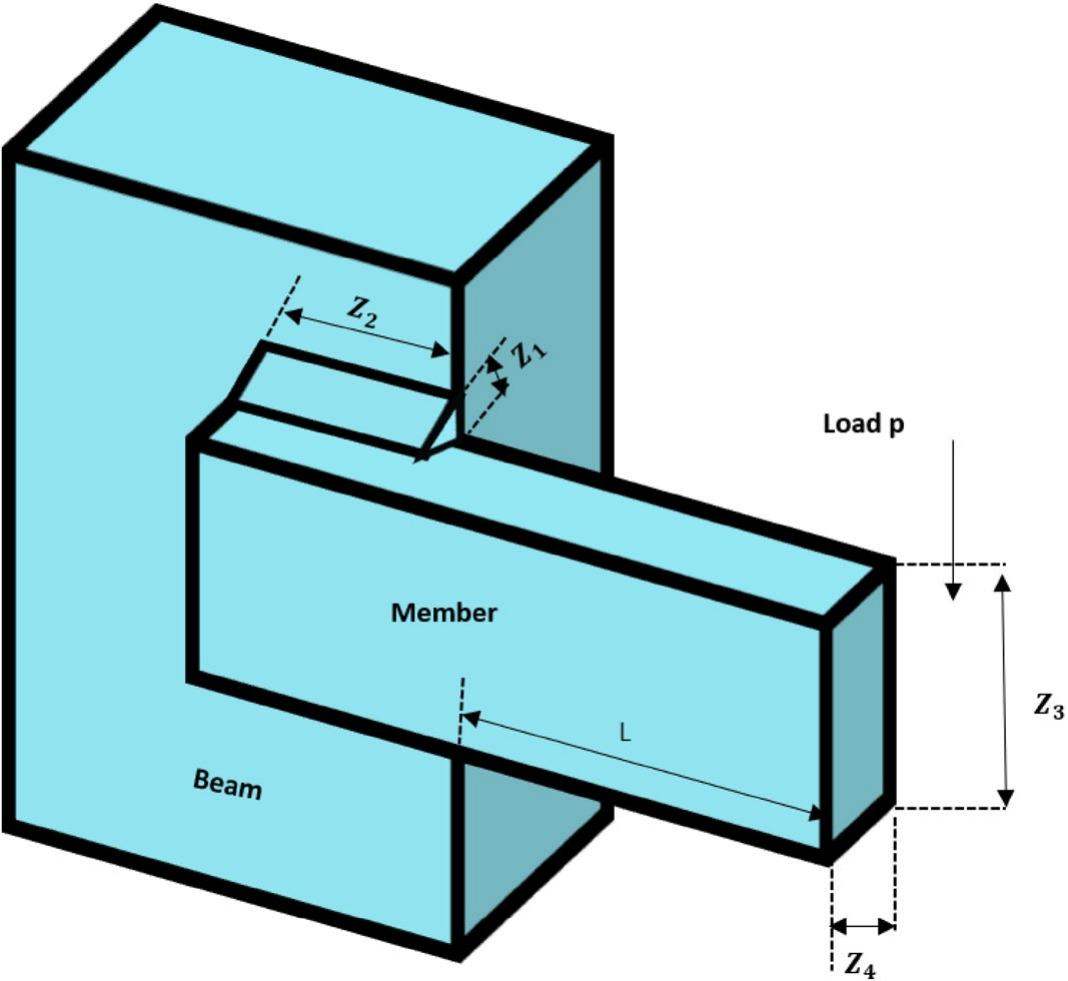
Welded beam design problem.

The aim of this design is to decrease the price of welded beams as much as possible. The diagram of the welded beam is shown in Fig. 21. This optimization problem contains 4 decision variables such as height of the bar $({z}_{3} or t)$, thickness of the bar $({z}_{4} or b)$, the thickness of the weld $({z}_{1} or h)$ and length bar connected portion, $( {z}_{2} or l).$ The following mathematical formula is defined to design this problem.

17 $$\begin{array}{*{20}l} {{\text{Consider}}\; \vec{z} = \left[ {z_{1} ,z_{2} , z_{3} , z_{4} } \right] = \left[ {h,l,t,b} \right]} \hfill \\ {Minimize\;f_{3} \left( {\vec{z}} \right) = 1.10471z_{1}^{2} z_{2} + 0.04811z_{3} z_{4} \left( {14 + z_{2} } \right)} \hfill \\ {Subject\;to\;g_{1} \left( {\vec{z}} \right) = \tau \left( {\vec{z}} \right) - \tau_{max} \le 0\; {\text{to}}} \hfill \\ {g_{2} \left( z \right) = \sigma \left( {\vec{z}} \right) - \sigma_{max} \le 0} \hfill \\ {g_{3} \left( {\vec{z}} \right) = \delta \left( {\vec{z}} \right) - \delta_{max} \le 0} \hfill \\ {g_{4} \left( {\vec{z}} \right) = z_{1} - z_{4} \le 0} \hfill \\ {g_{5} \left( {\vec{z}} \right) = P - P_{c} \left( {\vec{z}} \right) \le 0} \hfill \\ {g_{6} \left( {\vec{z}} \right) = 0.125 - z_{1} \le 0} \hfill \\ {g_{7} \left( {\vec{z}} \right) = 1.10471z_{1}^{2} + 0.04811z_{3} z_{4} \left( {14 + z_{2} } \right) - 5 \le 0} \hfill \\ {0.1 \le z_{1} \le 2} \hfill \\ {0.1 \le z_{2} \le 10} \hfill \\ {0.1 \le z_{3} \le 10} \hfill \\ {0.1 \le z_{4} \le 2} \hfill \\ \end{array}$$

where $$\tau \left( {\vec{z}} \right) = \sqrt {(\tau^{\prime } )^{2} + 2\tau^{\prime}\tau^{\prime \prime } \frac{{z_{2} }}{2R} + (\tau^{\prime \prime } )^{2} } , \tau^{\prime} = \frac{P}{{\sqrt 2 z_{1} z_{2} }}, \tau^{\prime \prime } = \frac{MR}{J}$$

$$M = P\left( {L + \frac{{z_{2} }}{2}} \right), R = \sqrt {\frac{{z_{2}^{2} }}{4} + \left( {\frac{{z_{1} + z_{3} }}{2}} \right)^{2} } , J = 2\left\{ {\sqrt 2 z_{1} z_{2} \left[ R \right]} \right\},$$

$$\sigma \left( {\vec{z}} \right) = \frac{6PL}{{z_{4} z_{3}^{2} }}, \delta \left( {\vec{z}} \right) = \frac{{6PL^{3} }}{{Ez_{3}^{2} z_{4} }},$$

$$P_{c} \left( z \right) = \frac{{4.013E\sqrt {\frac{{z_{3}^{2} z_{4}^{6} }}{36}} }}{{L^{2} }}\left( {1 - \frac{{z_{3} }}{2L}\sqrt{\frac{E}{4G}} } \right)$$

where $$P=6000lb, L=14in, E=30*{10}^{6}psi, G=12*{10}^{6}psi, {\tau }_{max}=\mathrm{13,600}psi, {\sigma }_{max}=\mathrm{30,000}psi, {\delta }_{max}=0.25in$$.

Table 19 shows the outcomes of a comparison of the AZOA with several metaheuristic algorithms employing the same penalty function. The outcomes demonstrate that the AZOA method performs superiorly in locating the optimal values for the welded beam design.

**Table 19:** Outcomes of welded beam design problem.

Algorithms	Optimum outcomes of variable				Optimal cost
	$h$	$l$	$t$	$b$	
AZOA	$4.69E{-}01$	$1.94E{+}00$	$5.72E{+}00$	$5.14E{-}01$	$1.72{\varvec{E}}+00$
GWO	$2.06E{-}01$	$3.48E{+}00$	$9.04E{+}00$	$2.06E{-}01$	$1.73E{+}00$
GSA	$1.82E{-}01$	$3.86E{+}00$	$1.00E{+}01$	$2.02E{-}01$	$1.88E{+}00$
PSO	$2.16E{-}01$	$3.47E{+}00$	$9.04E{+}00$	$2.66E{-}01$	$1.86E{+}00$
SSA	$2.06E{-}01$	$3.47E{+}00$	$9.04E{+}00$	$2.06E{-}01$	$1.72E{+}00$
MVO	$2.05E{-}01$	$3.47E{+}00$	$9.04E{+}00$	$2.06E{-}01$	$1.73E{+}00$
TSA	$1.96E{-}01$	$3.72E{+}00$	$9.03E{+}00$	$2.07E{-}01$	$1.75E{+}00$
LFD	$1.86E{-}01$	$3.91E{+}00$	$9.16E{+}00$	$2.05E{-}01$	$1.77E{+}00$

Significant values are in bold/italic.

### Solving Speed reducer design problem using AZOA

**Figure 22:**
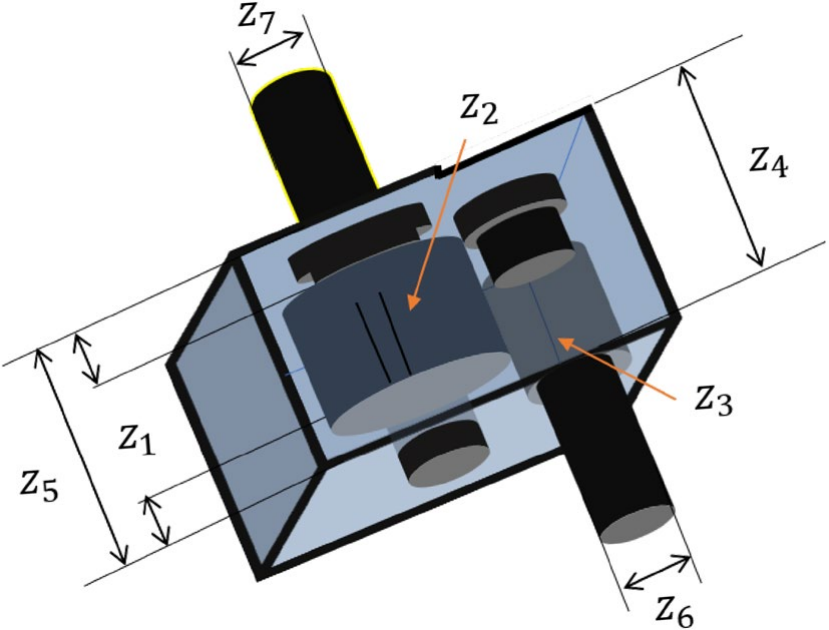
Speed reducer design problem.

In mechanical systems, one of the key pieces of the gearbox is the speed reducer, and it can be applied for numerous purposes. The weight of the speed reducer is to be reduced with 11 constraints in this optimization problem. This problem has seven variables such as face width $b\left({z}_{1}\right)$, module of teeth $m\left({z}_{2}\right)$, the number of teeth in the pinion $x\left({z}_{3}\right)$, length of the first shaft between bearings ${l}_{1}\left({z}_{4}\right)$, length of the second shaft between bearings ${l}_{2}\left({z}_{5}\right)$, the diameter of first shafts ${d}_{1}\left({z}_{6}\right)$, and the diameter of second shafts ${d}_{2}\left({z}_{7}\right)$ as revealed in Fig. 22. The mathematical formulation of the speed reducer problem is as follows.

18 $$\begin{array}{*{20}l} {{\text{Consider}}\;\vec{z} = \left[ {z_{1} z_{2} z_{3} z_{4} z_{5} z_{6} z_{7} } \right] = \left[ {b m x l_{1} l_{2} d_{1} d_{2} } \right],} \hfill \\ {Minimize\;f\left( {\vec{z}} \right) = 0.7854z_{1} z_{2}^{2} \left( {3.3333z_{3}^{2} + 14.9334z_{3} - 43.0934} \right) - 1.508z_{1} \left( {z_{6}^{2} + z_{7}^{2} } \right),} \hfill \\ {7.4777\left( {z_{6}^{2} + z_{7}^{2} } \right) + 0.7854\left( {z_{4} z_{6}^{2} + z_{5} z_{7}^{2} } \right),} \hfill \\ {Subject\; to\;g_{1} \left( {\vec{z}} \right) = \frac{27}{{z_{1} z_{2}^{2} z_{3} }} - 1 \le 0,} \hfill \\ {g_{2} \left( {\vec{z}} \right) = \frac{397.5}{{z_{1} z_{2}^{2} z_{3}^{2} }} - 1 \le 0,} \hfill \\ {g_{3} \left( {\vec{z}} \right) = \frac{{1.93z_{4}^{3} }}{{z_{2} z_{6}^{4} z_{3} }} - 1 \le 0,} \hfill \\ {g_{4} \left( {\vec{z}} \right) = \frac{{1.93z_{5}^{3} }}{{z_{2} z_{7}^{4} z_{3} }} - 1 \le 0,} \hfill \\ {g_{5} \left( {\vec{z}} \right) = \frac{{\sqrt {\left( {745z_{4} /z_{2} z_{3} } \right)^{2} + 16.9 \times 10^{6} } }}{{110z_{6}^{3} }} - 1 \le 0,} \hfill \\ {g_{6} \left( {\vec{z}} \right) = \frac{{\sqrt {\left( {745z_{5} /z_{2} z_{3} } \right)^{2} + 157.5 \times 10^{6} } }}{{85z_{7}^{3} }} - 1 \le 0,} \hfill \\ {g_{7} \left( {\vec{z}} \right) = \frac{{z_{2} z_{3} }}{40} - 1 \le 0,} \hfill \\ {g_{8} \left( {\vec{z}} \right) = \frac{{5z_{2} }}{{z_{1} }} - 1 \le 0,} \hfill \\ {g_{9} \left( {\vec{z}} \right) = \frac{{z_{1} }}{{12z_{2} }} - 1 \le 0,} \hfill \\ {g_{10} \left( {\vec{z}} \right) = \frac{{1.5z_{6} + 1.9}}{{z_{4} }} - 1 \le 0,} \hfill \\ {g_{11} \left( {\vec{z}} \right) = \frac{{1.1z_{7} + 1.9}}{{z_{5} }} - 1 \le 0,} \hfill \\ {2.6 \le z_{1} \le 3.6,} \hfill \\ {0.7 \le z_{2} \le 0.8,} \hfill \\ {z_{3} \in \left\{ {17, 18, 19, \ldots ., 28} \right\},} \hfill \\ {7.3 \le z_{4} ,} \hfill \\ {z_{5} \le 8.3,} \hfill \\ {2.9 \le z_{6} \le 3.9,} \hfill \\ {5 \le z_{7} \le 5.5} \hfill \\ \end{array} ,$$

Table 20 shows the results of the proposed algorithm and its comparison to other algorithms, such as GWO, GSA, PSO, SSA, TSA, MVO, and LFD on this problem. The simulation outcomes reveal that the proposed method, namely AZOA outperformed than other algorithms.

**Table 20:** Outcomes of Speed reducer design problem.

Algorithms	Optimum outcomes of variable							Optimal cost
	${z}_{1}$	${z}_{2}$	${z}_{3}$	${z}_{4}$	${z}_{5}$	${z}_{6}$	${z}_{7}$	
AZOA	3.5	0.7	17	7.3	7.715333	3.350215	5.286656	2994.472
GWO	3.506690	0.7	17	7.380933	7.815726	3.357847	5.286768	3001.288
GSA	3.600000	0.7	17	8.3	7.8	3.369658	5.289224	3051.120
PSO	3.500019	0.7	17	8.3	7.8	3.352412	5.286715	3005.763
SSA	3.50143	0.7	17	7.3	7.8	3.33421	5.26536	2994.2472
MVO	3.508502	0.7	17	7.392843	7.816034	3.358073	5.286777	3002.928
TSA	3.50274	0.7	17	7.3	8.3	3.46274	5.29886	3045.8524
LFD	3.50774	0.7	17	7.3	8.3	3.4738	5.2967	3005.753

### Solving Gear train design problem using AZOA

**Figure 23:**
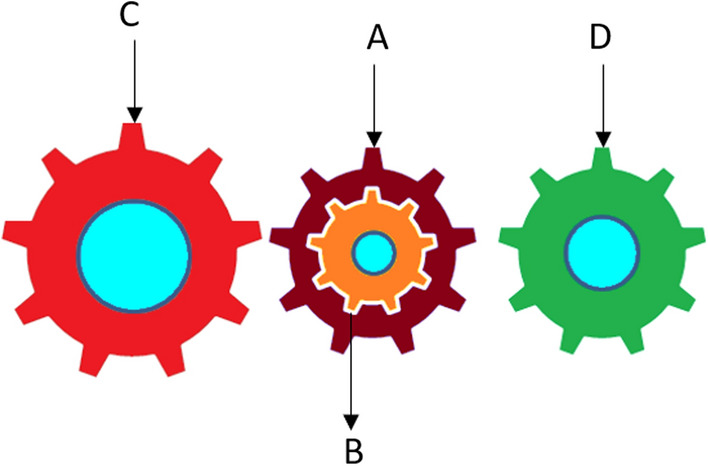
Gear train design problem.

The primary purpose of this structural problem is to minimize the gear ratio for the making of compound gear train as depicted in Fig. 23.

The objective is to determine the optimal number of teeth for four gears of a train in order to minimize gear ratio. The design variable which is same as the number of teeth of the gears are: ${n}_{A}\left({z}_{1}\right)$, ${n}_{B}\left({z}_{2}\right)$, ${n}_{C}\left({z}_{3}\right)$, and ${n}_{D}\left({z}_{4}\right)$. The mathematical formulation of the gear train design problem is as follows.

19 $${\text{Consider}}\;\vec{z} = \left[ {z_{1} z_{2} z_{3} z_{4} } \right] = \left[ {n_{A} n_{B} n_{C} n_{D} } \right],$$

$$Minimize\;f\left( {\vec{z}} \right) = \left( {\frac{1}{6.931} - \frac{{z_{3} z_{2} }}{{z_{1} z_{4} }}} \right)^{2} ,$$

$${\text{Variable}}\;{\text{range}}\;z_{1} ,\;z_{2} ,\;z_{3} ,\;z_{4} \in \left\{ {12, 13, 14, \ldots , 60} \right\}.$$

The outcomes of the proposed algorithm, namely AZOA, and its comparison to the other metaheuristic algorithms such as MFO^35^, ABC^76^, PSO^32^, CS^77^, MVO^25^, TSA^41^ and WOA^36^ are provided in Table 21. The simulation results in Table 21 show that AZOA outperforms the compared algorithm.

**Table 21:** Outcomes of Gear train design problem.

Algorithms	Optimum outcomes of variable				Optimal cost
	${n}_{A}\left({z}_{1}\right)$	${n}_{B}\left({z}_{2}\right)$	${n}_{C}\left({z}_{3}\right)$	${n}_{D}\left({z}_{4}\right)$	
AZOA	60	17.52	12	24.29	0
MFO	43	19	16	49	2.7009E−012
ABC	49	16	19	43	2.7009E−012
PSO	52	15	29	58	2.35764E−09
CS	43	16	19	49	2.7009E−012
MVO	43	16	19	49	2.7009e−012
TSA	60	16.8636	26.7906	52.1895	3.8701E−12
WOA	47	12	13	23	9.92157E−10

### Solving three bar truss design problem using AZOA

**Figure 24:**
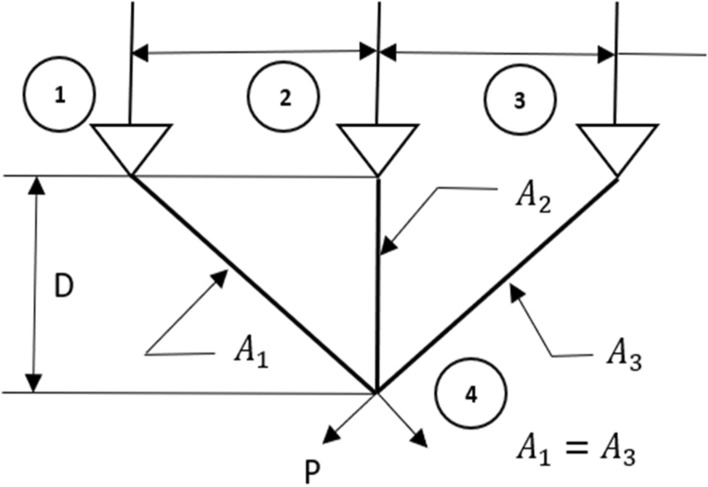
Three bar truss design problem.

The goal of truss design is to reduce the weight of the bar constructions. Figure 24 presents the graphical structure of this problem. The volume of a statically loaded 3-bar truss must be reduced while stress $\left(\upsigma \right)$ constraints on each truss member are maintained. The main aim is to find the best cross-sectional areas, ${\mathrm{A}}_{1}\left({\mathrm{z}}_{1}\right)$ and ${\mathrm{A}}_{2}\left({\mathrm{z}}_{2}\right)$. The mathematical formulation of this design problem is as follows.

20 $$\begin{array}{*{20}l} {{\text{Consider}}\;\vec{z} = \left[ {z_{1} z_{2} } \right] = \left[ {A_{1} A_{2} } \right],} \hfill \\ {Minimize,\,f\left( {\vec{z}} \right) = \left( {2\sqrt 2 z_{1} + z_{2} } \right) \times l,} \hfill \\ {Subject to,\;g_{1} \left( {\vec{z}} \right) = \frac{{\sqrt 2 z_{1} + z_{2} }}{{\sqrt 2 z_{1}^{2} + 2z_{1} z_{2} }}P - \sigma \le 0,} \hfill \\ {g_{2} \left( {\vec{z}} \right) = \frac{{z_{2} }}{{\sqrt 2 z_{1}^{2} + 2z_{1} z_{2} }}P - \sigma \le 0,} \hfill \\ {g_{3} \left( {\vec{z}} \right) = \frac{1}{{\sqrt 2 z_{2} + z_{1} }}P - \sigma \le 0,} \hfill \\ {l = 100 cm,\;P = 2kN/cm^{3} ,\;\sigma = 2kN/cm^{3} } \hfill \\ {{\text{Variable}}\;{\text{range}}\;0 \le z_{1} ,\;z_{2} \le 1} \hfill \\ \end{array}$$

Table 22 shows the outcomes of the proposed algorithm and its comparison to other algorithms, such as GOA^38^, MBA^79^, PSO-DE^78^, SSA^37^, MVO^25^, TSA^41^, and AO^43^ on this problem. The outcomes demonstrate that the proposed method, namely AZOA outperformed than compared algorithms.

**Table 22:** Outcomes of three-bar truss design problem.

Algorithms	Optimal values of variable		Optimum weight
	${\mathrm{A}}_{1}\left({\mathrm{z}}_{1}\right)$	${\mathrm{A}}_{2}\left({\mathrm{z}}_{2}\right)$	
AZOA	0.7885471	0.408610	263.89585540
GOA	0.7888975	0.407619	263.89588149
MBA	0.7885650	0.408559	263.89585
PSO-DE	0.7886751	0.408248	263.89584
SSA	0.7886654	0.408275	263.8958434
MVO	0.7886027	0.408453	263.8958499
TSA	0.78928	0.40665	263.9076
AO	0.7926	0.3966	263.8684

### Application of AZOA for optimal placement of wind turbines in wind farms

Wind energy is the electrical energy generated by harnessing the wind via windmills or wind turbines. It is one of the most prominent types of renewable energy sources as it is plentiful and present everywhere. This energy when used appropriately, can assist us with creating a lot of electricity. Wind energy has recently acquired popularity in response to rising electricity demand. A wind farm's total energy output can be maximized by employing the wind turbines in the best possible position. Positioning a wind turbine in a wind farm is a difficult operation since the aspects like the wake loss caused by upstream wind turbines to the downstream wind turbines must be taken into account. Minimizing the wake loss to increase output power poses a challenge for various optimization algorithms applied to this layout optimization problem. Hence, in this section, the AZOA algorithm is employed to find the optimal location of wind turbines and maximize the total power output with the minimum cost per kilowatt. Two different case studies are performed such as: constant wind speed (CWS) with variable wind direction (VWD) and variable wind speed (VWS) with variable wind direction (VWD). The experimental outcomes are compared with studies performed employing L-SHADE^80^, GA^81^, GA^82^, GWO^83^, BPSO-TVAC^84^, RSA^85^, and SBO^86^. The mathematical modelling of the wind farm layout problem is addressed as follows.

**Figure 25:**
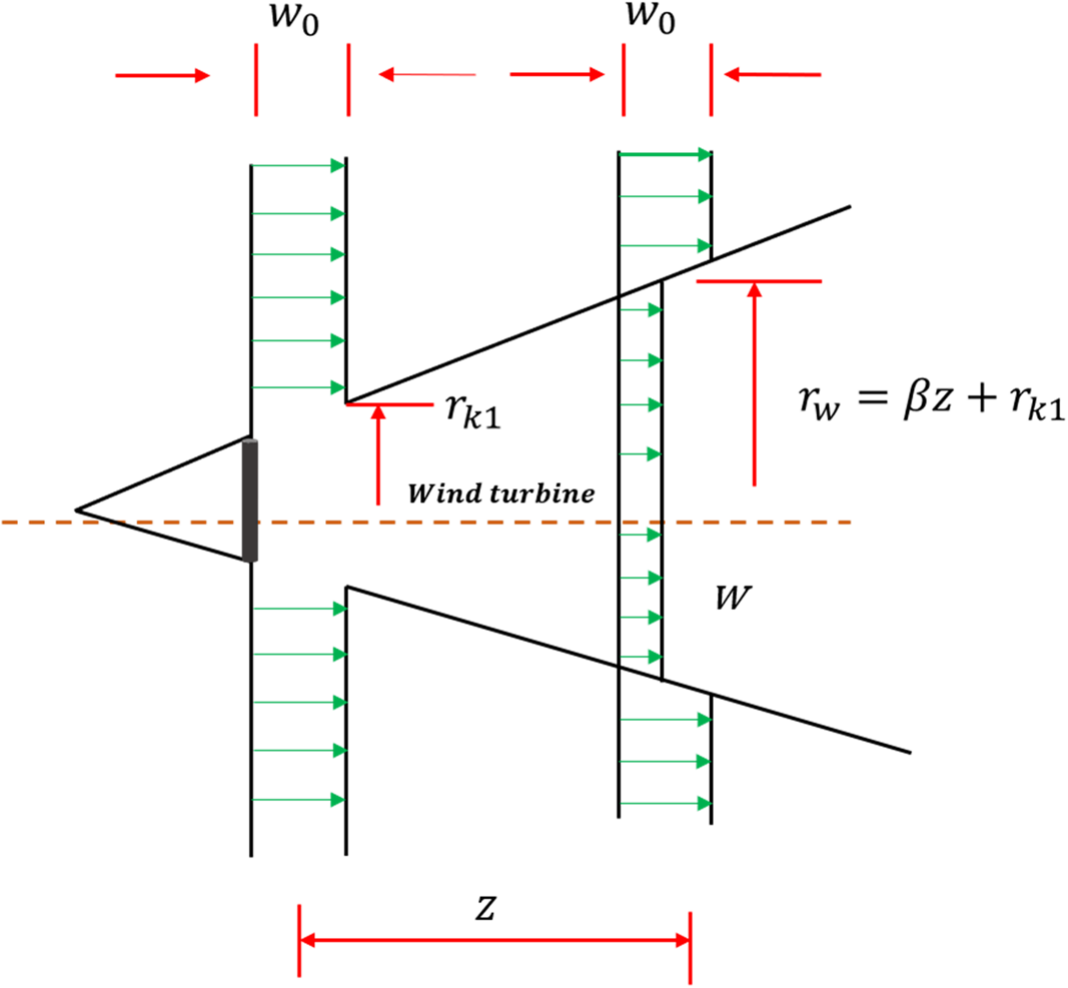
Jensen linear wake model.

As wind passes through a turbine, the speed of the wind drops and the strength of the turbulence increases, leaving a wake behind the turbine. Not only does the wake keep moving downstream, but it inflates laterally as well. Turbines placed downstream create less power due to the wake effect. The Jensen linear wake decay model^87,88^ is used in this study for the calculation of wind velocity in the wake zone. Figure 25 depicts the schematic of the linear wake model. The speed of the wind in the wake zone is estimated using the assumption that momentum is preserved in the wake. The wind speed in the wake region is given by:

21 $$w={w}_{0}\left[1-\frac{2a}{\left(1+\beta \frac{{z}_{i,j}}{{r}_{k1}}\right)}\right]$$

22 $$a=\frac{1-\sqrt{1-{c}_{r}}}{2}$$

23 $${r}_{k1}={r}_{k}\sqrt{\frac{1-a}{1-2a}}$$

24 $${\beta }_{k}=\frac{0.5}{ln\left(\frac{{h}_{k}}{{z}_{0}}\right)}$$

25 $${C}_{r}=4a(1-a)$$

where w denotes the wake effect, ${w}_{0}$ denotes the original wind speed without taking into account any wake impact, *a* denotes the axial induction factor, ${\beta }_{k}$ denotes the entrainment constant in relation to the *kt*ℎ turbine, ${z}_{i,j}$ is the distance amongst the ${i}$th and the ${j}$th turbine, ${r}_{k1}$ is the downstream rotor radius, ${h}_{k}$ is the hub height of the ${k}$th turbine, ${z}_{0}$ denotes the surface roughness of the wind farm, ${C}_{r}$ is the coefficient of thrust of the wind turbine rotor.

When a single turbine encounters numerous wakes, the combined wake's kinetic energy is believed to be equivalent to the total of the kinetic energy deficits.

The resultant velocity of ${i}$th turbine downstream of ${N}_{x}$ turbines is given by:

26 $${w}_{i}={w}_{0}\left[1-\sqrt{{\sum }_{k=1}^{{N}_{x}}{\left(1-\frac{{w}_{ik}}{{w}_{0}}\right)}^{2}}\right]$$

where ${w}_{ik}$ denotes the velocity of wind of the ${i}$th turbine under the impact of the ${k}$th turbine. For the linear wake model, the wake region is conical, and the radius of the wake zone is defined as the wake influence radius determined by:

27 $${r}_{w}=\beta z+{r}_{k1}$$

Power output from ${i}$th turbine in $kW$ is given by:

28 $${P}_{i}=0.5*\rho *\pi {*{r}_{k}}^{2}*{{w}_{i}}^{3}*{C}_{p}/100 kW$$

where $\rho$ represents the air density and ${C}_{p}$ is the efficient of the rotor.

The total power output of a windfarm with $N$ turbines is calculated by Eq. (29).

29 $${p}_{x}={\sum }_{m=0}^{360}{\sum }_{i=1}^{N}{f}_{m}{P}_{i}({w}_{i})$$

where

30 $${P}_{i}=0.3*{{w}_{i}}^{3}$$

Cost per $kW$ of the output power is calculated by:

31 $$Cost per kW=\frac{cost}{{p}_{x}}$$

where

32 $$Cost=N (\frac{2}{3}+\frac{1}{3}{e}^{-0.00174{N}^{2}})$$

The efficiency of the windfarm is calculated by the formula:

33 $$Efficiency=\frac{{\sum }_{m=0}^{360}{\sum }_{i=1}^{N}{f}_{m}{P}_{i}({w}_{i})}{{\sum }_{m=0}^{360}{\sum }_{i=1}^{N}{f}_{m}{P}_{i,max}({w}_{i, max})}$$

where ${P}_{i,max}$ represents the maximum power output of the ${i}$th turbine as a function of the maximum wind speed ${w}_{i, max}$ if there were no wake effect and ${f}_{m}$ represents the probability of a particular wind speed from a specific direction.

**Figure 26:**
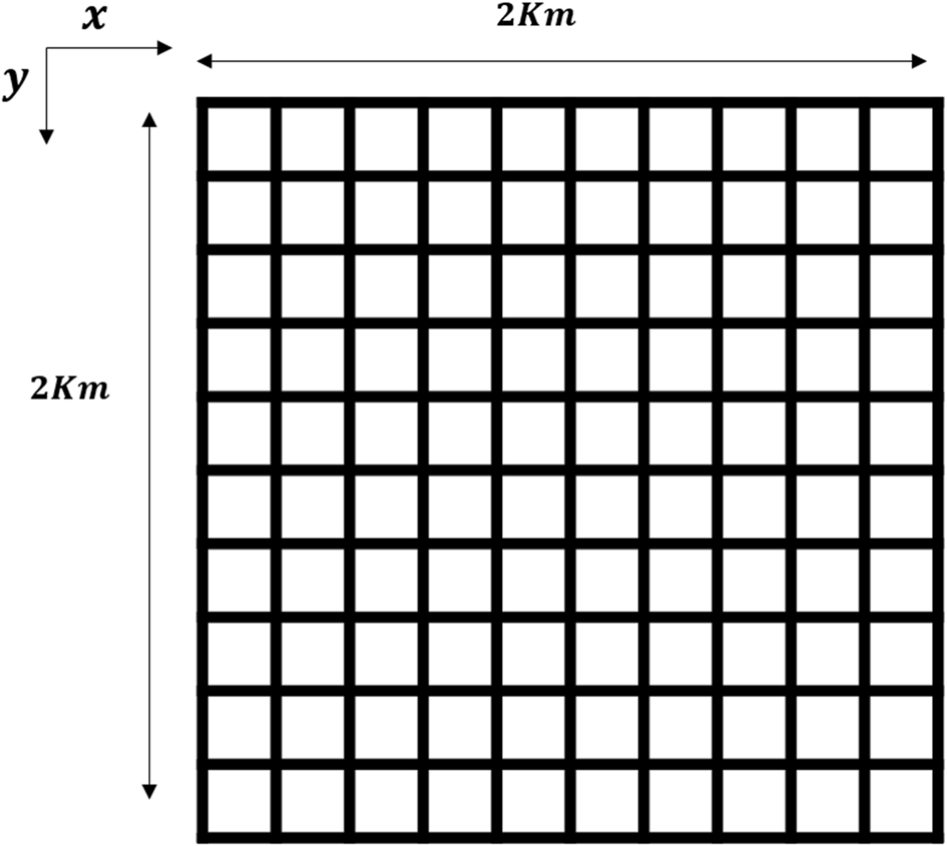
Wind farm topology.

This work is based on an analysis of a 10 × 10 square wind farm with 100 possible places for wind turbines. All the wind turbines were deployed in the middle of the cubicle. The dimension of each cubicle is 200 m, as represented in Fig. 26. The selection of cubicle, which was equal to the diameter of the rotor, prevented the wake from striking the other turbines when it was placed in a column with another adjacent column. Parameters for the wind farm employed in this study are listed in Table 23. The proposed method, namely the AZOA algorithm, is implemented in both the case studies (CWS with VWD and VWS with VWD), and the outcomes are compared with other existing algorithms, including L-SHADE^80^, GA^81^, GA^82^, GWO^83^, BPSO-TVAC^84^, RSA^85^, and SBO^86^. Each algorithm is modelled employing a population size of 200 and a maximum number of 100 iterations. The upper bound and lower bound are assigned as 1 and 0, respectively, while the size of the problem is assigned to 100.

**Table 23:** Numerical data of wind turbines in the experiment.

Parameters	Value
Hub height $( {h}_{k})$	60 m
Thrust coefficient $({C}_{r})$	0.88
Rotor diameter $(2{r}_{k})$	40 m
Air density $(\rho )$	1.2254 kg/m^3^
Surface roughness of wind farm $({z}_{0})$	0.3 m
Rotor efficiency $({C}_{p})$	0.4

**Figure 27:**
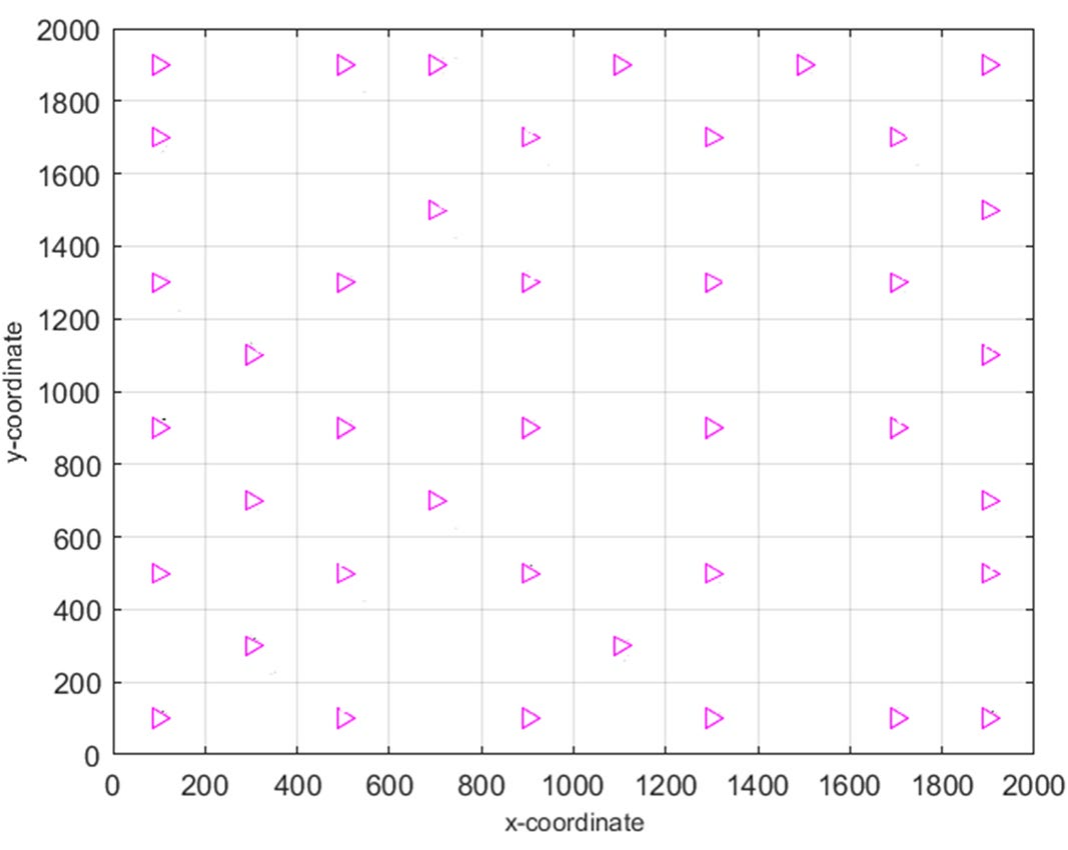
Optimal wind farm configuration by AZOA for CWS with VWD.

In the first case, a CWS of 12 m per second was assumed with an equal chance of wind flow from each direction by investigating 36 angles ranging from ${0}^{^\circ }$ to ${360}^{^\circ }$ degrees in ${10}^{^\circ }$ increments. The proposed AZOA is employed in this case, and the outcomes of the AZOA algorithm and its comparison to the other metaheuristic algorithms are provided in Table 24. From Table 24, it is observed that AZOA outperforms the compared algorithm for the same objective function. Figure 27 depicts the optimal wind farm configuration identified by AZOA. The proposed AZOA algorithm generates an annual power output of 17,920 kW from 40 turbines at a cost per kW of 0.0015340 and an efficiency of 86.42%.

**Table 24:** Outcomes obtained in the case of CWS with VWD.

Algorithms	No. of turbines	Power (kW)	Cost/kW	Efficiency
AZOA	40	17,920	**0.0015340**	86.42%
L-SHADE^80^	40	17,920	0.0015341	86.42%
GA^81^	19	9245	0.0017371	93.86%
GA^82^	39	17,220	0.001567	85.17%
GWO^83^	40	17,817	0.0015430	86%
BPSO-TVAC^84^	35	15,796	0.0015648	87.06%
RSA^85^	39	17,406	0.0015470	NA
SBO^86^	40	17,254	0.0015930	83%

Significant values are in bold.

**Figure 28:**
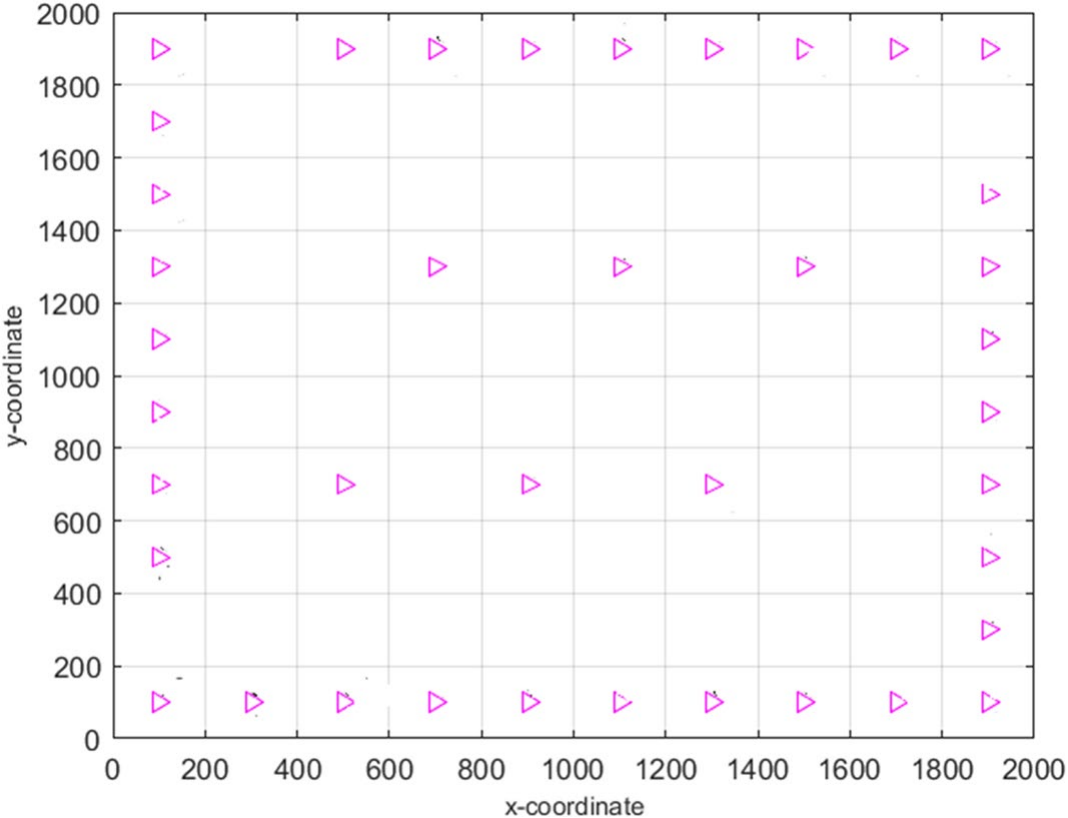
Optimal wind farm configuration by AZOA for VWS with VWD.

To verify the efficiency of the proposed method for optimal placement of a wind farm in case 2, VWS and VWD are assumed. In this case, 8 m/s, 12 m/s, and 17 m/s with 36 angles ranging from 0° to 360° degrees in 100° increments are considered. The proposed AZOA is employed in this case, and the outcomes of the AZOA algorithm and its comparison to the other metaheuristic algorithms are provided in Table 25. From Table 25, it is observed that AZOA outperforms the compared algorithm for the same objective function. Figure 28 depicts the optimal wind farm configuration identified by AZOA. The proposed AZOA algorithm generates an annual power output of 32,556 kW from 39 turbines at a cost/kW of 0.00083218 and an efficiency of 86.78%.

**Table 25:** Outcomes obtained in the case of VWS with VWD.

Algorithms	No. of turbines	Power (kW)	Cost/kW	Efficiency
AZOA	39	32,556	**0.00083218**	86.78%
L-SHADE^80^	39	32,351	0.0008322	86.68%
GA^81^	15	13,460	0.0009941	94.62%
GA^82^	39	31,850	0.000840	86%
GWO^83^	46	36,433	0.0008523	82.76%
BPSO-TVAC^84^	38	31,498	0.000837	86%
RSA^85^	39	32,096	0.000839	NA
SBO^86^	40	32,501	0.000847	85%

Significant values are in bold.

Finally, the obtained outcomes reveal the efficiency and validity of the AZOA algorithm in optimally configuring turbines in a wind farm for both case studies, as the algorithm provided better outcomes when compared to other algorithms.

### Solving the economic load dispatch (ELD) problem using AZOA

In the area of power systems, the ELD is one of the highlighted problems attracted by the researchers. The primary goal of the problem is to allocate required power among available generator units as efficiently as possible in order to reduce overall fuel costs while maintaining load demand and all power units' various operational constraints^89,90^. The overall fuel cost of the generators is generally expressed using a quadratic function as follows:

34 $${F}_{i}\left({p}_{i}\right)={u}_{i}*{{p}_{i}}^{2}+{v}_{i}*{p}_{i}+{w}_{i}$$

where ${u}_{i},v, {w}_{i}$ are the cost coefficients of ${i}$th generator, ${F}_{i}$ is the cost of ${i}$ generator, ${p}_{i}$ is the generated power of ${i}$th generator and $N$ is the total generators. Typically, the aggregate supply of power produced by the generators is more than sufficient to satisfy both the required amount and the entire transmission line loss. Thus, it is necessary to satisfy the following equality criteria:

35 $${p}_{d}+{p}_{l}=\sum_{i=1}^{N}{p}_{i}$$

Here, ${p}_{d}$ and ${p}_{l}$ represent the demand and the total line transmission loss, respectively. The Kron's loss formula is employed for determining the transmission loss in the form shown below.

36 $${p}_{l}=\sum_{i=0}^{N}\sum_{j=1}^{N}{p}_{i}{B}_{ij}{p}_{ij}+\sum_{i=1}^{N}{B}_{i0}{p}_{i}+{B}_{00}$$

In this context, the $B$ terms ${B}_{ij}, {B}_{i0}$ and ${B}_{00}$ are referred to as the loss coefficients. The overall power produced by the generators is circumscribed by their respective maximum active power ${p}_{max}$ and the minimum power ${p}_{min}$ because of the capabilities and limitations on the generators. As a result, each generator needs to comply with the criteria below.

37 $${p}_{min}\le {p}_{i}\le {p}_{max}$$

Let ${F}_{i}$ epitomize as the cost of producing energy at ${i}$th generator. Then, the total cost $C$ is demarcated as $\sum_{i=1}^{N}{F}_{i}$. The cost function is primarily influenced by the actual generated power ${p}_{i}$. Therefore, ${p}_{i}$ is the only variables used to estimate the individual cost ${F}_{i}$ of the generating units and the total cost $C$ can be articulated as $\sum_{i=1}^{N}{F}_{i}\left({p}_{i}\right)$.

38 $$\begin{array}{*{20}l} {{\text{Minimum}}\;C = \mathop \sum \limits_{i = 1}^{N} F_{i} \left( {p_{i} } \right)} \\ {{\text{Subject to}}\;\mathop \sum \limits_{i = 1}^{N} p_{i} = p_{d} + p_{l} } \\ \end{array}$$

**Figure 29:**
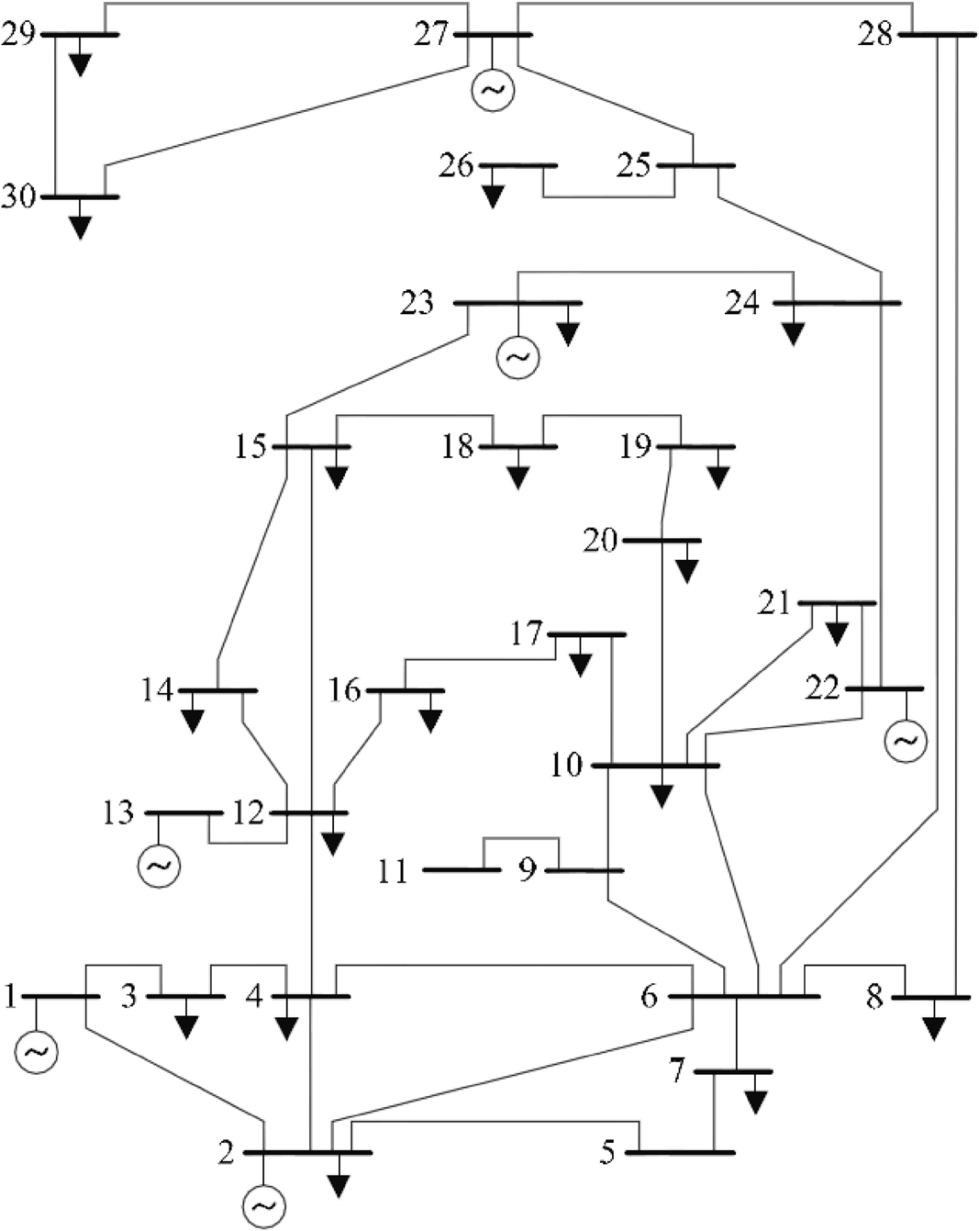
Structure of a 30 bus IEEE system.

The structure of an IEEE-30 system with six generators is illustrated in Fig. 29. In Table 26, the cost coefficients $$({u}_{i}$$, $${v}_{i}$$ and $${w}_{i})$$ and the limit constraints ($${p}_{imin}$$, $${p}_{imax})$$ of the generators are reported. In Table 27, the coefficient matrix B for the specified system is provided. The stated problem is solved through AZOA to determine the most cost-effective load dispatch for multiple distinct loads of 600 MW, 700 MW, and 800 MW. Several well-known algorithms are compared to AZOA, including lambda iteration^91^ and quadratic programming^92^, GA^93^, and, PSO^94^. Tables 28, 29 and 30 demonstrate the algorithm comparison results for needs of 600 MW, 700 MW, and 800 MW, respectively. From these Tables, it is observed that the proposed algorithm AZOA provided the best fuel cost among all the compared algorithms.

**Table 26:** The cost coefficients with the limits of the generators.

No	${u}_{i}$	${v}_{i}$	${w}_{i}$	${p}_{imin}$	${p}_{imax}$
1	0.15240	38.53973	756.79886	10	125
2	0.10587	46.39655	451.32513	10	150
3	0.02803	40.39655	1049.9977	35	225
4	0.03546	38.32782	1243.5311	35	210
5	0.02111	36.32782	1658.5596	130	325
6	0.01799	38.27041	1356.6592	125	315

**Table 27:** Loss coefficient matrix B.

0.000140	0.000017	0.000015	0.000019	0.000026	0.000022
0.000017	0.000060	0.000013	0.000016	0.000015	0.000020
0.000015	0.000013	0.000065	0.000017	0.000024	0.000019
0.000019	0.000016	0.000017	0.000071	0.000030	0.000025
0.000026	0.000015	0.000024	0.000030	0.000069	0.000032
0.000022	0.000020	0.000019	0.000025	0.000032	0.000085

**Table 28:** Comparison of cost in different methods in solving 6-unit system of 600 MW.

No.	Lambda iteration^90^	Quadratic programming^91^	GA^92^	PSO^93^	AZOA
${p}_{1}$	23.86	23.90	22.8	23.86	23.85
${p}_{2}$	10	10	10	10	10
${p}_{3}$	95.64	95.63	103.3	95.64	95.98
${p}_{4}$	100.07	100.70	98.9	100.07	100.68
${p}_{5}$	202.83	202.82	194.23	202.83	202.50
${p}_{6}$	181.19	182.02	187.5	181.19	181.19
Total	614.23	615.07	616.73	614.23	614.23
Loss	14.23	15.07	14.2	14.23	14.230
Cost	32,094.7	32,096.5	32,098.6	32,094.7	**32,094.7**

Significant values are in bold.

**Table 29:** Comparison of cost in different methods in solving 6-unit system of 700 MW.

No.	Lambda iteration^90^	Quadratic programming^91^	GA^92^	PSO^93^	AZOA
${p}_{1}$	28.29	28.33	29.09	28.2	28.29
${p}_{2}$	10	10	10	10.0	10
${p}_{3}$	118.96	118.95	116.64	118.53	119.29
${p}_{4}$	118.67	118.67	123.43	118.53	118.82
${p}_{5}$	230.76	230.75	226.4	230.2	230.80
${p}_{6}$	212.74	212.80	213.7	214.16	212.19
Total	719.43	719.50	719.23	719.62	719.42
Loss	19.43	19.50	19.4	19.4	19.42
Cost	36,912.2	36,914.01	36,913.7	36,912.2	**36,912.2**

Significant values are in bold.

**Table 30:** Comparison of cost in different methods in solving 6-unit system of 800 MW.

No.	Lambda iteration^90^	Quadratic programming^91^	GA^92^	PSO^93^	AZOA
${p}_{1}$	32.58	32.63	32.5	32.58	32.58
${p}_{2}$	14.48	14.48	12.4	14.48	14.48
${p}_{3}$	141.54	141.54	140.51	141.54	141.54
${p}_{4}$	136.04	136.04	136.2	136.04	136.04
${p}_{5}$	257.66	257.65	258.28	257.66	257.66
${p}_{6}$	243.00	243.00	245.3	243.00	242.99
Total	825.33	825.34	825.19	825.33	825.33
Loss	25.76	25.34	25.44	25.76	25.33
Cost	41,896.7	41,898.4	41,925.2	41,896.7	**41,896.7**

Significant values are in bold.

## Conclusion and future work

This study has developed a novel bio-inspired meta-heuristic algorithm, namely AZOA, inspired by the social behaviour of American zebras in the wild. The main inspiration for this proposed algorithm is the unique and fascinating social character and leadership exercise of American zebras in the wild, which navies the baby zebras to leave the herd before maturity and join a separate herd with no family relations. This process of leaving the group prevents the zebra parents from breeding with their offspring to guarantee diversity in AZOA. Similarly, the convergence is assured by the leadership exercise in American zebras to direct the speed and direction of the group. The proposed AZOA concept has been modelled and designed in five simple phases for easy implementation and superior performance. To evaluate the efficiency of the AZOA algorithm, the CEC-2005, CEC-2017, and CEC-2019 benchmark functions are taken into consideration while compared with several existing and latest outstanding evolutionary algorithms. The simulation results and statistical analysis reveal that AZOA is capable of attaining the optimal solutions for maximum benchmark functions while maintaining a good balance between exploration and exploitation. Additionally, sensitivity analysis has been employed to access the performance of the proposed AZOA. Furthermore, the implementation of AZOA in solving several engineering design optimization problems ensured the robustness of the proposed algorithm in real-world optimization problems. Although the proposed AZOA has offered superior performance in most of the benchmark functions examined in this article, the superiority of AZOA is not remarkable when handling some multimodal and composite problems against the classical algorithms, and it also attained mediocre results against contemporary algorithms such as FFA, MGO, AVOA, and GTO. Hence, several modifications, such as the implementation of learning operators, the introduction of adaptive weight parameters, and the design of the binary and multimodal versions, are the scope of future research work of the AZOA algorithm.

## Data Availability

All data generated or analysed during this study are included in this article.
